# Iridium(I)– and Rhodium(I)–Olefin Complexes Containing an α-Diimine Supporting Ligand

**DOI:** 10.1021/acs.organomet.2c00036

**Published:** 2022-04-05

**Authors:** James Kovach, Suzanne R. Golisz, William W. Brennessel, William D. Jones

**Affiliations:** Department of Chemistry, University of Rochester, Rochester, New York 14627, United States

## Abstract

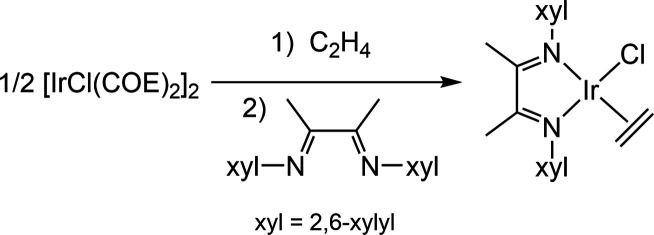

Iridium(I) complexes of the type IrX(olefin)(α-diimine) (α-diimine = 1,4-bis(2,6-xylyl)-2,3-dimethyl-1,4-diaza-1,3-butadiene; X = Cl, I, Me, O_2_CCF_3_; olefin = ethylene, cyclooctene (COE)) were synthesized from the readily available precursor [IrCl(COE)_2_]_2_. These complexes display unusual ^1^H NMR spectra and have large UV–vis extinction coefficients. NOESY and HSQC NMR experiments were used to provide rigorous NMR spectral assignments, and IrCl(C_2_H_4_)(α-diimine), **1**, and IrCl(COE)(α-diimine), **4**, were structurally characterized by X-ray crystallography. The related rhodium complex [RhCl(α-diimine)]_2_, **6**, was also synthesized and characterized by NMR and X-ray crystallography. **6** was observed to be in equilibrium with RhCl(C_2_H_4_)(α-diimine), **7**, under an ethylene atmosphere.

## Introduction

Saturated hydrocarbons make up the major component of petroleum and natural gas.^1^ Since the C–C and C–H bonds of which saturated hydrocarbons are comprised are relatively inert,^2^ these feedstocks are primarily used as a fuel source. Consequently, new catalysts for the efficient, direct functionalization of C–H bonds have been sought for decades. In this regard, the α-diimine ligand scaffold (also known as diazabutadiene, DAB) has found many applications in C–H activation and other catalysis.

Shilov was among the first to develop a homogeneous alkane oxidation catalyst system using Pt^IV^,^3,4^ and ever since related research was focused on understanding the fundamental processes of the Shilov system and making modifications to improve Shilov-style catalyst performance.^5^ Bercaw, Labinger, and Tilset have developed a cationic α-diimine platinum(II) complex that shares many of the same features of Shilov’s catalyst, which C–H activates benzene^6^ and substituted arenes (eq 1).^7^ More recently, Gunnoe found that an α-diimine rhodium complex catalyzes the oxidative coupling of ethylene with benzene in the presence of Cu(II) oxidant (eq 2)^8^ or even using only O_2_.^9^ Rhodium and iridium α-diimine complexes have also been found to be active for CO_2_ reduction to formate,^10^ alkyne amination,^11^ and vinylarene borylation (eq 3).^12^ Additionally, α-diimine complexes have been seen to react with H_2_ (oxidative addition) and O_2_ (peroxide formation).^13^ Inspired by the above reactivities, we sought to prepare organometallic iridium(I) complexes containing a labile olefin ligand and the α-diimine ligand 1,4-bis(2,6-xylyl)-2,3-dimethyl-1,4-diaza-1,3-butadiene, which has literature precedent^6^ for use in a C–H activation complex.
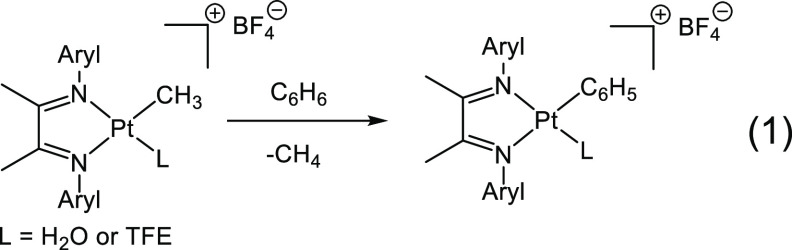
1
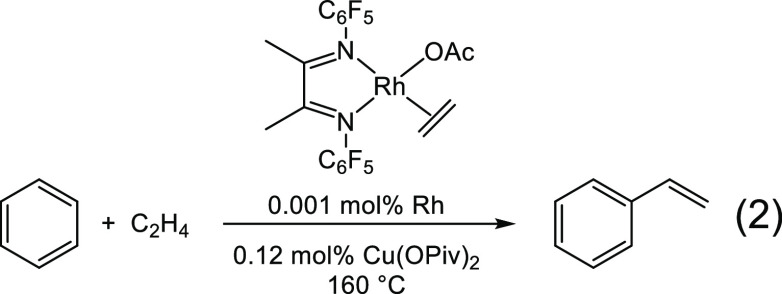
2
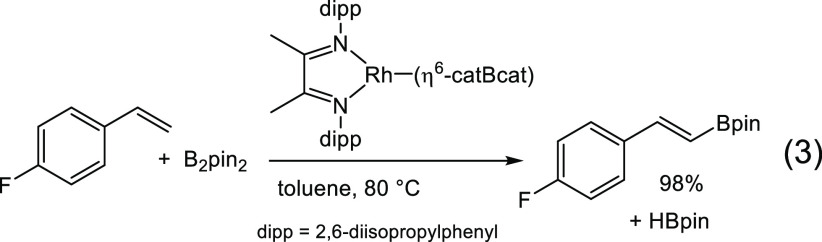
3

## Results and Discussion

### Synthesis, Reactivity, and NMR Characterization of the Iridium Complexes

In order to obtain an iridium(I) precursor with a labile ethylene ligand, [IrCl(COE)_2_]_2_ was dissolved in tetrahydrofuran (THF) and treated with excess ethylene at 77 K (eq 4). Upon thawing, the solution turned colorless indicating the transformation into IrCl(C_2_H_4_)_4_, which converts to the dinuclear compound [IrCl(C_2_H_4_)_2_]_2_ at RT.^14^ The addition of the α-diimine ligand (α-diimine = 1,4-bis(2,6-xylyl)-2,3-dimethyl-1,4-diaza-1,3-butadiene) generated IrCl(C_2_H_4_)(α-diimine), **1**. Complex **1** is a highly colored purple complex that is air sensitive. It is stable in the solid state and in THF solution at room temperature for long periods of time. It is insoluble in pentane and stable under vacuum but readily decomposes in refluxing pentane at 36 °C.^15^
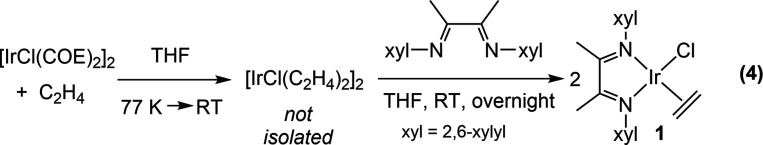
4

**Figure 1:**
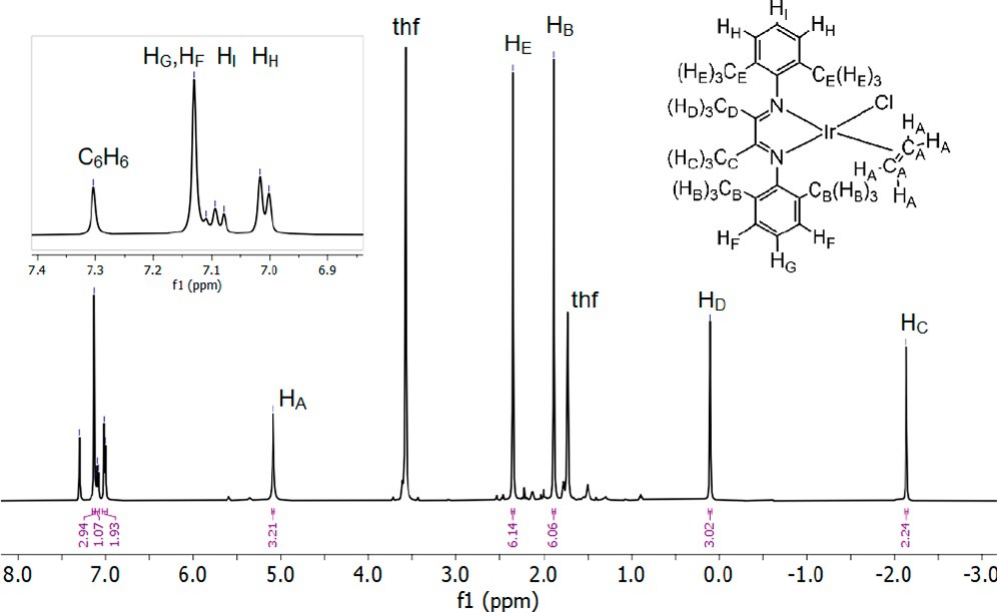
^1^H NMR spectrum (in THF-*d*_8_) and assignments for **1** based on ^1^H–^1^H NOESY and ^1^H–^13^C HSQC experiments. Assignments for **2**–**4** were made by comparison to **1**. **1**–**3**, X = Cl, Me, I; **4**, X = Cl, w/COE in place of C_2_H_4_.

Complex **1** has some noteworthy ^1^H NMR spectral properties (Figure 1). A NOESY spectrum was used to identify a chain of proximity from the ethylene protons all the way to H_E_ (H_A_–H_B_–H_C_–H_D_–H_E_). NOE interactions were also seen between H_B_/H_F_ and H_E_/H_H_. The hydrogens of the xylyl methyl groups of the coordinated α-diimine ligand appear at δ 2.35 and 1.88 (H_E_ and H_B_, respectively) which is in the typical region for benzylic hydrogens. The backbone methyl hydrogens H_C_ and H_D_ (α to the imine), however, are shifted significantly upfield. H_D_ has a chemical shift of δ 0.10, and H_C_ has a chemical shift of δ −2.14, which is unusual for a diamagnetic system. These shifts can be compared with the analogous shifts in the free ligand (δ 2.00), FeCl_2_(^3,5MePh^DAB^Me^) (δ 1.16), and ZnCl_2_(^3,5MePh^DAB^Me^) (δ 2.04).^16^ Furthermore, the ethylene ligand appears quite downfield for being coordinated to a transition metal at δ 5.09, which is not very different from that of free ethylene.^17^ These observations imply that there is not much π-backbonding to the ethylene and that the chlorido ligand is acting as a much better σ-donor than ethylene. However, the ^13^C resonance of the coordinated ethylene is shifted upfield to δ 50.21 (vs δ 123.09 for free ethylene), suggestive of significant backbonding. Furthermore, it has been noted that these chemical shifts can be very dependent on magnetic anisotropies in the complex, which might account for the variations seen here.^18^ The backbone methyl hydrogens H_C_ trans to the chlorido ligand are more upfield shifted than the backbone methyl hydrogens H_D_ trans to the ethylene ligand, which could be a result of this trans-influence. These unique chemical shifts show that both H_C_ and H_D_ of the coordinated ligand experience considerably more electron density than the free ligand itself. Selected ^1^H NMR data are summarized in Table 1.

**Table 1:** ^1^H NMR Data for Compounds **1**–**7** (THF-*d*_8_, 22 °C)

	xylyl	backbone Me	olefin, other
**1**	2.35 (s)	0.10 (s)	5.09 (s)
	1.88 (s)	–2.14 (s)	
**2**	2.43 (s)	–1.13 (s)	6.09 (s, CH_2_=CH_2_)
	1.66 (s)	–2.71 (s)	5.89 (Ir–CH_3_)
**3**	2.36 (s)	–0.19 (s)	5.21 (s)
	1.89 (s)	–2.47 (s)	
**4**	2.33 (s)	0.06 (s)	5.72 (d)
	1.90 (br s)	–2.24 (s)	
**5**	2.43 (s)	0.70 (s)	5.13 (s)
	1.91 (s)	–1.65 (s)	
**6**	2.18 (s)	0.00 (s)	
**7**^a^	2.32 (s)	1.66 (s)	3.05 (br s)
	2.11 (s)	0.62 (s)	

a –80 °C.

Attempts to alkylate the complex using common metathesis alkylating agents proved difficult. Reaction of **1** with MeLi, MeMgCl, or ZnMe_2_ produced the same methylated major product Ir(Me)(C_2_H_4_)(α-diimine), **2**, with different degrees of side reactions (eq 5, see NMR spectra in the Supporting Information). The backbone methyl groups of **2** are shifted quite upfield (δ −1.13 and −2.71), and the coordinated ethylene is downfield (δ 6.09), as in the case of **1**, along with a downfield peak attributed to the methyl ligand (δ 5.89). Burger and Nückel mentioned difficulty when attempting to isolate an iridium–pyridinediimine complex, Ir(*N*-(2,6-xylyl)-*N*-((1*E*)-1-{6-[(1*E*)-*N*-(2,6-dimethylphenyl)-ethanimidoyl]pyridine-2-yl}ethylidene)amine))Me.^19^ They noted the general sensitivity of the complex and were only able to isolate very small quantities of aluminum-free material via crystallization. Similar to **2**, Burger’s complex also has an Ir–Me resonance that is quite downfield (^1^H NMR, THF-*d*_8_, δ 6.91).^19^ Interestingly, there was a follow-up publication noting the stoichiometric C–H activation of benzene using this Ir–Me complex under mild conditions.^20^
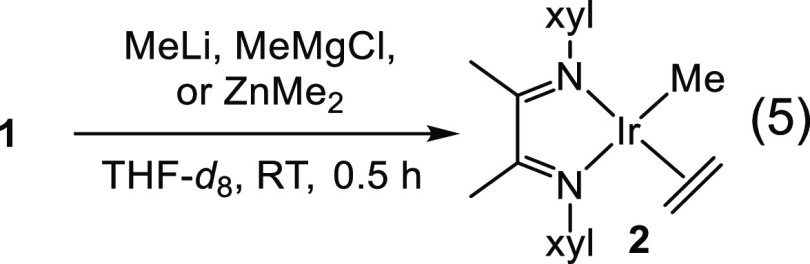
5

The use of metathesis reagents to synthesize a stable, isolable complex **2** proved ineffective, so our next attempt was to use the oxidative addition reagent iodomethane. Instead of forming the desired oxidative addition product Ir^III^Cl(Me)I(C_2_H_4_)(α-diimine), we instead observed halide exchange forming IrI(C_2_H_4_)(α-diimine), **3**, and chloromethane (eq 6).^21^ Complex **3** was confirmed by independent synthesis from the reaction of **1** with KI. Interestingly, **1** and **3** have almost identical resonances for H_B_ and H_E_, but H_C_ and H_D_ are even more upfield for **3** than **1**. A possible explanation for this is that because iodide is more polarizable than chloride, the α-diimine ligand is more able to pull electron density from iodide than chloride, thus adding electron density to the backbone methyl groups H_C_ and H_D_.
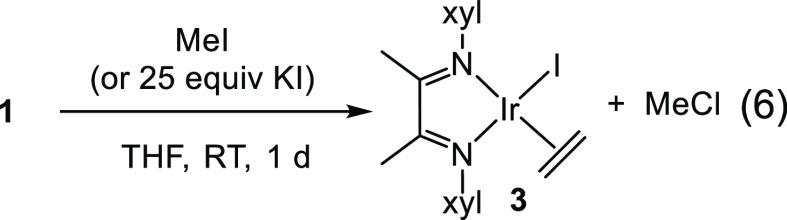
6

Repeating the general procedural parameters for the synthesis of **1** without the addition of ethylene generated the analogous complex IrCl(COE)(α-diimine), **4** (eq 7). The ^1^H NMR chemical shift for the olefinic hydrogen atoms (H_A_) was observed at δ 5.72 and methyl groups H_D_ and H_C_ at δ 0.06 and −2.24, respectively. Braun’s complex, using the same exact α-diimine ligand as we used in this report for the complex IrCl(^t^BuNC)(α-diimine), has methyl resonances from the α-diimine ligand at δ −0.09 and −2.41.^22^
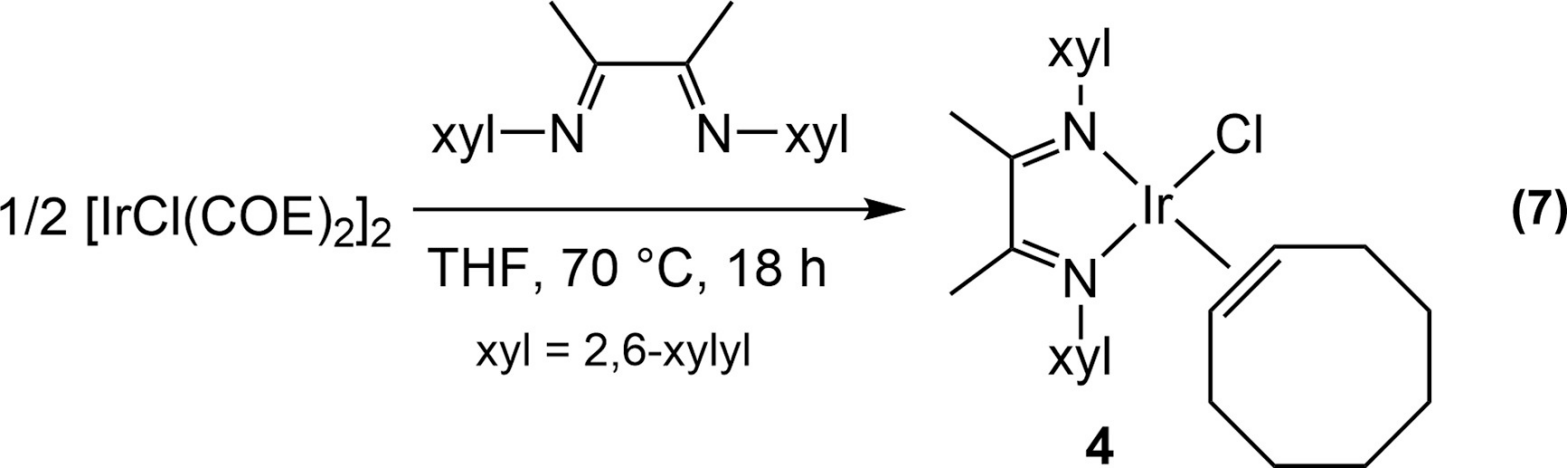
7

The treatment of complex **3** with 1 equiv of AgTFA (TFA = trifluoroacetate) gives a new product assigned as Ir(O_2_CCF_3_)(α-diimine)(C_2_H_4_), **5** (see the Experimental Section). Complex **5** was isolated as a sticky red-purple solid that could not be crystallized. The addition of benzene to a THF-*d*_8_ solution of **5** did not show any evidence for reaction with benzene at room temperature.

### UV–Vis Spectra of the Complexes

**Figure 2:**
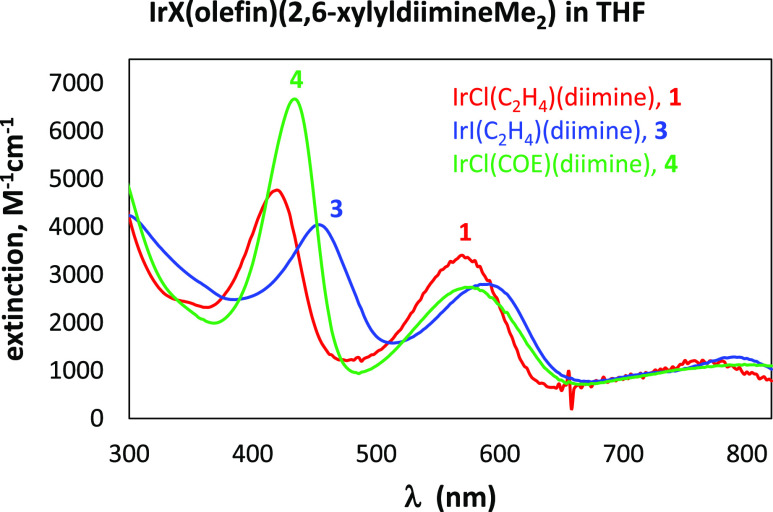
UV–vis spectra of iridium compounds **1**, **3**, and **4**.

A UV–vis spectrum was recorded for **1**, **3**, and **4**, as shown in Figure 2. Each displays three absorption bands in the visible region (Table 2). The high extinction suggests that these are MLCT bands (M → diimine-π*). It is possible that the high extinction coefficient and the large upfield shift for the backbone methyl groups may be caused by a low-lying singlet diradical MLCT excited state, as observed for other late transition metal complexes using α-diimine-type supporting ligands.^23−25^

**Table 2:** UV–Vis Data for Complexes **1**, **3**, and **4** in THF

compound	λ_1_, nm (ε, M^–1^ cm^–1^)	λ_2_, nm (ε, M^–1^ cm^–1^)	λ_3_, nm (ε, M^–1^ cm^–1^)
**1**	420 (4771)	570 (3407)	764 (1220)
**3**	454 (4046)	592 (2808)	790 (1286)
**4**	434 (6674)	574 (2738)	800 (1118)

### X-ray Crystallographic Characterization of **1** and **4**

**Figure 3:**
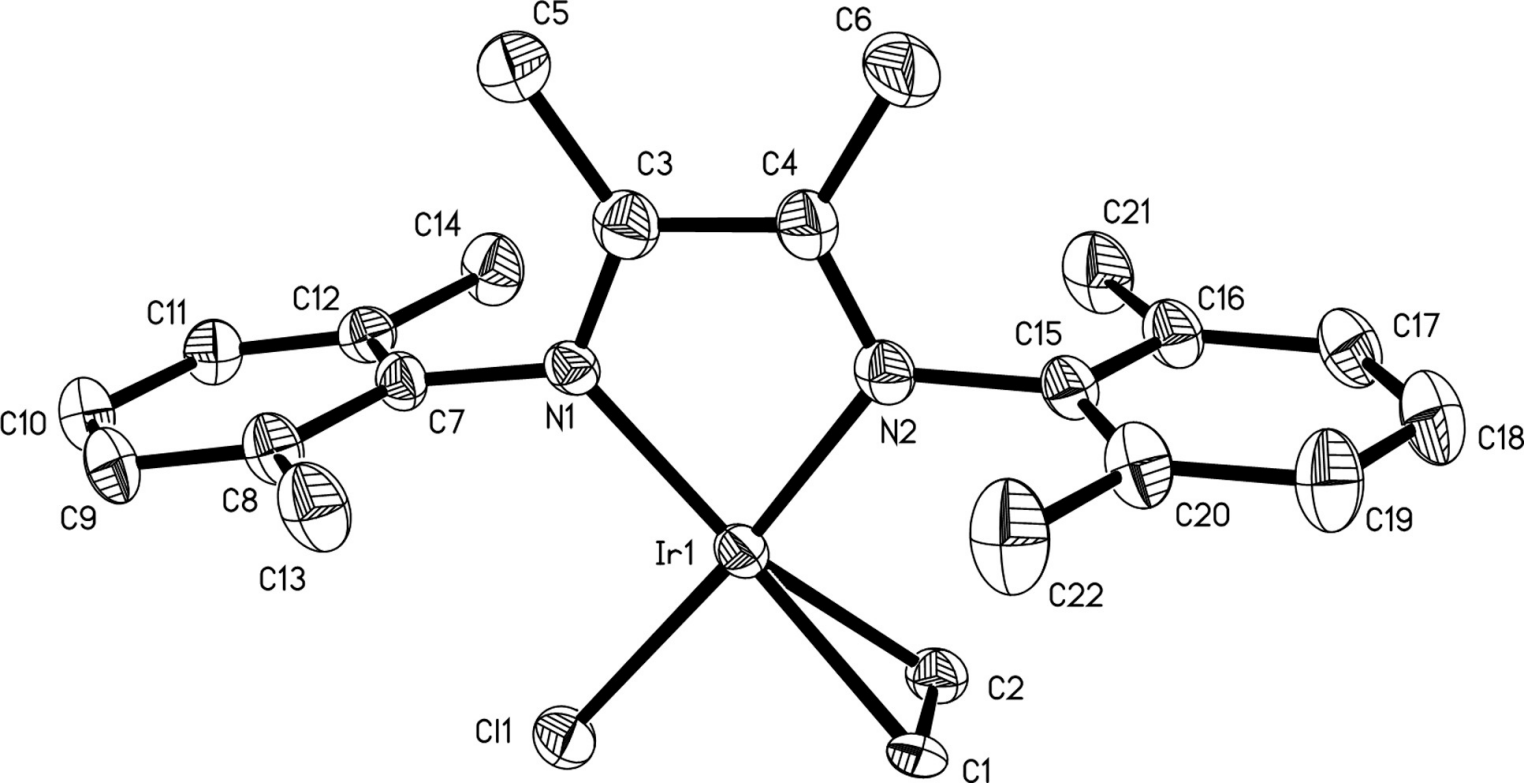
Molecular structure of **1** with hydrogen atoms omitted. Thermal ellipsoids are drawn at the 50% probability level. The chlorido and ethylene ligands are disordered.

**Figure 4:**
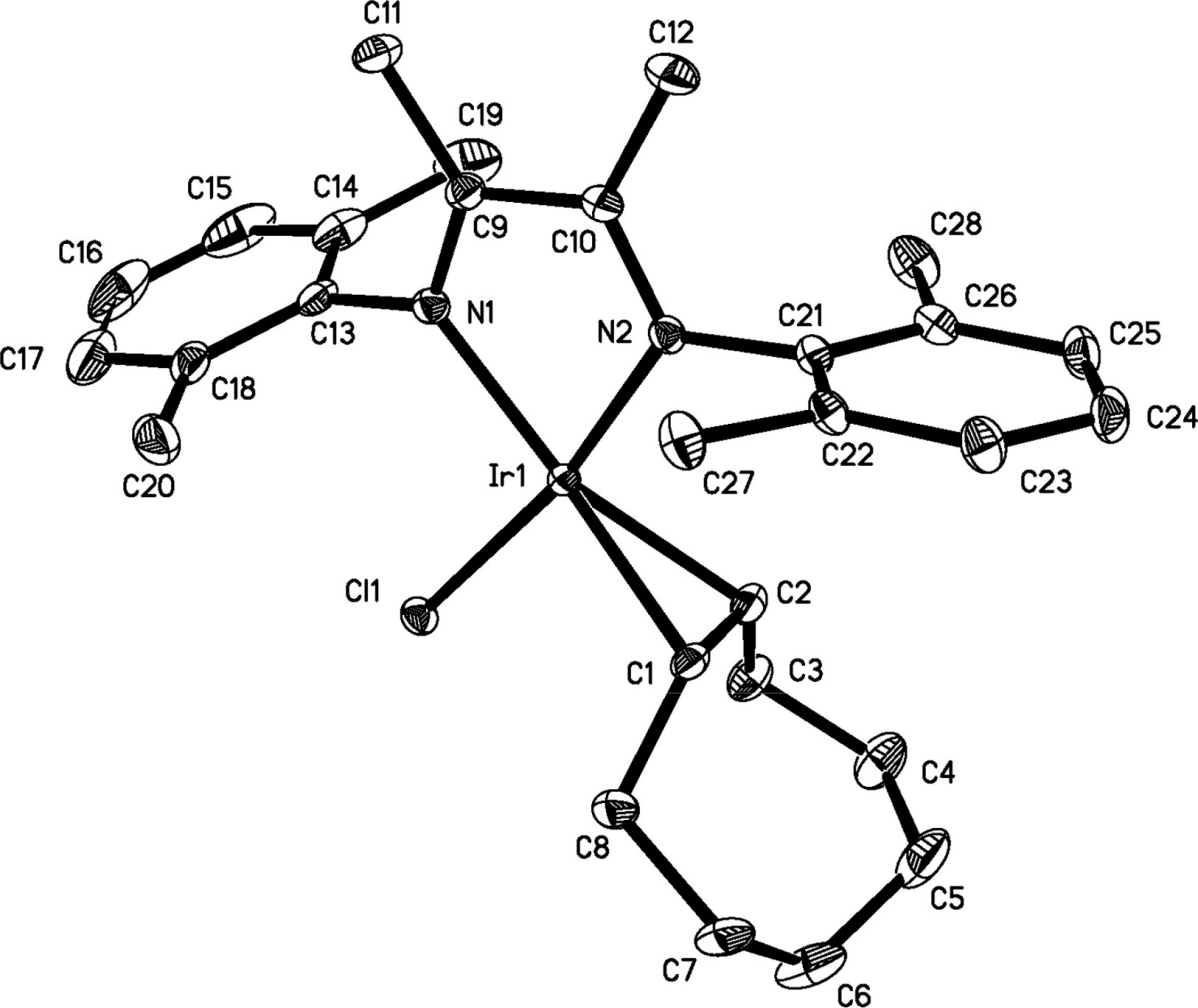
Molecular structure of **4** with hydrogen atoms omitted. Thermal ellipsoids are drawn at the 50% probability level.

Single crystals suitable for structure determination were grown for complexes **1** and **4**. **1** crystallizes with nearly a 1:1 disorder about the chlorido and ethylene ligands (Figure 3). **4** crystallizes without ligand disorder (Figure 4). The N=C double bonds for both **1** and **4** are lengthened by ∼0.05 Å and the diimine backbone C–C bonds are shortened by ∼0.06 Å when compared to the free ligand.^26^ These changes are consistent with the established redox noninnocence of α-diimine ligands^27−29^ and reflects the presence of some Ir^III^–metalladiazacyclopentene or Ir^II^–diimine π-radical anion character. In both complexes, the olefin is perpendicular to the square plane. Braun published the structure of RhCl(COE)(4,4′-di-*tert*-butyl-2,2′-bipyridine) which showed a similar arrangement of the COE ligand compared to **4**.^30^ The olefinic hydrogen atoms for Braun’s rhodium complex point away from the chlorido ligand, and the olefinic C–C bonds lengths^31^ (1.4005(5) Å and 1.386(5) Å) were comparable to that in **4** (1.402(4) Å). Unsurprisingly, the structure of IrCl(^t^BuNC)(α-diimine) is nearly identical with that of **1** and **4**, with replacement of the isocyanide ligand by an olefin. The metrics for several other diaryl−α-dimine complexes are provided in Table 3 for comparison. Note that complexes **1** and **4** have longer C=N bonds and shorter backbone C–C bonds than most other iridium (and rhodium) compounds, with the zinc(II) compounds representing molecules with no noninnocent behavior.^16^ Braun’s ^t^BuNC complex^13^ is closest to the values seen in **1** and **4**.

**Table 3:** Metrics for Several Diaryl−α-Dimine Complexes

compound^a^	*d*(C=N)	backbone *d*(C–C)	CCDC#	REFCODE
^2,4,6Me3Ph^DAB^Me^^32^	1.275 (inversion)	1.504	170457	NEMZAG
Ir^I^Cl(^2,6Me2Ph^DAB^Me^)(C_2_H_4_), **1**^b^	1.327(8), 1.336(8)	1.439(9)	2130719	b
Ir^I^Cl(^2,6Me2Ph^DAB^Me^)(COE), **4**^b^	1.317(3), 1.336(3)	1.438(3)	2130720	b
Ir^I^Cl(^2,6Me2Ph^DAB^Me^)(^t^BuNC)^13^	1.325(3), 1.311(4)	1.447(4)	811454	ITOHOP
[Ir^III^(^4FPh^DAB^Me^)ClCp*]^+^^33^	1.291(5), 1.299(5)	1.476(6)	1832696	ZIWCAM
[Ir^III^(^2,4,6Me3Ph^DAB^H^)Cp*Cl^11^	1.299(3), 1.297(3)	1.450(3)	876335	QIBWUV
Zn^II^(^Ph^DAB^Me^)Cl_2_^16^	1.286(2), 1.283(2)	1.520(3)	2054951	UTEQOC
Zn^II^(^3,5Me2Ph^DAB^Me^)Cl_2_^16^	1.282(2) (mirror)	1.522(2)	2054953	UTERAP
Zn^II^(^2,4,6Me3Ph^DAB^Me^)Cl_2_^16^	1.276(3), 1.279(3)	1.527(3)	2054958	UTERUJ
Fe^II^(^2,4,6Me3Ph^DAB^Me^)Cl_2_^16^	1.278(2), 1.280(2)	1.514(2)	2054960	UTESEU
^2,4,6Me3Ph^DAB^Me^^34^	1.278(2), (twofold)	1.500(2)	287384	ODAKUA
Fe^II^(^3,5Cl2Ph^DAB^Me^)Cl_2_(THF)_2_^16^	1.279(4), 1.279(4)	1.524(5)	2054955	UTESIY
^3,5Cl2Ph^DAB^Me^^16^	1.276(2) (inversion)	1.505(2)	2054957	UTEROD
[Rh^I^(^2,6Me2Ph^DAB^Me^)Cl]_2_, **6**^b^	1.323(2), 1.323(2)	1.437(2)	2130720	b
[Rh^I^(^2,6iPrPh^DAB^Me^)Cl]_2_^12^	1.325(3), 1.316(3)	1.426(3)	602790	WEKCUL
[Rh^I^(^3,5Me2Ph^DAB^Me^)(CO)_2_]^+^^35^	1.278(4), 1.284(4)	1.510(4)	799914	UMAVOU
[Rh^I^(^3,4,5MeO3Ph^DAB^Me^)(CO)_2_]^+^^35^	1.286(3) (mirror)	1.516(4)	799915	UMAVUA
[Rh^I^(^4ClPh^DAB^Me^)(CO)_2_]^+^^35^	1.280(5) (mirror)	1.508(8)	799917	UMAWEL
[Rh^III^(^Ph^DAB^Me^)(nbd)(PPh_3_)]^+^^36^	1.323, 1.282	1.48(1)	136305	WIWJUH
Rh^III^(^3,5Me2Ph^DAB^Me^)(CO)I_2_Me^37^	1.289(4), 1.285(3)	1.489(4)	1038899	TUMLEU
Rh^III^(^2iPrPh^DAB^Me^)(CO)I_2_Me^38^	1.299, 1.268	1.475	207866	HUYPIA
Rh^III^(^2,6iPr2Ph^DAB^Me^)(C(O)Me)I_2_^38^	1.296 (mirror)	1.487	207867	HUYPOG
[Rh^III^(^3,5Me2Ph^DAB^Me^)(H_2_O)_3_(C(O)Me)]^2+^^37^	1.293(2), 1.287(2)	1.485(2)	1038900	TUMLIY
[Rh^III^(^3,5Me2Ph^DAB^Me^)(H_2_O)_3_Me]^2+^^37^	1.286(4), 1.295(4)	1.486(4)	1038901	TUMLOE
[Rh^III^(^3,5Me2Ph^DAB^Me^)(H_2_O)(C(O)Me)(TFA)_2_]^2+^^37^	1.291(2), 1.293(2)	1.486(3)	1038903	TUMMAR
[Rh^III^(^4COOHPh^DAB^Me^)ClCp*]^+^^10^	1.289(4), 1.288(4)	1.491(4)	1923080	FOSYAQ

a Abbreviations: ^R1^DAB^R2^ represents a 2,3-R^2^-diazabutadiene with R^1^ groups attached to the nitrogen atoms.

b This work.

### Synthesis and Characterization of Rhodium Analogues

The synthesis of the related rhodium complex of **1** was attempted by the reaction of the bis-xylyl-α-diimine with [RhCl(C_2_H_4_)_2_]_2_, giving a dark purple solution. Examination of the reaction by ^1^H NMR spectroscopy, however, showed a ∼2:1 mixture of two products **6** and **7**, each with a diimine ligand. This ratio was seen to vary depending on the reaction conditions. In one reaction where the solution was stirred continuously under N_2_, **7** was the dominant product, and the solution was dark green.

In another reaction, the purple solution was subjected to a pressure of ethylene (2 atm), resulting in an immediate color change to green. The ^1^H NMR spectrum of the solution showed only resonances for **7**. It was hypothesized that the desired ethylene complex RhCl(C_2_H_4_)(α-diimine), **7**, was in equilibrium with the dimer [RhCl(α-diimine)]_2_, **6**, lacking the ethylene ligand (eq 8).
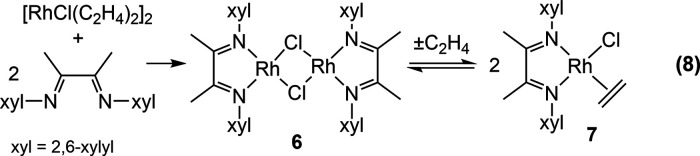
8

**Figure 5:**
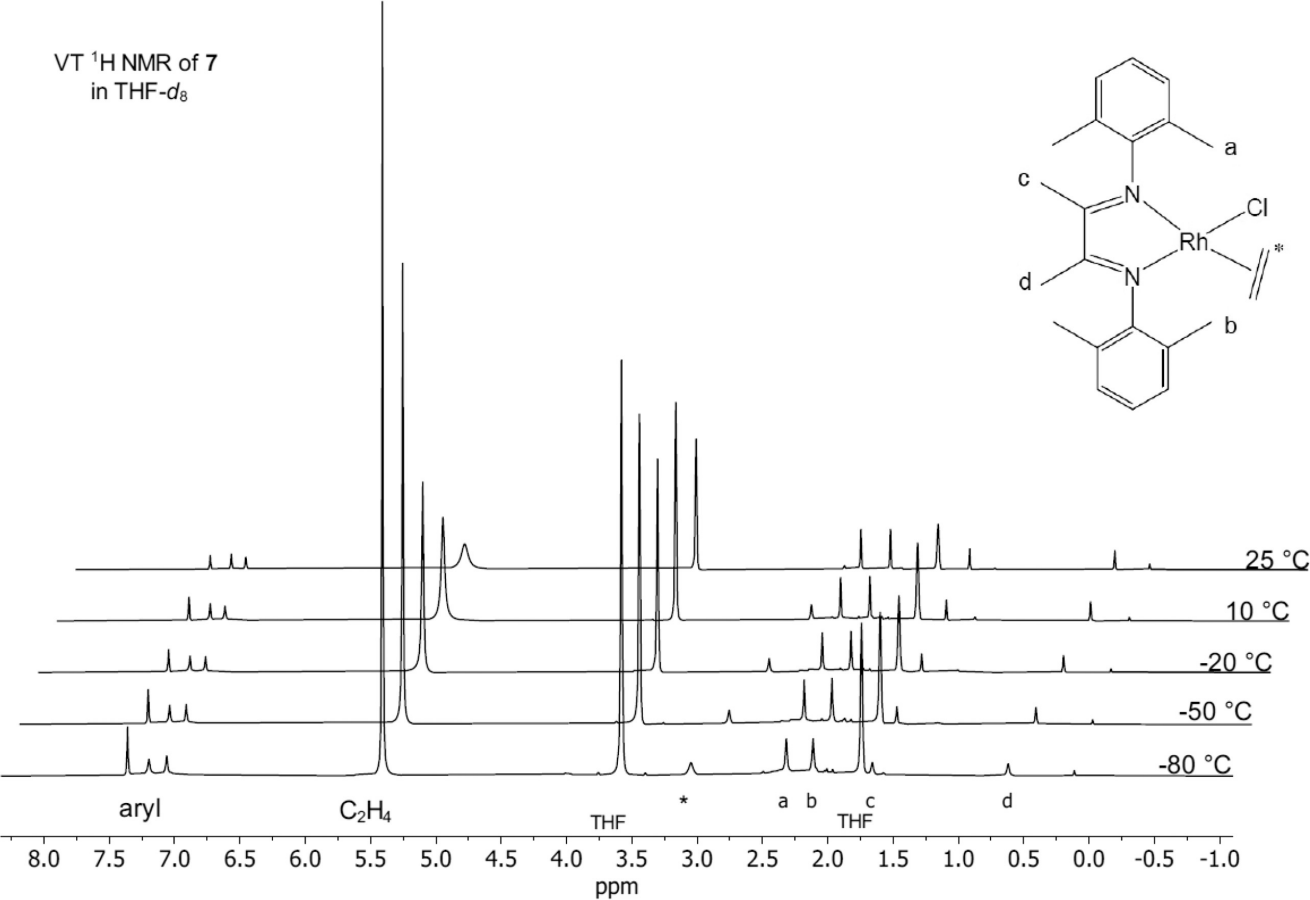
Variable temperature ^1^H NMR spectra of **7** in THF-*d*_8_ under 2 atm C_2_H_4_ (400 MHz).

The resonance for the coordinated ethylene in **7** could not be observed at room temperature. A ^13^C{^1^H} NMR spectrum showed no sharp resonances. Variable temperature NMR spectroscopy was used to observe the coordinated ethylene (Figure 5). A new resonance was seen to grow in at δ 3.05 below 10 °C, which can be assigned to the coordinated ethylene. The resonance for free ethylene appears at δ 5.4 as a broad peak, which sharpens as the temperature is lowered. The coordinated ethylene resonance broadens at −80 °C, suggesting that rotation is beginning to slow. Note that in the static structure, the ethylene hydrogens are inequivalent. The observation of a single broad resonance may be due to near isochronous shifts combined with rapid rotation. From the line width at −80 °C (14.8 Hz), a rotation rate of 82 s^–1^ can be estimated.^39^

**Figure 6:**
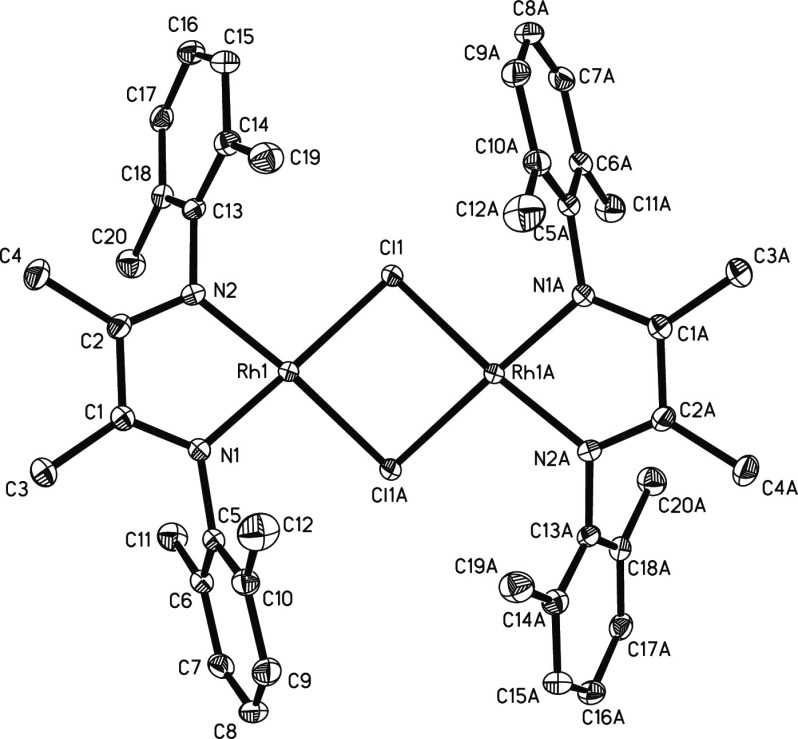
Diagram of **6** with thermal ellipsoids drawn at the 50% probability level. Hydrogen atoms and cocrystallized CH_2_Cl_2_ molecules were omitted for clarity. *d*(Rh1–Rh1A) = 3.290 Å.

The complete removal of ethylene gas under vacuum produced mainly **6**, but some **7** was still present. Consequently, **6** was independently prepared by the reaction of [RhCl(COE)_2_]_2_ with the diimine (eq 9). The red product was obtained cleanly after washing to remove COE. Compound **6** could be recrystallized from dichloromethane/pentane to give X-ray quality crystals. The structure of **6** in Figure 6 shows a view down the crystallographic twofold axis and confirms that this compound has lost the coordinated ethylene and forms a bis-μ-chlorido dimer. Each RhCl_2_N_2_ moiety is square planar with the Rh_2_Cl_2_ unit bent along the Cl–Cl axis at 137.7°. The fact that pure **6** is red whereas **7** is green also explains why the mixture of **6** and **7** is purple.
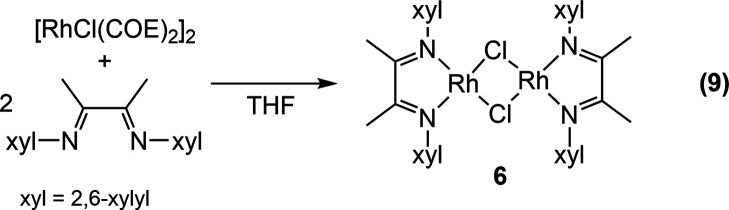
9

Compound **6** also displays a ^1^H NMR resonance at δ 0.0 for the two diimine backbone methyl groups, which is similar to one of the two analogous resonances in **1** and **4**. While it is tempting to say this shift can be associated with a methyl group trans to the chlorido ligand, the differing nature of μ_1_-Cl vs μ_2_-Cl makes assignments on the basis of trans-ligand effects unreliable.

## Conclusions

Here we show the synthesis of nonpyridine based olefin complexes of iridium(I) using an α-diimine supporting ligand. Complex **1** was not easily alkylated, but the work by Braun showed that this same diimine supporting ligand when coordinated to iridium(I) using an isocyanide ligand (not ethylene as in the present case) displayed interesting peroxo and dihydrogen chemistry. Thus, selection of the correct neutral, monodentate coligand in this system is critical to reactivity. No reactions with the C–H bonds of benzene were observed, in contrast to the examples shown in eqs 1–2.

## Experimental Section

### General Considerations

All reactions were performed under N_2_ using standard glovebox and/or Schlenk techniques. Pentane and THF were dried and deoxygenated by passage through activated alumina and Q5 (oxygen scavenger) columns from Glass Contour Co. (Laguna Beach, CA) or were distilled from Na/benzophenone ketyl. THF-*d*_8_ (Cambridge) was distilled from Na/benzophenone ketyl. Iodomethane was dried over CaSO_4_ and vacuum distilled. MeMgCl (2.6 M in THF, Aldrich) was titrated against 2-butanol in toluene using 1,10-phenanthroline as an indicator prior to use. KI (J. T. Baker) was used as received. [IrCl(COE)_2_]_2_ was synthesized according to the literature.^40^ Elemental analyses were determined at the CENTC Elemental Analysis Facility at the University of Rochester using a PerkinElmer 2400 Series II analyzer equipped with a PerkinElmer Model AD-6 autobalance by Dr. William W. Brennessel. NMR spectra were collected on Bruker Avance NMR spectrometers operating at ^1^H NMR frequencies of 400 or 500 MHz and calibrated to residual solvent signals (THF-*d*_8_, 25 °C, δ 3.58, 1.73). ^1^H and ^13^C{^1^H} NMR spectral assignments for **1** were determined by comparison with ^1^H–^1^H NOESY and ^1^H–^13^C HSQC experiments. ^1^H NMR spectral assignments for **2** and **3** were determined by comparing to the assignments for **1**. ^1^H and ^13^C{^1^H} NMR spectral assignments for **4** were determined by comparing to results from **1**. UV–vis spectra were obtained on a Hewlett-Packard 8452A Diode Array Spectrophotometer.

### Synthesis of IrCl(C_2_H_4_)(ArN=C(Me)C(Me)=NAr) (Ar = 2,6-Me_2_C_6_H_3_), **1**

A 250 mL Schlenk flask was loaded with [IrCl(COE)_2_]_2_ (602 mg, 0.672 mmol) and THF (100 mL). The contents were cooled to 77 K, and ethylene (excess) was condensed into the flask. Upon thawing, ethylene pressure expanded the septum, and periodically this excess gas pressure was vented through a needle to prevent the vessel from rupturing. The solution turned from orange to colorless. An α-diimine (369 mg, 1.26 mmol) solution in THF (10 mL) was added, and the contents were vigorously stirred overnight at room temperature. The volatiles were removed under vacuum, and the dark solid was washed with pentane (500 mL, until the green colored filtrate became colorless) affording a dark purple solid (370 mg, 54%). Crystals suitable for structure determination were grown by pentane diffusion into a THF solution of **1** at −20 °C. Anal. Calcd for C_22_H_28_ClIrN_2_: C, 48.21; H, 5.15; N, 5.11. Found: C, 48.42; H, 5.30; N, 4.73. ^1^H NMR (500 MHz, THF-*d*_8_, 22 °C): δ 7.13 (s, 3H_F+G_), 7.09 (t, *J* = 7.5 Hz, 1H_I_), 7.01 (d, *J* = 7.5 Hz, 2H_H_), 5.09 (s, 4H_A_), 2.35 (s, 6H_E_), 1.88 (s, 6H_B_), 0.10 (s, 3H_D_), −2.14 (s, 3H_C_). ^13^C{^1^H}NMR (125 MHz, THF-*d*_8_, 25 °C): δ 188.04 (s, C), 173.96 (s, C), 153.99 (s, C), 149.55 (s, C), 128.89 (s, CH_ARYL_), 128.77 (s, C), 128.51 (s, C), 128.35 (s, CH_ARYL_), 127.68 (s, CH_ARYL_), 126.85 (s, CH_ARYL_), 50.21 (s, C_A_), 24.70 (s, C_C_), 20.27 (s, C_D_), 19.29 (s, C_E_), 17.24 (s, C_B_). HSQC and NOESY spectra were used to assign resonances (see the Supporting Information).

### Synthesis of Ir(Me)(C_2_H_4_)(α-diimine), **2**

A typical procedure begins by loading a J-Young NMR tube with **1** (5–10 mg) and THF-*d*_8_ (0.6 mL). Addition of 1 equiv of ZnMe_2_ (9.5% wt/wt in hexane), MeMgCl (2.6 M in THF), or MeLi (1.6 M in diethyl ether) at room temperature produced a dark green mixture after 0.5 h. Attempts to isolate the product were not successful. The ZnMe_2_ reactions were probably the cleanest while the MeLi and MeMgCl reactions produced more side products. The methyl product **2** decomposed during attempts to purify it. See the Supporting Information for ^1^H NMR spectra. ^1^H NMR (400 MHz, THF-*d*_8_, 22 °C): (major product) δ 7.15–6.94 (m, 6H, C*H*_ARYL_), 6.09 (s, 4H, *H*_A_), 5.89 (s, 3H, Ir–C*H*_3_), 2.43 (s, 6H, *H*_E_), 1.66 (s, 6H, *H*_B_), −1.13 (s, 3H, *H*_D_), −2.71 (s, 3H, *H*_C_).

### Synthesis of IrI(C_2_H_4_)(ArN=C(Me)C(Me)=NAr) (Ar = 2,6-Me_2_C_6_H_3_), **3**

A 20 mL scintillation vial was loaded with **1** (20.1 mg, 0.0367 mmol), KI (150.8 mg, 0.908 mmol), and THF (6 mL) and then set vigorously stirring at room temperature for 20 h. The volatiles were removed in vacuo. The crude mixture was taken up in benzene (a total of 24 mL) and filtered through a Celite plug to remove KCl and KI. The filtrate was collected, and the volatiles were removed in vacuo resulting in 23.5 mg (76%, crude) of a dark green solid. Anal. Calcd for C_22_H_28_IIrN_2_: C, 41.31; H, 4.41; N, 4.38. Found: C, 41.09; H, 4.32; N, 4.06. ^1^H NMR (400 MHz, C_6_D_6_, 22 °C): δ 7.14 (m, 1H), 7.03 (d, *J* = 7.5 Hz, 2H), 6.92 (dd, *J* = 8.1, 7.0 Hz, 1H), 6.81 (d, *J* = 7.6 Hz, 2H), 5.92 (s, 4H), 2.44 (s, 6H), 1.67 (s, 6H), −0.95 (s, 3H), −3.30 (s, 3H). ^1^H NMR (500 MHz, THF-*d*_8_, 22 °C) δ 7.17 (dd, *J* = 8.8, 5.5 Hz, 1H), 7.13 (d, *J* = 9.0 Hz, 2H), 7.08 (dd, *J* = 8.3, 6.5 Hz, 1H), 7.02 (d, *J* = 7.5 Hz, 2H), 5.21 (s, 4H), 2.36 (s, 6H), 1.89 (s, 6H), −0.19 (s, 3H), −2.47 (s, 3H). ^13^C{^1^H} NMR (126 MHz, THF-*d*_8_, 22 °C): δ 188.70 (s), 174.33 (s), 156.37 (s), 147.66 (s), 129.21 (s), 128.94 (s), 128.28 (s), 128.00 (s)0, 127.73 (s), 127.02 (s), 47.33 (s), 21.10 (s), 19.41 (s), 18.94 (s), 17.28 (s).

### Reaction of **1** with Iodomethane

A J-Young tube was loaded with **1** (8.8 mg, 0.016 mmol) and dissolved in THF-*d*_8_, and iodomethane (1 μL, 0.016 mmol) was added at room temperature. The vessel was placed on an inverting NMR tube mixing device for 22.5 h, resulting in a dark mixture. A ^1^H NMR spectrum showed the formation of a new product consistent with **3**, a small singlet at δ 2.99 consistent with chloromethane,^21^ and a significant amount of starting material **1**. After 3 months at room temperature, the ratio of **3**:**1** did not significantly change. A GCMS showed the formation of methyl chloride (*m*/*z* = 50/52).

### Synthesis of IrCl(COE)(ArN=C(Me)C(Me)=NAr) (Ar = 2,6-Me_2_C_6_H_3_), **4**

A 50 mL resealable flask was loaded with [IrCl(COE)_2_]_2_ (100.0 mg, 0.1116 mmol), α-diimine (65.0 mg, 0.222 mmol), and THF (10 mL), and the flask was sealed and heated at 70 °C for 18 h. The volatiles were removed in vacuo at 70 °C. In order to remove trace amounts of COE, benzene (6 mL) was added, the volatiles were removed in vacuo, and the solid residue was placed under vacuum overnight at 70 °C. Benzene (8 mL) was used to transfer the green solid to a preweighed vial, the volatiles were removed in vacuo, and the solid was placed under vacuum at 70 °C overnight producing 135.5 mg (97%) of a dark green solid. Crystals suitable for structure determination were grown from a pentane solution left at −20 °C for 2 years. Anal. Calcd for C_28_H_38_ClIrN_2_: C, 53.36; H, 6.08; N, 4.44. Found: C, 53.35; H, 6.05; N, 4.17. ^1^H NMR (500 MHz, THF-*d*_8_, 22 °C): δ 7.15 (s, 3H, CH_ARYL_), 7.09 (t, *J* = 7 Hz, 1H, CH_ARYL_), 6.98 (d, *J* = 7 Hz, 2H, CH_ARYL_), 5.72 (d, *J* = 9 Hz, 2H, H_A_), 2.33 (s, 6H, H_E_), 1.90 (br s, 6H + 2H, H_B_ + CH_2_), 1.47 (d, *J* = 9 Hz, 6H, CH_2_), 1.37–1.18 (m, 4H, CH_2_), 0.06 (s, 3H, H_D_), −2.24 (s, 3H, H_C_). ^13^C{^1^H} NMR (125 MHz, THF-*d*_8_, 22 °C): δ 186.47 (s, C), 172.80 (s, C), 154.98 (s, C), 149.27 (s, C), 129.33 (s, C), 128.79 (s, CH_ARYL_), 128.18 (s, C), 128.12 (s, CH_ARYL_), 127.62 (s, CH_ARYL_), 126.62 (s, CH_ARYL_), 70.44 (s, C_A_), 31.75 (s, CH_2_), 29.71 (s, CH_2_), 27.67 (s, CH_2_), 25.04 (s, C_C_), 20.16 (s, C_D_), 19.36 (s, C_E_), 17.43 (s, C_B_).

### Synthesis of Ir(OC(O)CF_3_)(C_2_H_4_)(ArN=C(Me)-C(Me)=NAr) (Ar = 2,6-Me_2_C_6_H_3_), **5**

Silver trifluoroacetate (2.7 mg, 0.012 mmol) and **3** (6.7 mg, 0.012 mmol) were dissolved in THF-*d*_8_ and stirred at room temperature for several minutes. The product solution was filtered through a short column (2 cm) of Celite and washed with THF, and the volatiles were removed under vacuum yielding a reddish purple sticky solid. The material resisted crystallization attempts. ^1^H NMR (400 MHz, THF-*d*_8_, 22 °C): δ 7.09 (m, 6H), 5.13 (s, 4H), 2.43 (s, 6H), 1.91 (s, 6H), 0.70 (s, 3H), −1.65 (s, 3H). Addition of 10 μL of C_6_H_6_ to the NMR sample at 22 °C showed no changes.

### Synthesis of [RhCl(ArN=C(Me)C(Me)=NAr)]_2_ (Ar = 2,6-Me_2_C_6_H_3_), **6**

2,6-Xylyldimethyl-α-diimine (188 mg, 0.643 mmol) was dissolved in 10 mL of THF and added dropwise to a solution of [RhCl(COE)_2_]_2_ (232 mg, 0.323 mmol) in 10 mL of THF. The solution was stirred at room temperature for 72 h. The volatiles were removed under vacuum at 35 °C overnight. A ^1^H NMR spectrum showed product **6** with traces of COE. The solid was washed with cold pentane (3 × 2 mL) and dried under vacuum to obtain a dark red solid. Yield, 96 mg (35%). Anal. Calcd for C_40_H_48_Cl_2_N_4_Rh_2_: C, 55.76; H, 5.62; N, 6.50. Found: C, 55.40; H, 5.54; N, 6.25. ^1^H NMR (400 MHz, THF-*d*_8_, 22 °C): 7.11 (t, *J* = 7.5 Hz, 2H), 7.05 (d, *J* = 7.5 Hz, 4H), 2.18 (s, 12H), 0.00 (s, 6H). ^13^C{^1^H} NMR (126 MHz, THF-*d*_8_, 22 °C) δ 157.81 (s), 153.89 (s), 130.55 (s), 128.46 (s), 126.07 (s), 19.18 (s), 18.04 (s). Crystals suitable for structure determination were grown by dissolving in CH_2_Cl_2_ and layering with pentane at −20 °C for 17 months.

### Synthesis of RhCl(C_2_H_4_)(ArN=C(Me)C(Me)=NAr), **7**

[RhCl(α-diimine)]_2_ (20 mg, 0.027 mmol) was dissolved in 1 mL of THF-*d*_8_ in a high-pressure medium-walled NMR tube. A pressure of C_2_H_4_ (2 atm) was introduced to give a green solution. ^1^H NMR (400 MHz, THF-*d*_8_, −80 °C): δ 7.20 (s, 4H), 7.06 (s, 2H), 3.05 (br s, 4H), 2.32 (s, 6H), 2.11 (s, 6H), 1.66 (s, 3H), 0.62 (s, 3H). ^13^C{^1^H} NMR (101 MHz, THF-*d*_8_, −80 °C): δ 168.84, 149.74, 129.92, 129.33, 129.20, 129.13, 128.79, 125.32, 124.03, 123.47, 65.40 (d, *J* = 22.2 Hz), 21.80, 19.25, 18.08, 15.92. The resonances for the bound C_2_H_4_ and the two backbone methyl groups shift upfield ∼0.5 ppm as the temperature rises to 25 °C.

## Supporting Information Available

^1^H and ^13^C{^1^H} NMR spectra of compounds; X-ray structural details and data for **1**, **3**, and **6**. (PDF)

## Dedication

Dedicated to Professor Maurice Brookhart on the occasion of his 80th birthday.
