# Supplementary material for: Iridium(I)– and Rhodium(I)–Olefin Complexes Containing an α-Diimine Supporting Ligand
Source: Organometallics. 2022 Apr 5;41(22):3167–74. doi: 10.1021/acs.organomet.2c00036 (PMC9710518; doi:10.1021/acs.organomet.2c00036)
Supplement: Supplementary file 1 — om2c00036_si_001.pdf [file om2c00036_si_001.pdf]

Supporting Information for

Iridium(I)- and Rhodium(I)-Olefin Complexes Containing an α-Diimine Supporting Ligand

James Kovach, Suzanne R. Golisz, William W. Brennessel, William D. Jones*

Department of Chemistry, University of Rochester, Rochester, NY 14627

Figure S-1. ^{1}H NMR spectrum of 2,6-xylyldiimine	S2
Figure S-2. ^{13}C{^{1}H} NMR spectrum of 2,6-xylyldiimine	S3
Figure S-3. ^{1}H NMR spectrum of **1**	S4
Figure S-4. ^{13}C{^{1}H} NMR spectrum of **1**	S5
Figure S-5. HSQC NMR spectrum of **1**	S6
Figure S-6. NOESY NMR spectrum of **1**	S7
Figure S-7. ^{1}H NMR spectrum of **2** (via 3 routes)	S8
Figure S-8. ^{1}H NMR spectrum of **3**	S9
Figure S-9. ^{13}C{^{1}H} NMR spectrum of **3**	S10
Figure S-10. ^{1}H NMR spectrum of **4**	S11
Figure S-11. ^{13}C{^{1}H} NMR spectrum of **4**	S12
Figure S-12. ^{1}H NMR spectrum of **5**	S13
Figure S-13. ^{1}H NMR spectrum of **6**	S14
Figure S-14. ^{13}C{^{1}H} NMR spectrum of **6**	S15
Figure S-15. ^{1}H NMR spectrum of **7**	S16
Figure S-16. ^{13}C{^{1}H} NMR spectrum of **7**	S17
Figure S-17. VT ^{1}H NMR spectrum of **7**	S18
Figure S-18. VT ^{1}H NMR spectrum of **7** (expanded and overlaid)	S19
Figure S-19. Equilibrium VT ^{1}H NMR spectrum of equilibrium between **6** & **7**	S20
X-ray data for **1**	S21
X-ray data for **4**	S33
X-ray data for **6**	S46

Compound Index:

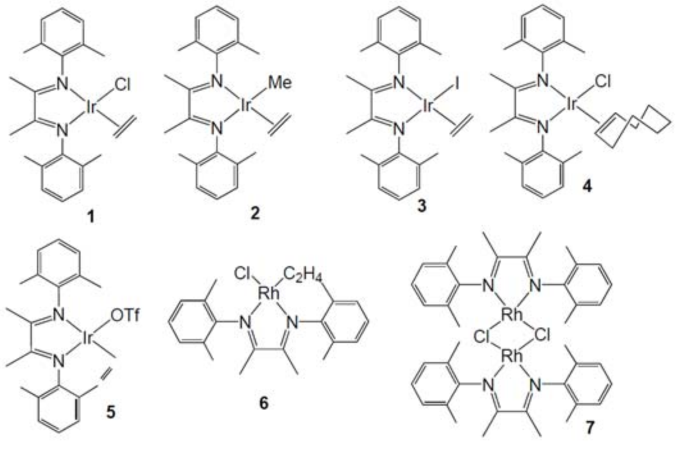

Figure S-1. ^{1}H NMR of 2,6-xylyldiimine in C_6D_6

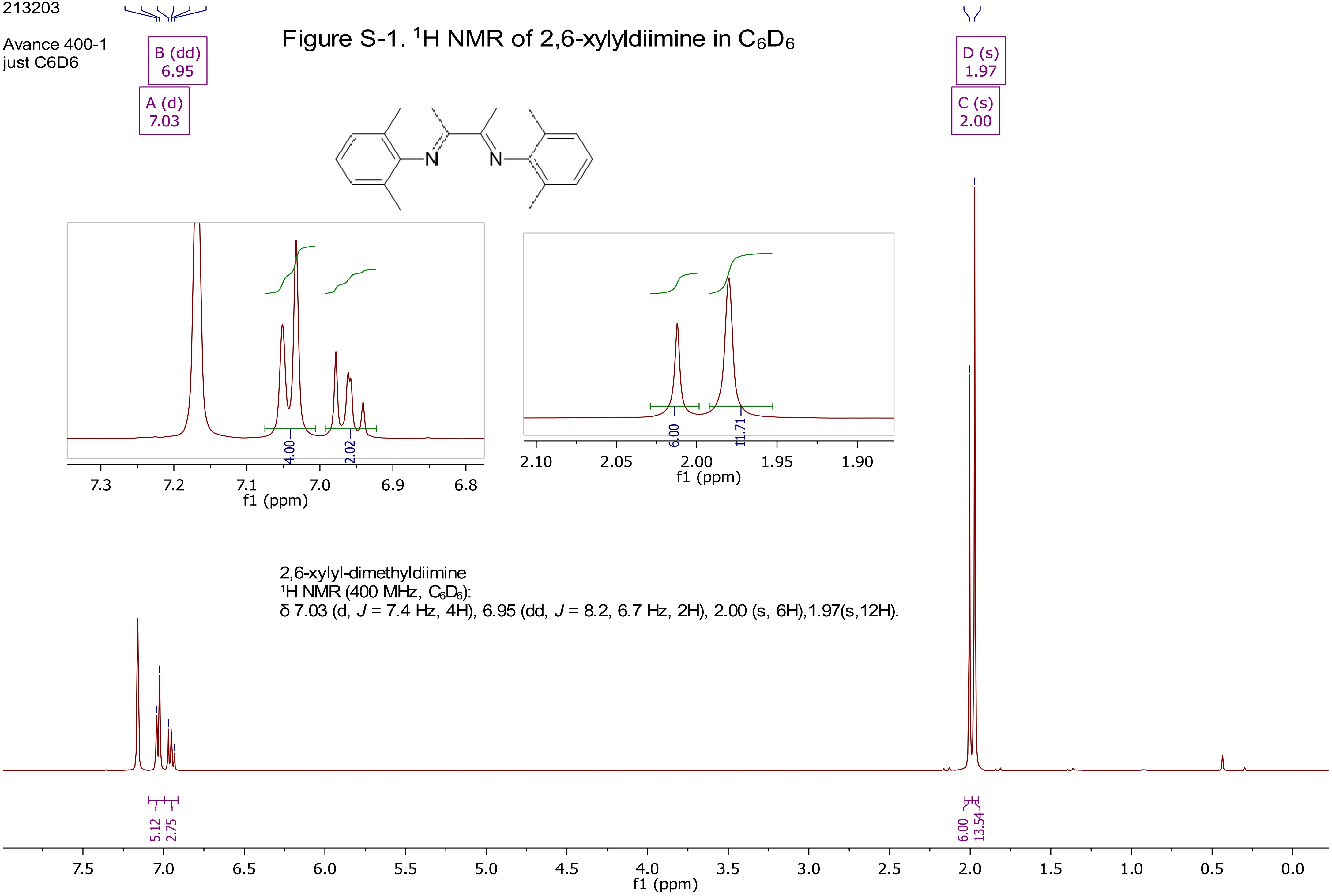

2,6-xylyl-dimethyldiimine
^{1}H NMR (400 MHz, C_6D_6):
δ 7.03 (d, J = 7.4 Hz, 4H), 6.95 (dd, J = 8.2, 6.7 Hz, 2H), 2.00 (s, 6H),1.97(s,12H).

$^{13}C\{^1H\}$ NMR (101 MHz, THF-d_8) δ 166.21, 147.12, 126.18, 122.69, 121.42, 15.47, 13.31.

Figure S-2. $^{13}C\{^1H]$ NMR of 2,6-xylyldiimine in C_6D_6

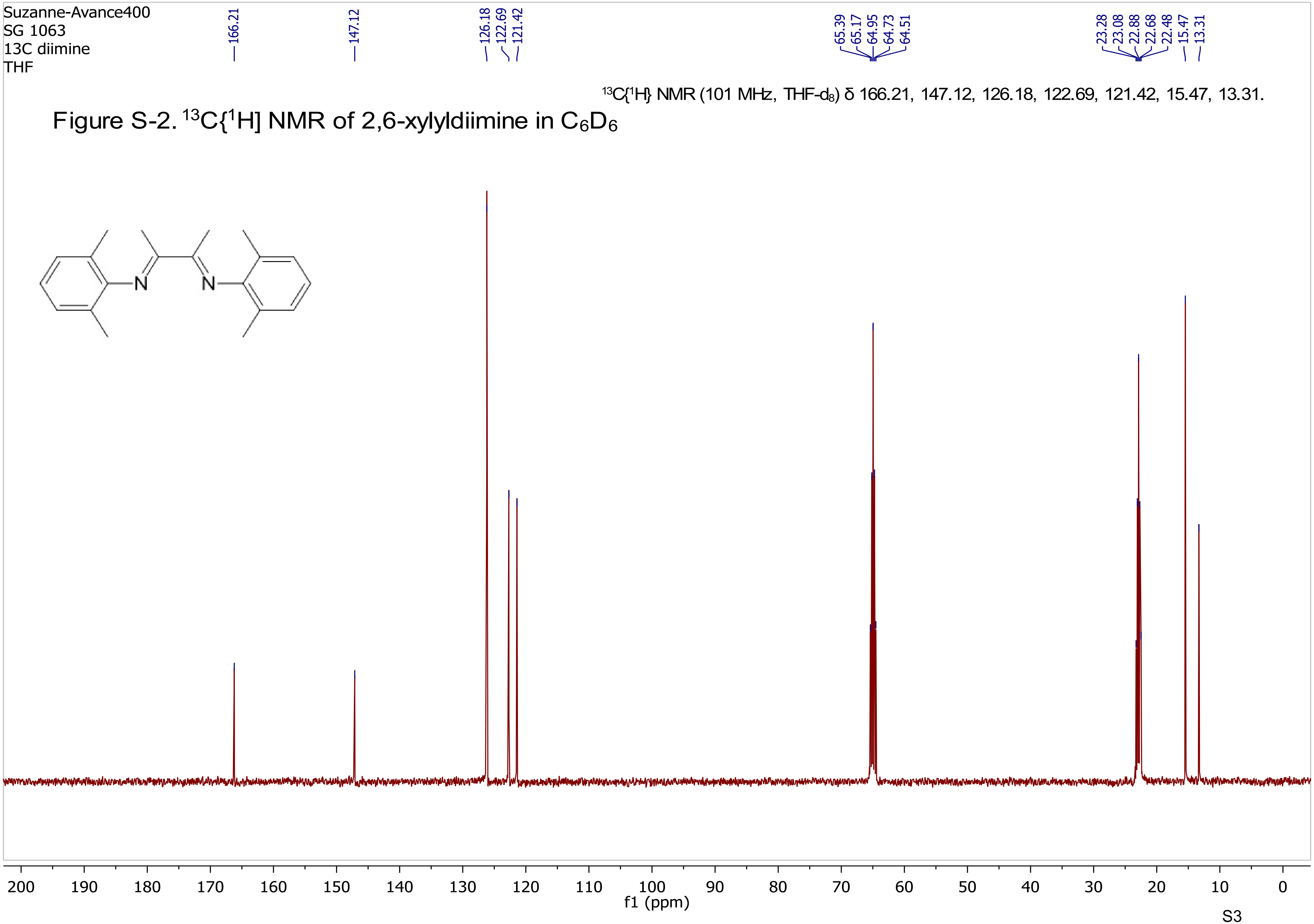

Figure S-3. ^1H NMR of **1** in THF-d_8

^1H NMR (500 MHz, THF-d_8) δ 7.13 (s, 3 H), 7.09 (t, J = 7.5 Hz, 1 H), 7.01 (d, J = 7.5 Hz, 2 H), 5.09 (s, 3 H), 2.35 (s, 6 H), 1.88 (s, 6 H), 0.10 (s, 3 H), -2.14 (s, 3 H).

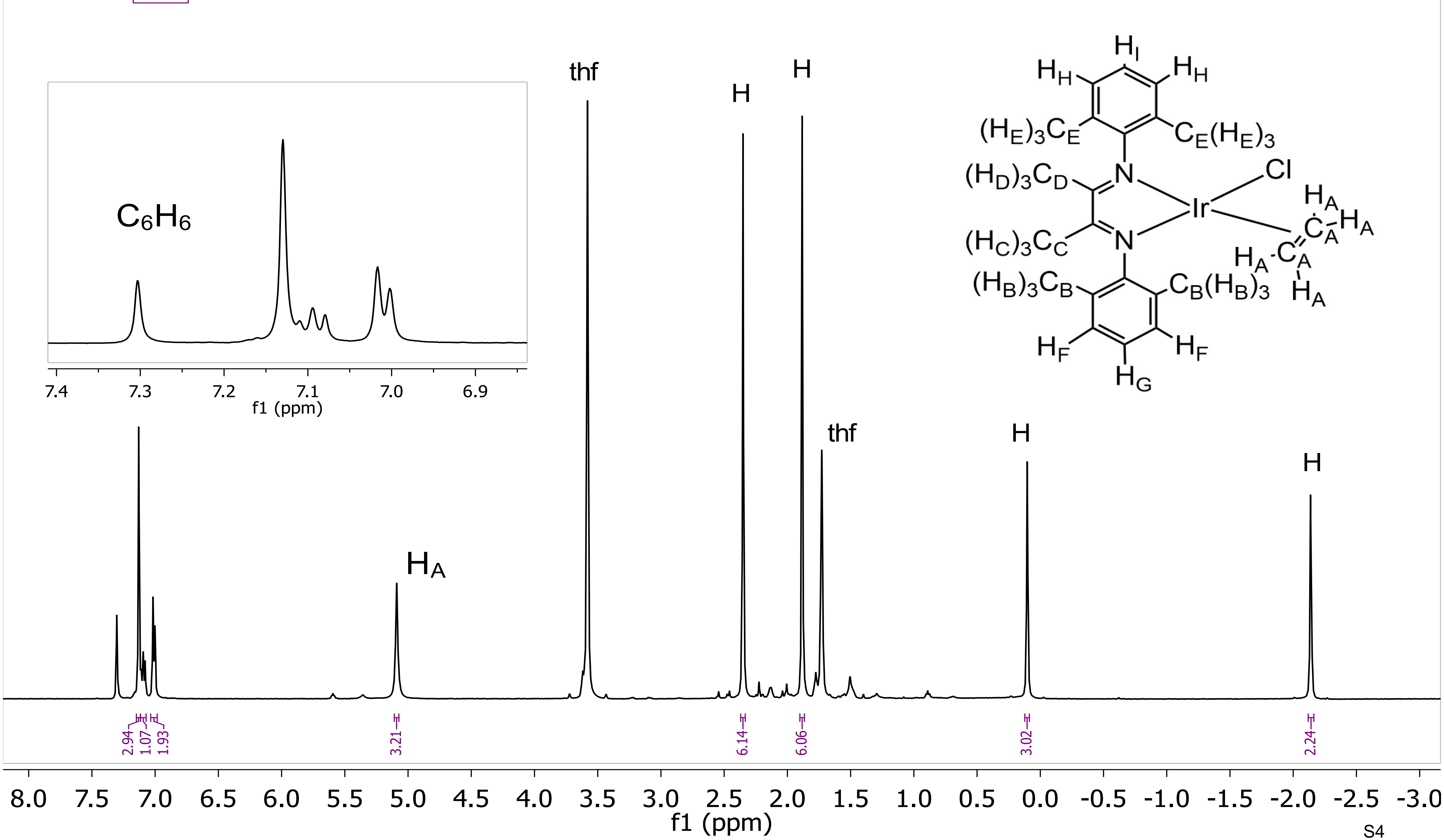

Figure S-4. $^{13}C\{^{1}H\}$ NMR of **1** in THF-d_8

^{13}C NMR (126 MHz, THF-d_8) δ 188.04, 173.96, 153.99, 149.55, 128.89, 128.77, 128.51, 128.35, 127.68, 126.85, 67.57, 50.21, 25.49, 24.70, 20.27, 19.29, 17.24.

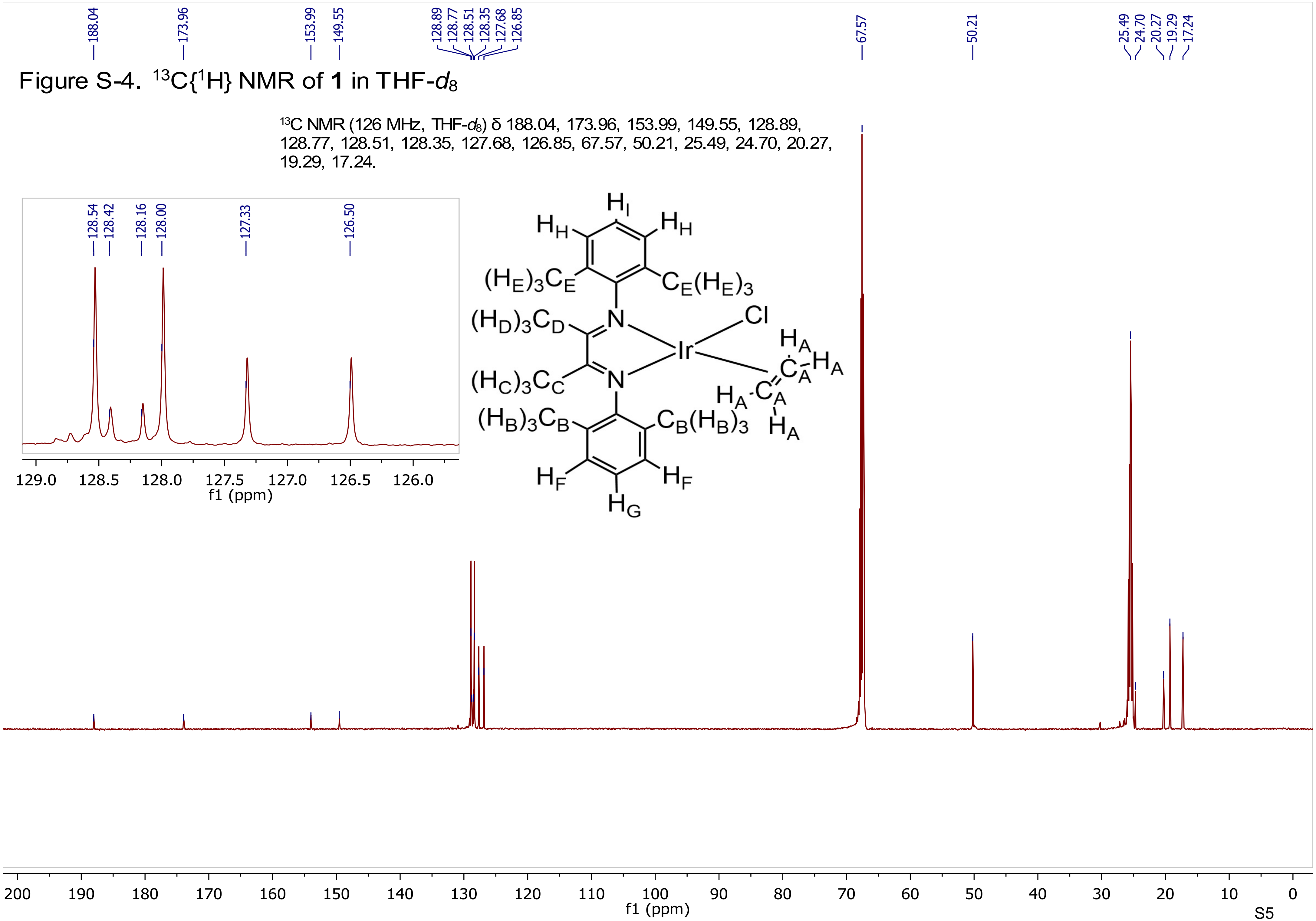

Figure S-5. HSQC of **1** in THF-d_8 with z-gradient

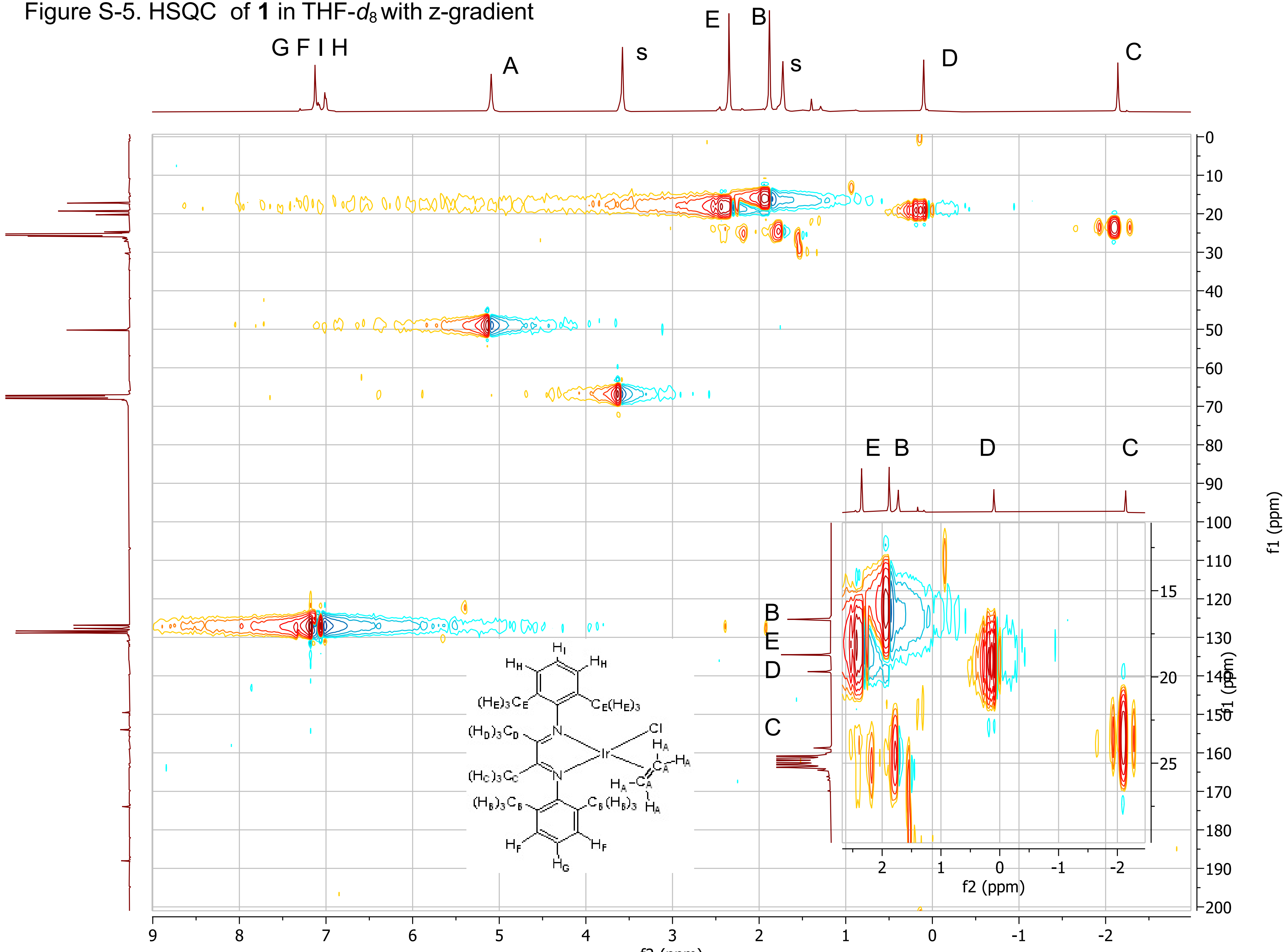

Figure S-6. NOESY of **1** in THF-d_8

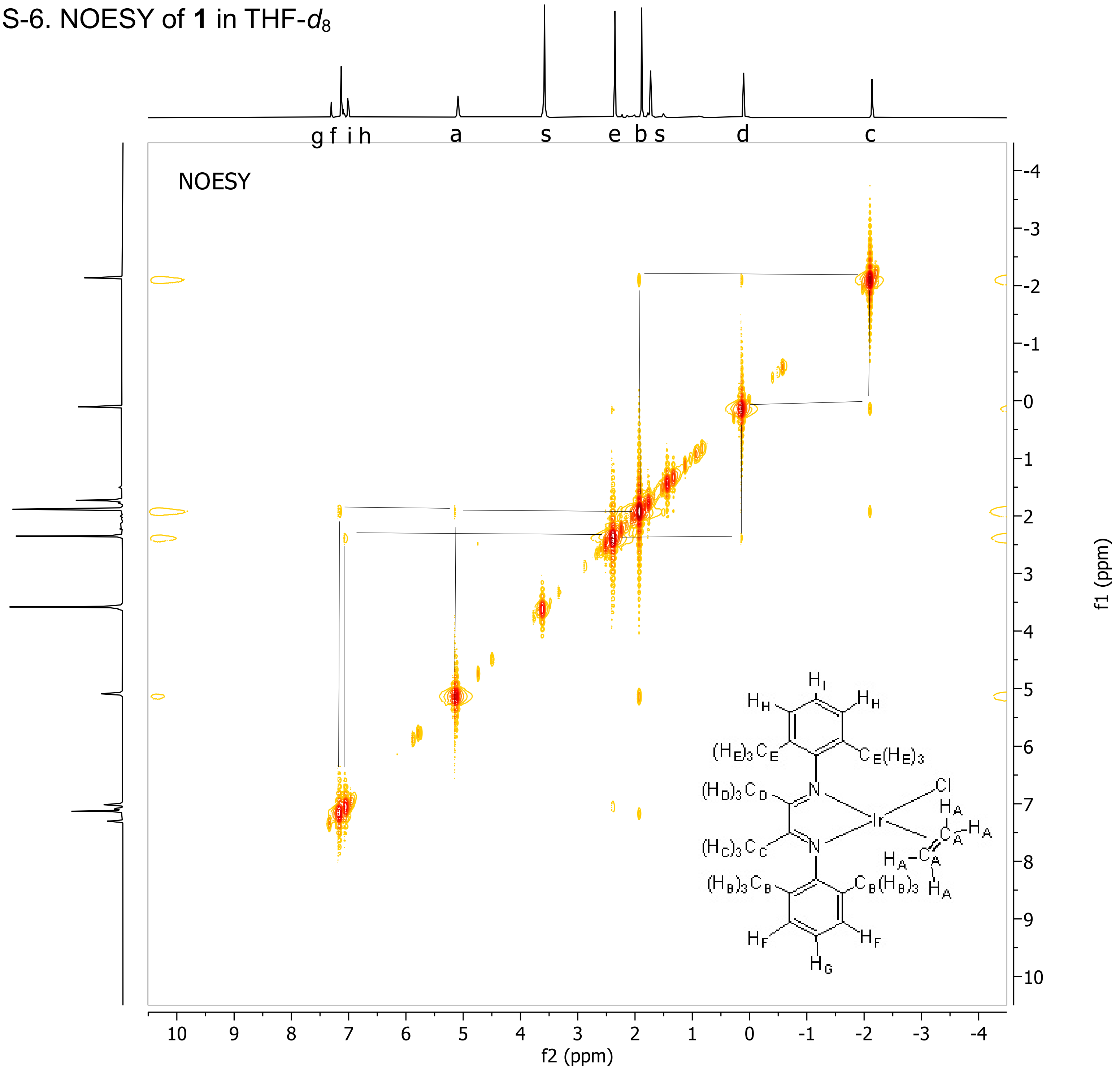

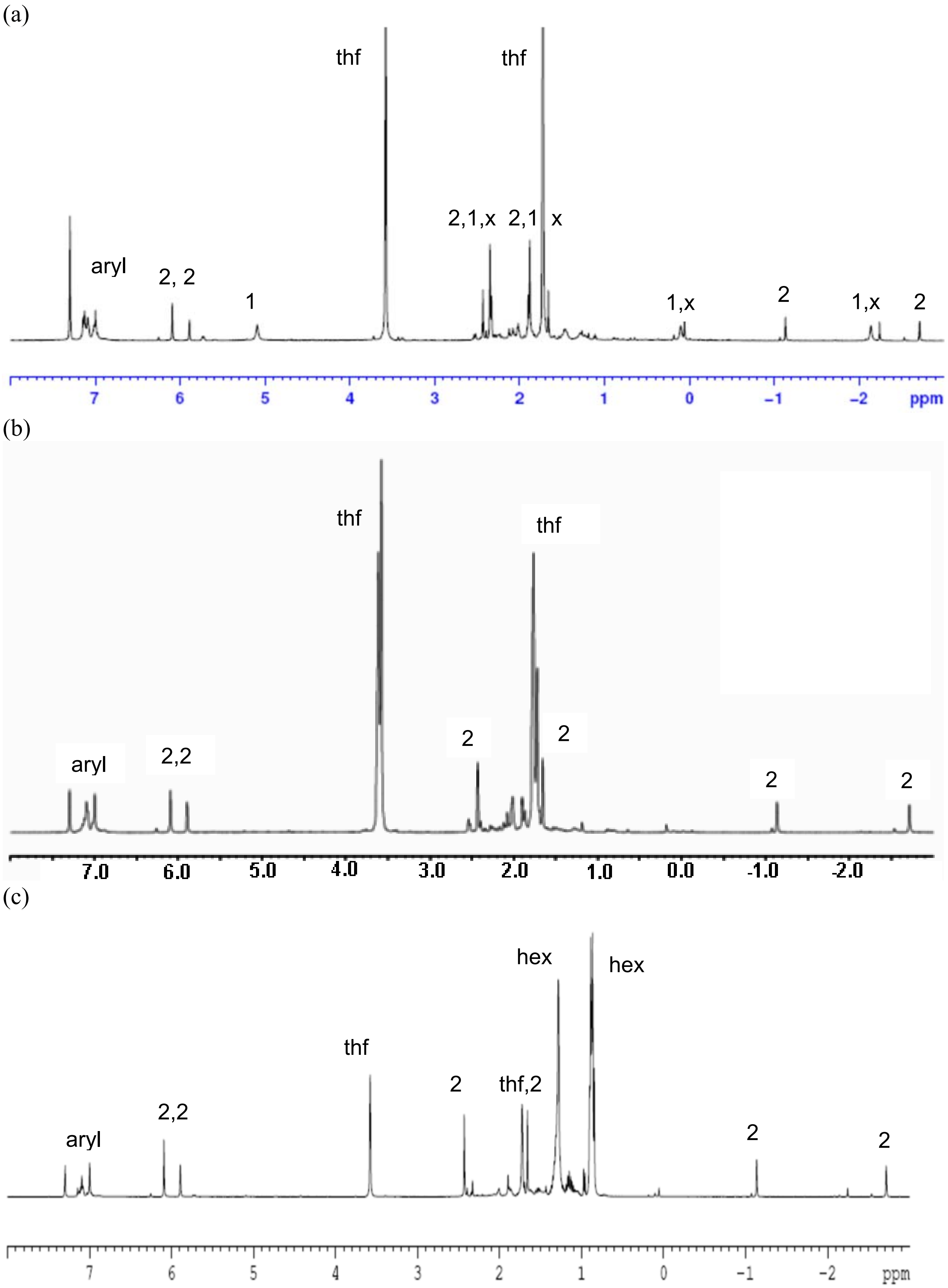

Figure S-7. ^{1}H NMR spectra of reaction of **1** in THF-d_8 with (a) MeLi, (b) MeMgCl in THF, and (c) Me_2Zn in hexane. Note that spectrum (a) contains unreacted **1** and a second product.

Figure S-8. ^{1}H NMR of **3** in THF-d_8
213904

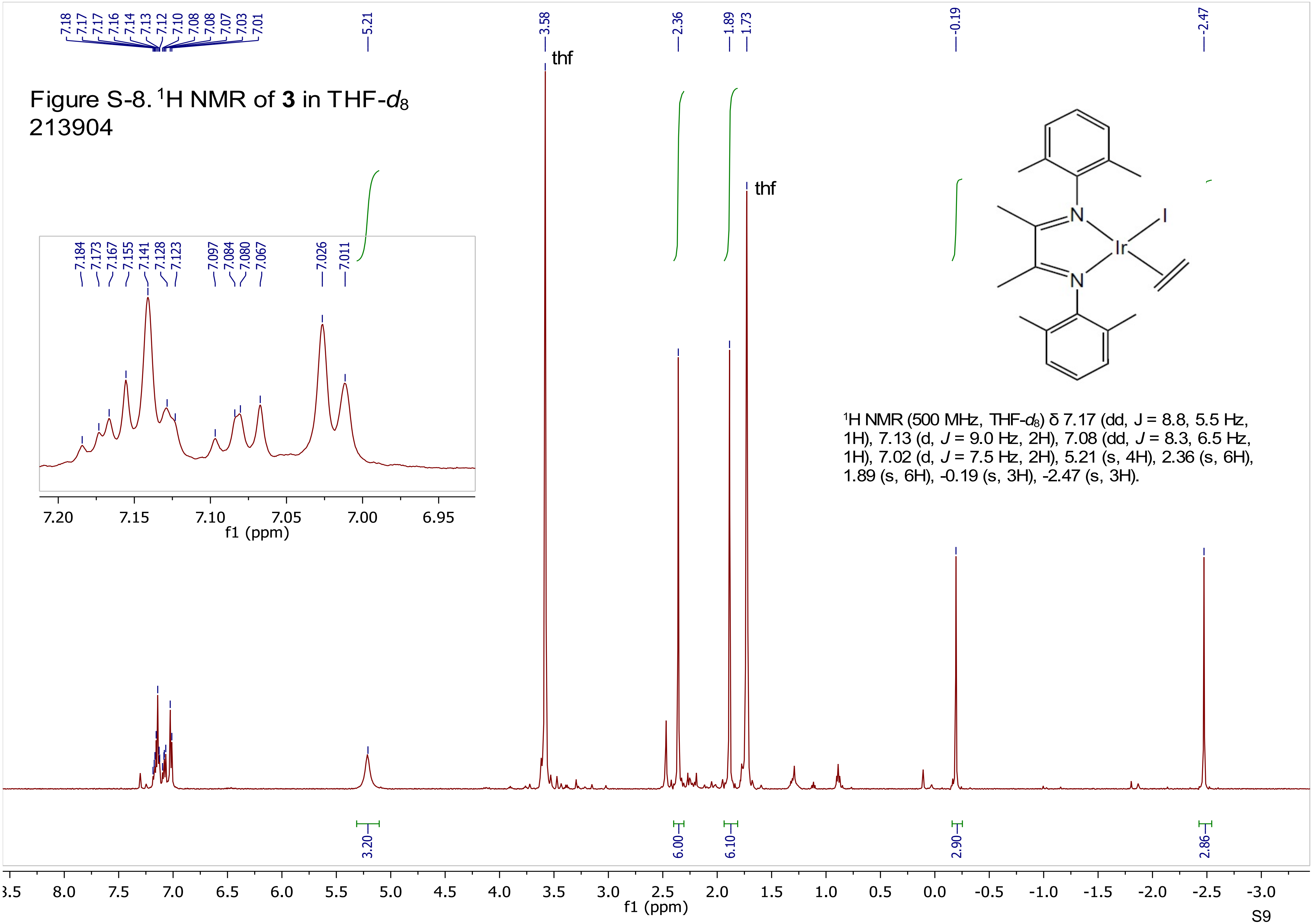

^{1}H NMR (500 MHz, THF-d_8) δ 7.17 (dd, J = 8.8, 5.5 Hz, 1H), 7.13 (d, *J* = 9.0 Hz, 2H), 7.08 (dd, *J* = 8.3, 6.5 Hz, 1H), 7.02 (d, *J* = 7.5 Hz, 2H), 5.21 (s, 4H), 2.36 (s, 6H), 1.89 (s, 6H), -0.19 (s, 3H), -2.47 (s, 3H).

Figure S-9. $^{13}C\{^{1}H\}$ NMR of **3** in THF-d_8
213906

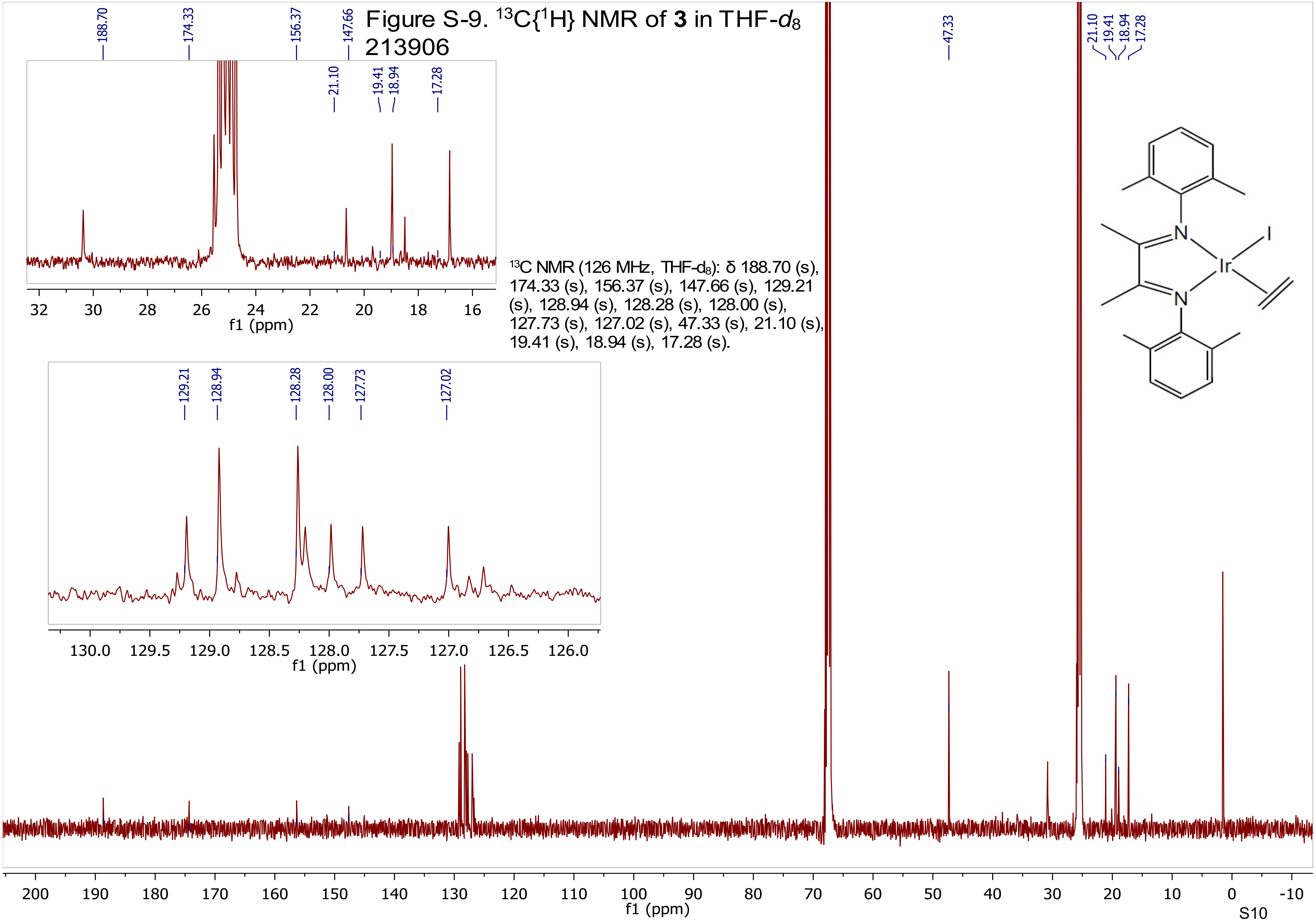

^{13}C NMR (126 MHz, THF-d$_8$): δ 188.70 (s), 174.33 (s), 156.37 (s), 147.66 (s), 129.21 (s), 128.94 (s), 128.28 (s), 128.00 (s), 127.73 (s), 127.02 (s), 47.33 (s), 21.10 (s), 19.41 (s), 18.94 (s), 17.28 (s).

Figure S-10. ^{1}H NMR of **4** in THF-d_8
213602

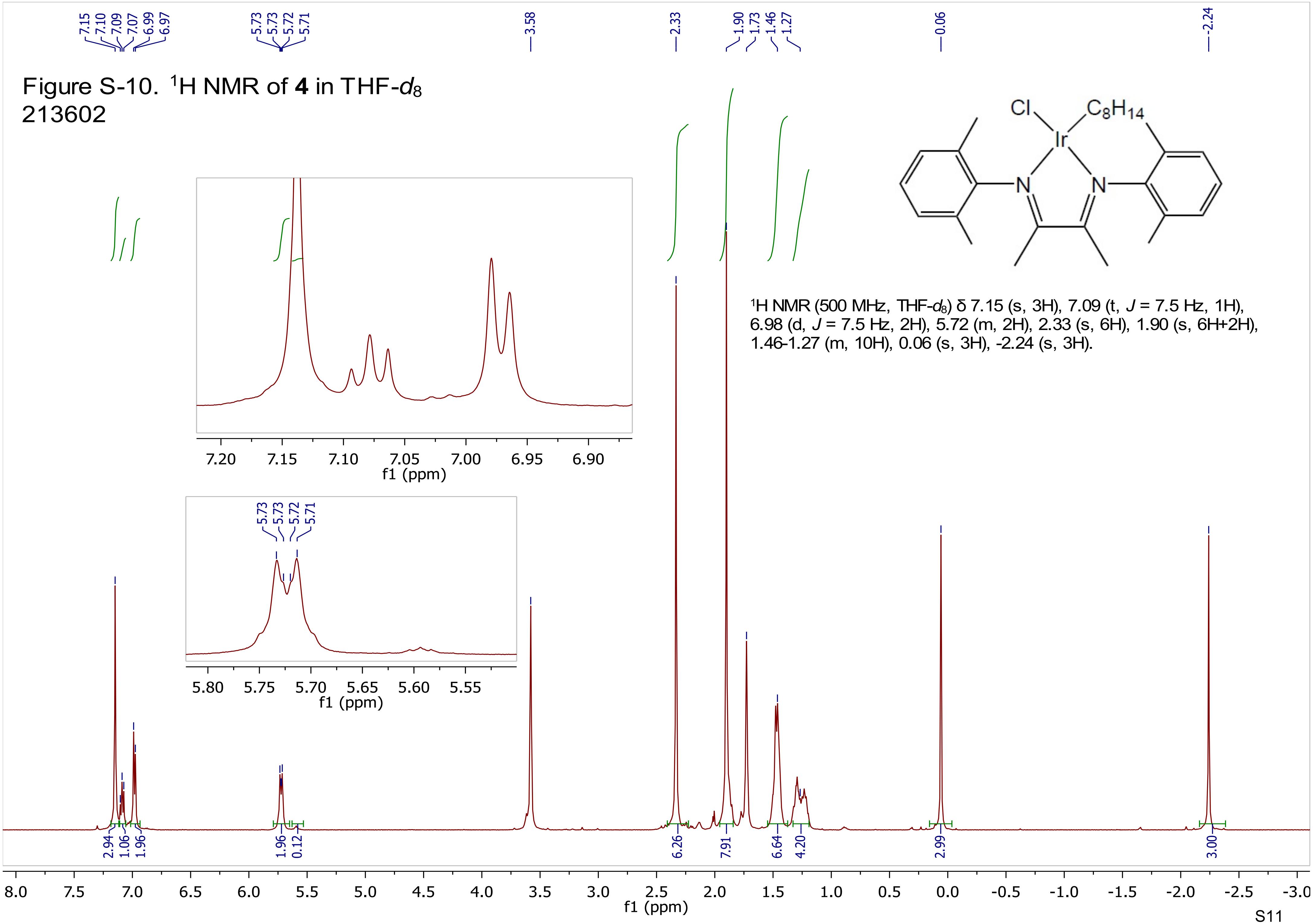

^{1}H NMR (500 MHz, THF-d_8) δ 7.15 (s, 3H), 7.09 (t, *J* = 7.5 Hz, 1H), 6.98 (d, *J* = 7.5 Hz, 2H), 5.72 (m, 2H), 2.33 (s, 6H), 1.90 (s, 6H+2H), 1.46-1.27 (m, 10H), 0.06 (s, 3H), -2.24 (s, 3H).

Figure S-11. $^{13}C\{H\}$ NMR of **4** in THF-d_8
213603

^{13}C NMR (126 MHz, THF-d_8): δ 186.47 (s), 172.80 (s), 154.98 (s), 149.27 (s), 129.33 (s), 128.79 (s), 128.18 (s), 128.12 (s), 127.62 (s), 126.62 (s), 70.44 (s), 31.75 (s), 29.71 (s), 27.67 (s), 25.04 (s), 20.16 (s), 19.36 (s), 17.43 (s).

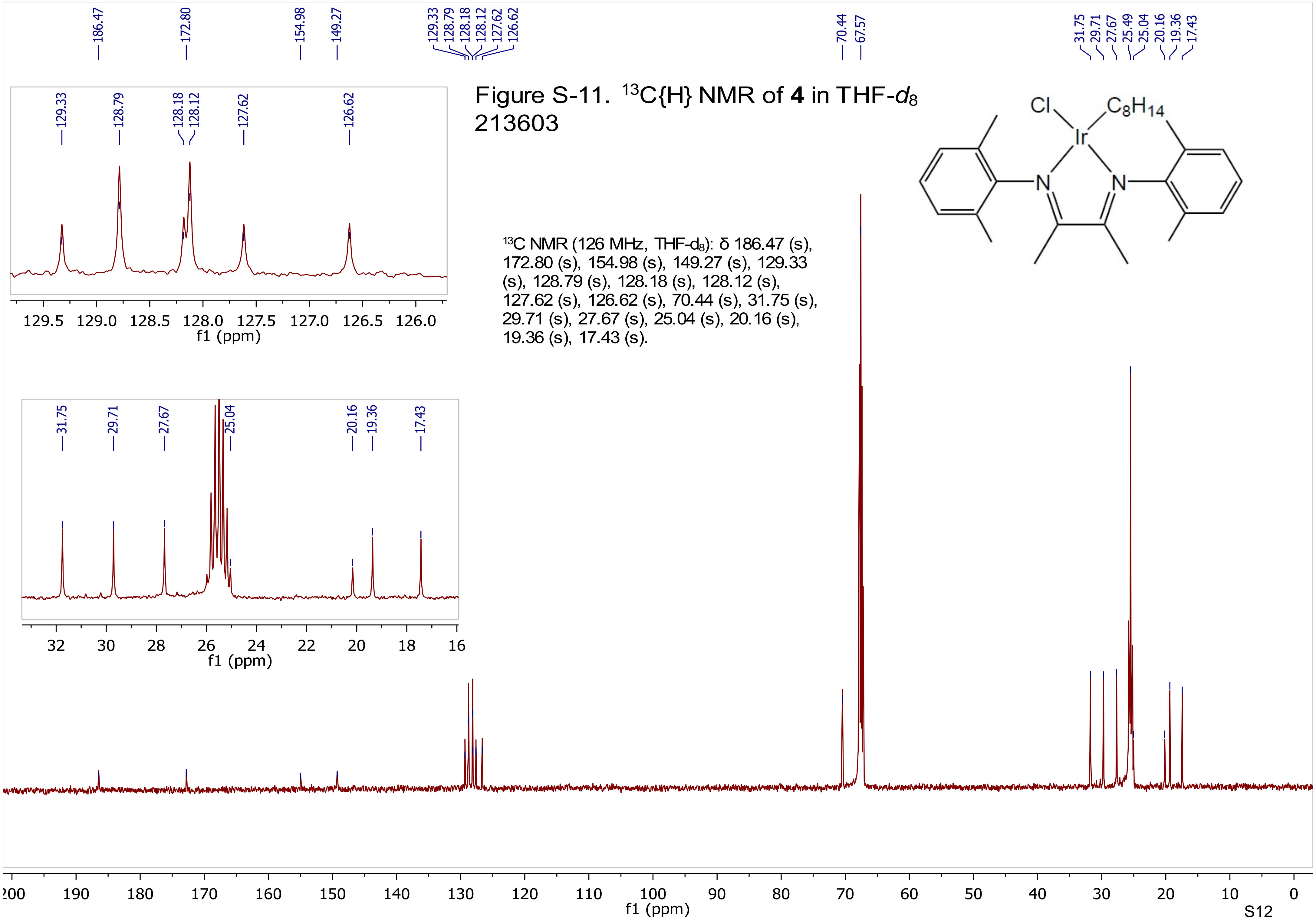

Figure S-12. ^{1}H NMR spectrum of **5** in THF-d_8.

^{1}H NMR (THF-d_8, 22 °C): δ 7.09 (m, 6H), 5.13 (s, 4H), 2.43 (s, 6H), 1.91 (s, 6H), 0.70 (s, 3H), -1.65 (s, 3H).

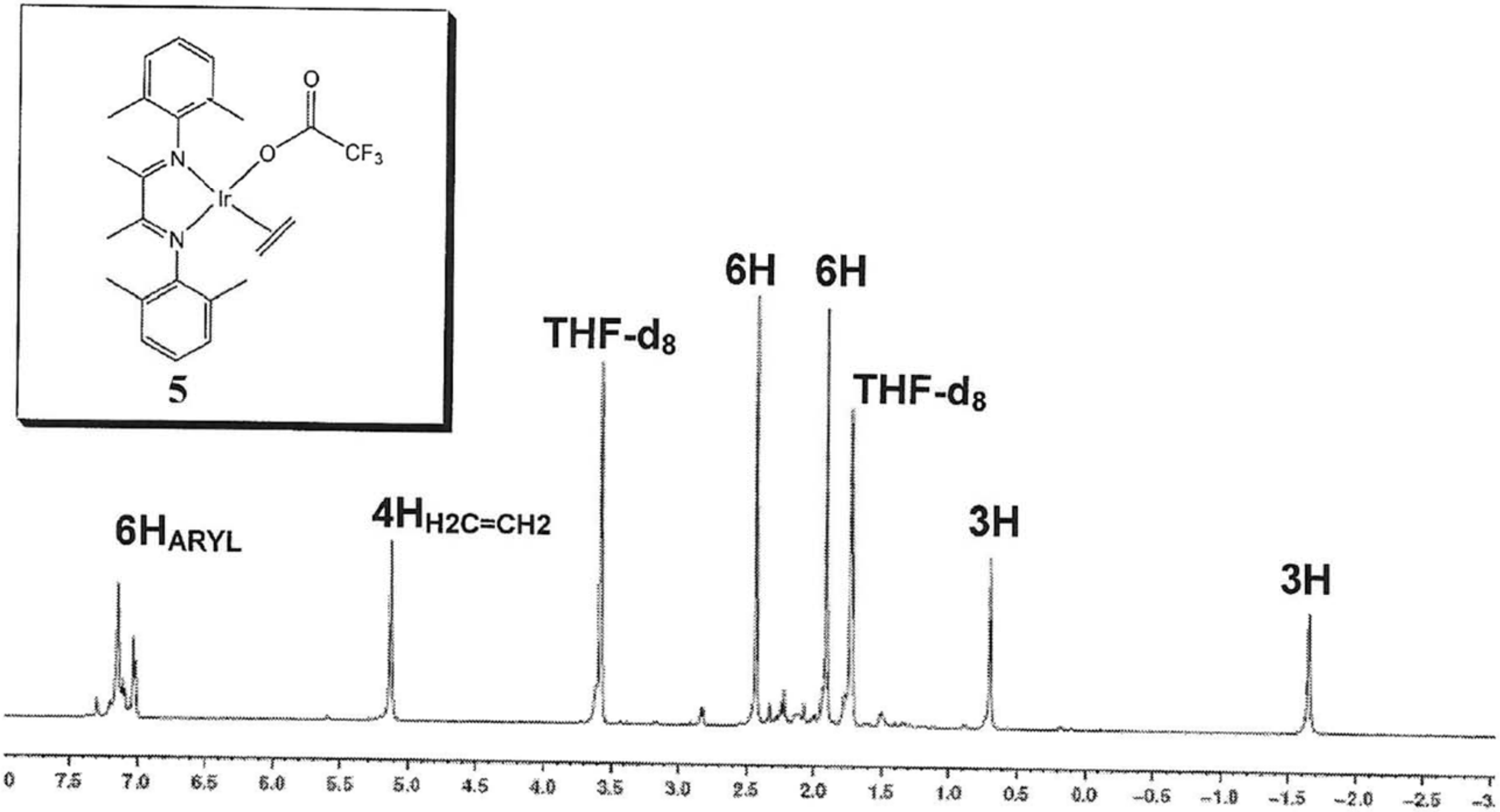

Figure S-13. ^{1}H NMR of **6** in THF-d_8

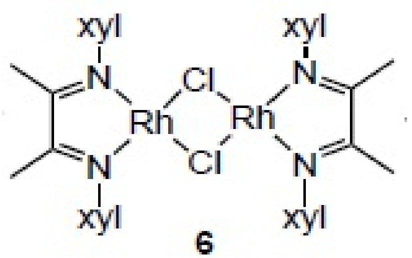

6

^{1}H NMR (500 MHz, THF-d_8): δ 7.11 (t, J = 7.5 Hz, 2H), 7.04 (d, *J* = 7.5 Hz, 4H), 2.18 (s, 12H), 0.00 (s, 6H).

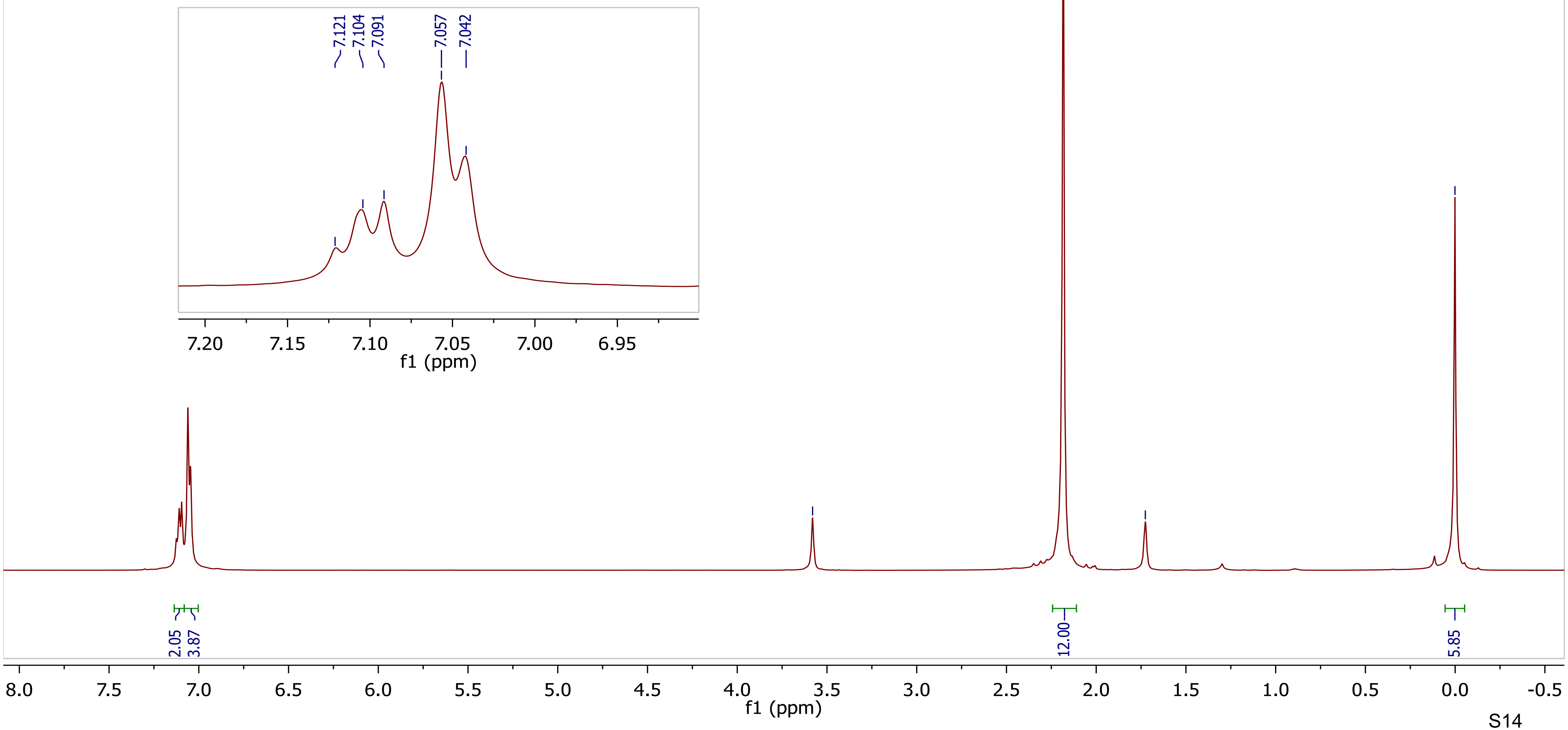

sl02s1.2 — THF-d8

Figure S-14. $^{13}C\{^1H\}$ NMR of **6** in THF-d_8

^{13}C NMR (126 MHz, THF-d_8) δ 157.81, 153.89, 130.55, 128.46, 126.07, 19.18, 18.04.

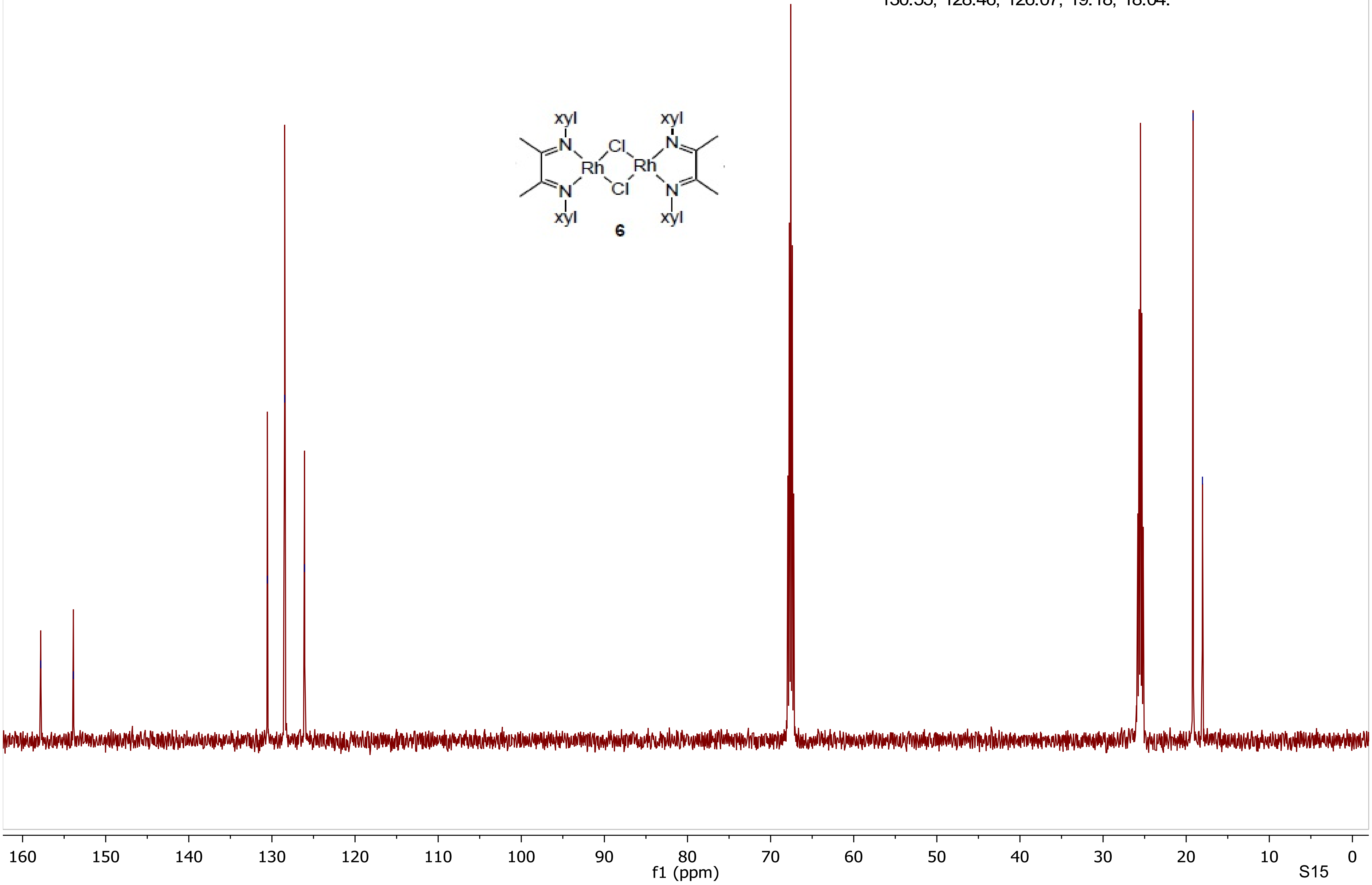

Figure S-15. ^{1}H NMR of **7** in THF-d_8

^{1}H NMR (400 MHz, THF-d_8, -80 °C): δ 7.20 (s, 4 H), 7.06 (s, 2 H), 3.05 (s, 4 H), 2.32 (s, 6 H), 2.11 (s, 6 H), 1.66 (s, 3 H), 0.62 (s, 3 H).

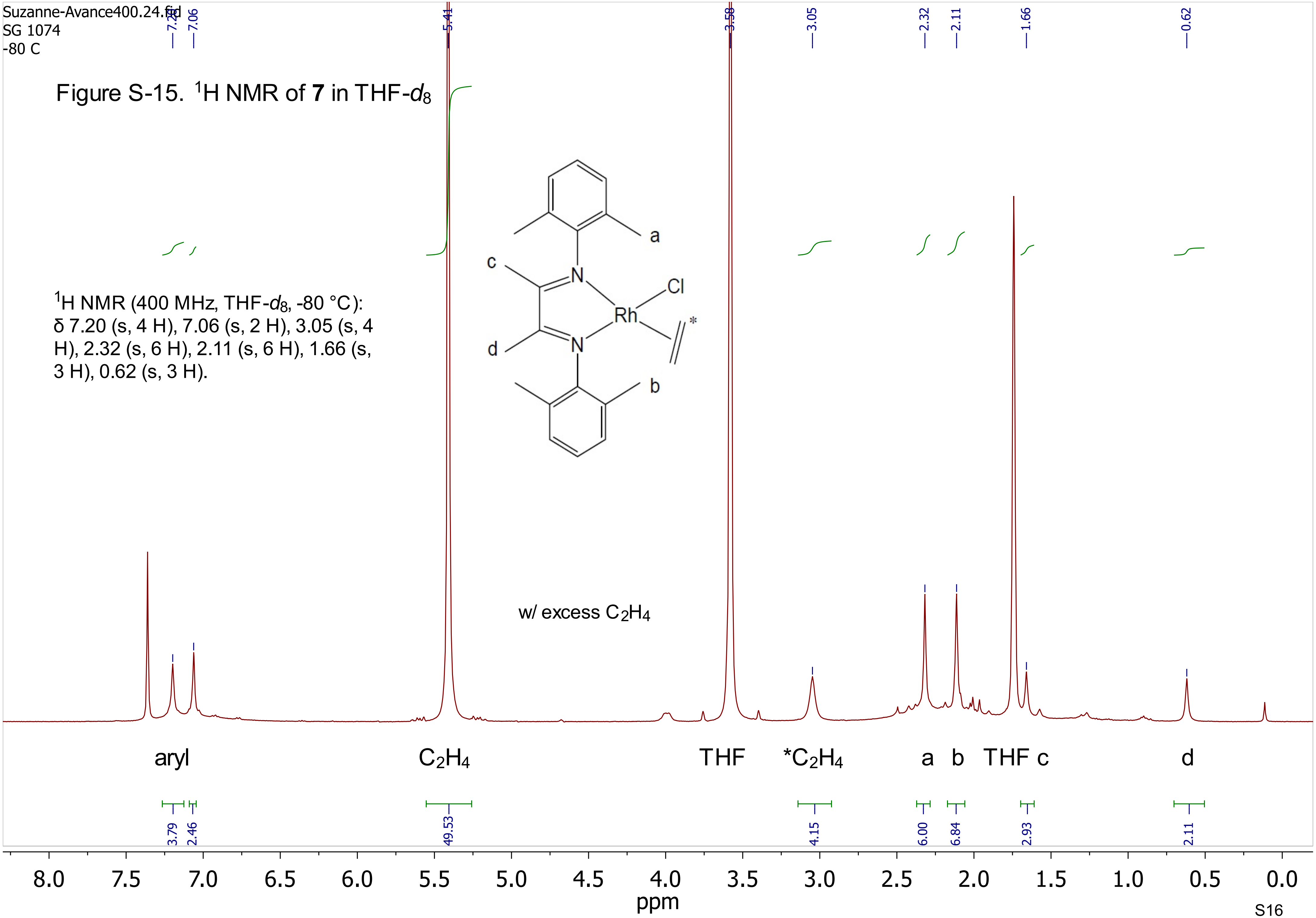

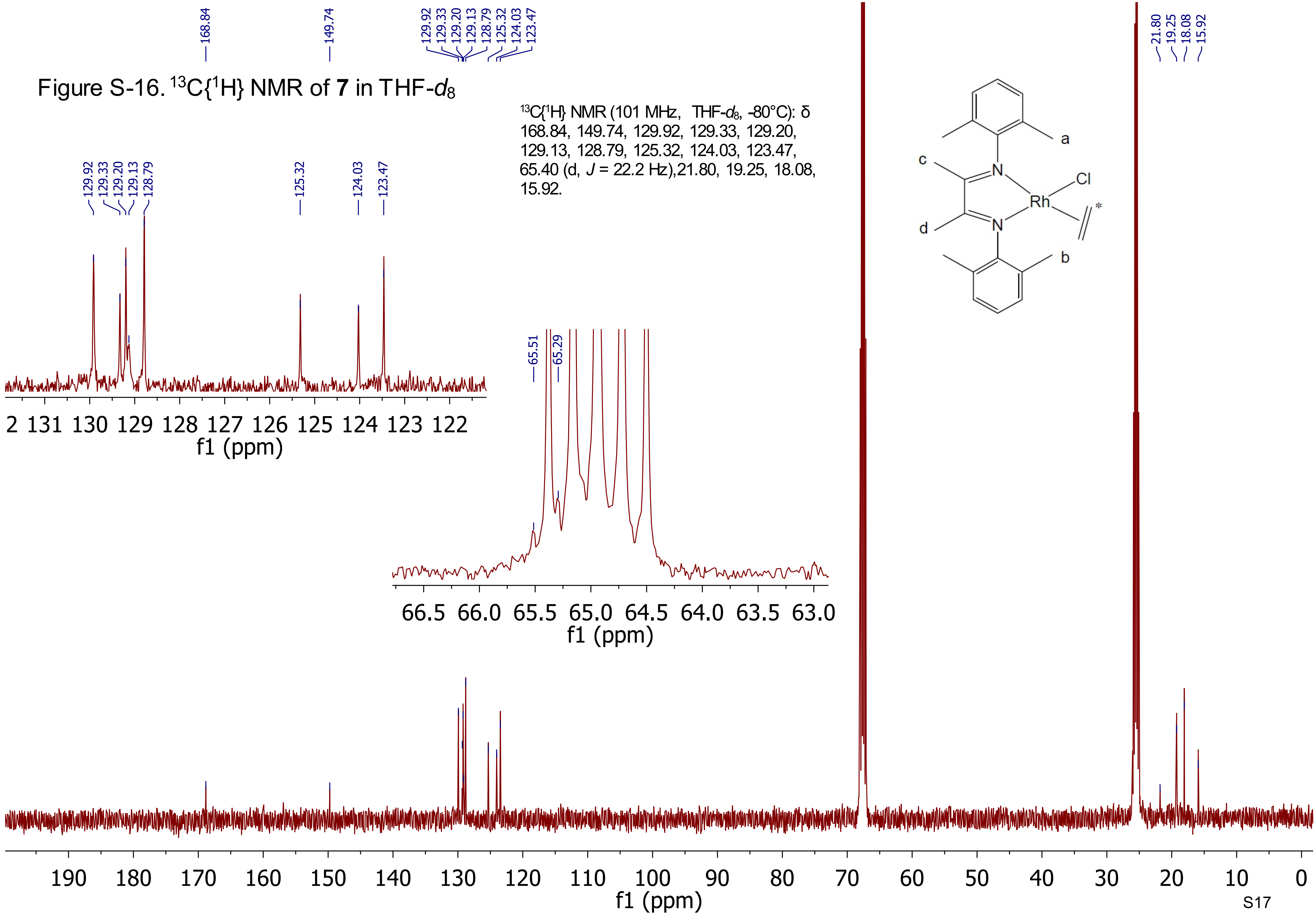

Figure S-16. $^{13}C\{^1H\}$ NMR of **7** in THF-d_8

$^{13}C\{^1H\}$ NMR (101 MHz, THF-d_8, -80°C): δ 168.84, 149.74, 129.92, 129.33, 129.20, 129.13, 128.79, 125.32, 124.03, 123.47, 65.40 (d, J = 22.2 Hz),21.80, 19.25, 18.08, 15.92.

Figure S-17. VT ^1H NMR of **7** in THF-d_8

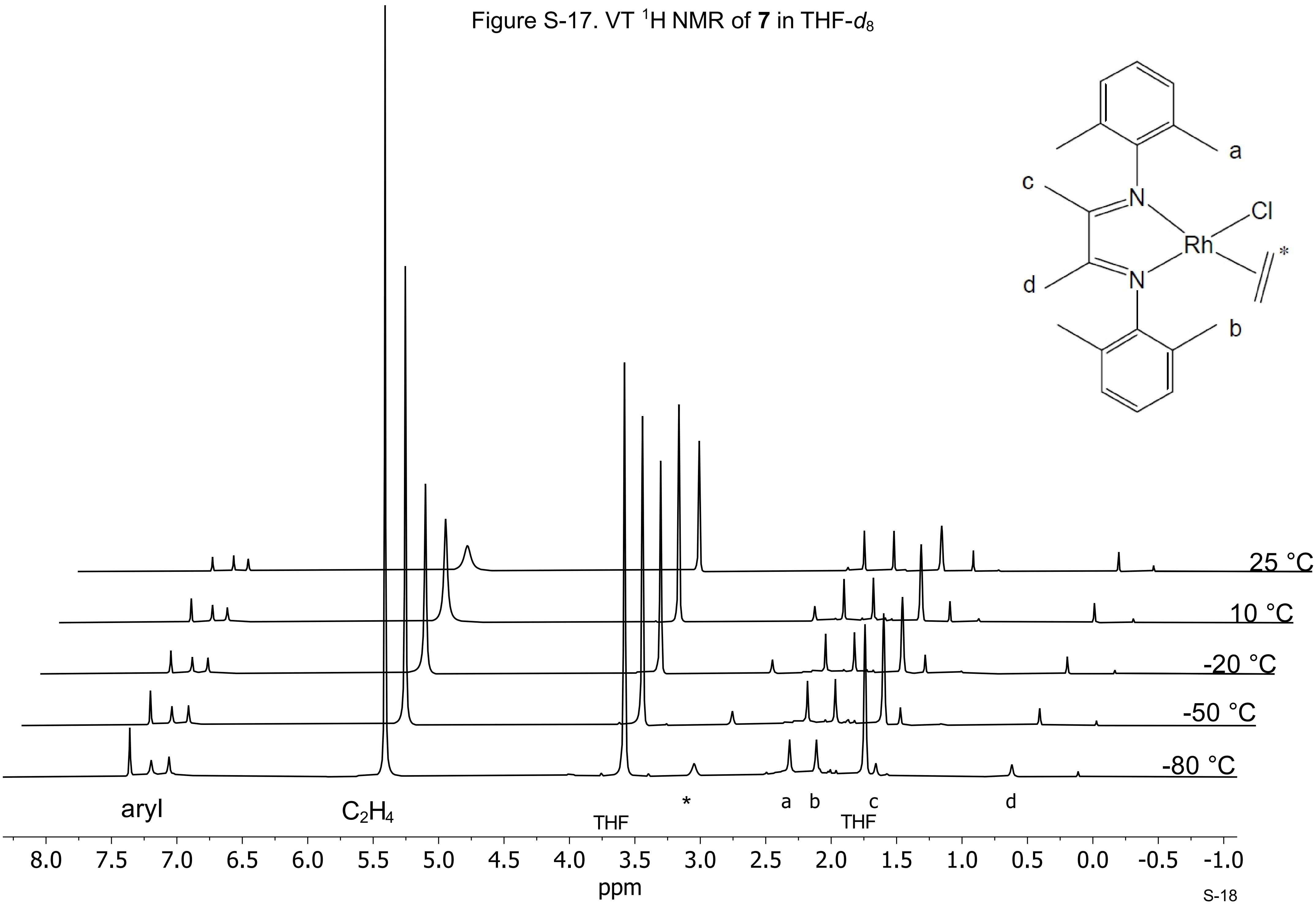

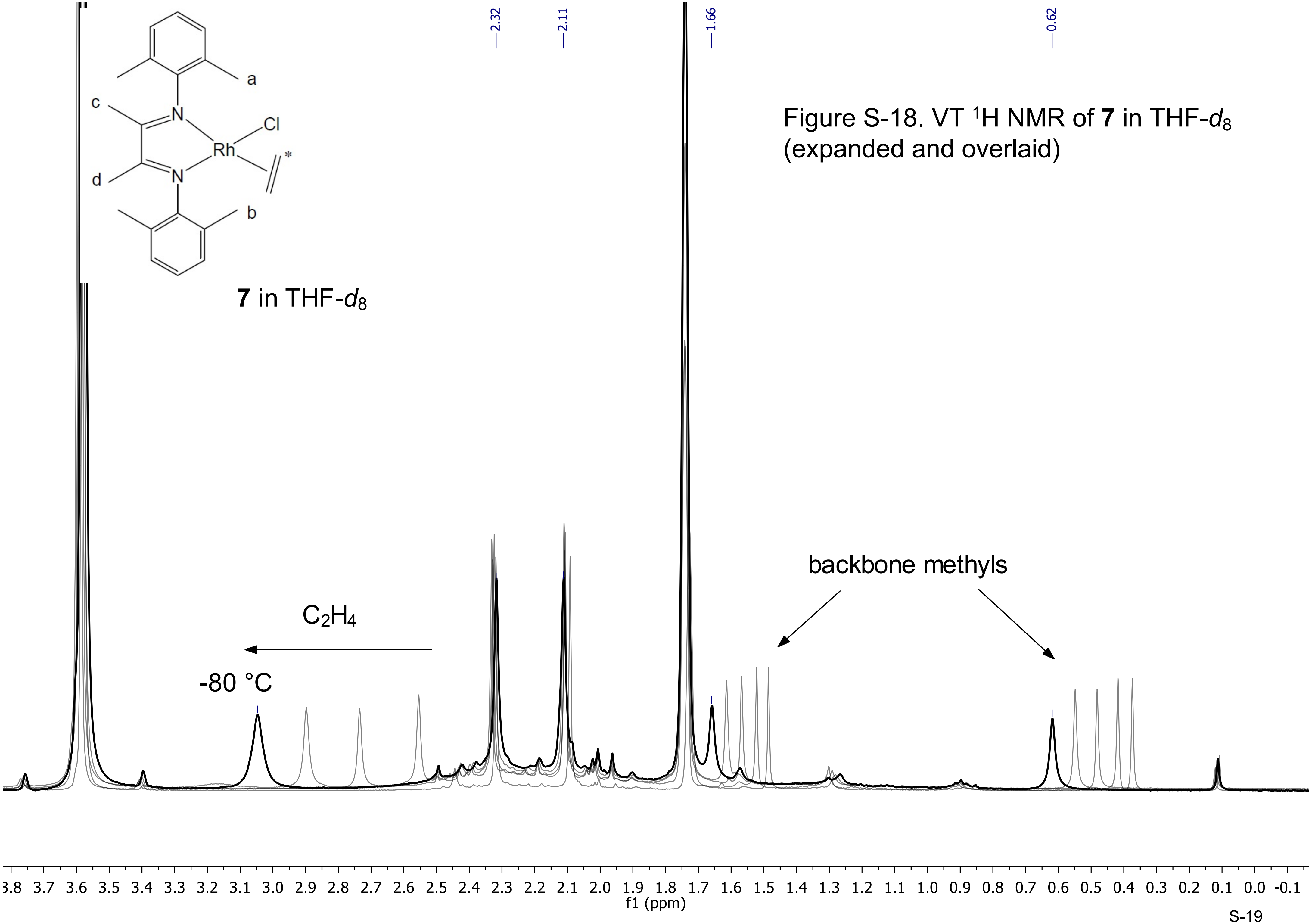

Figure S-18. VT ^{1}H NMR of **7** in THF-d_8 (expanded and overlaid)

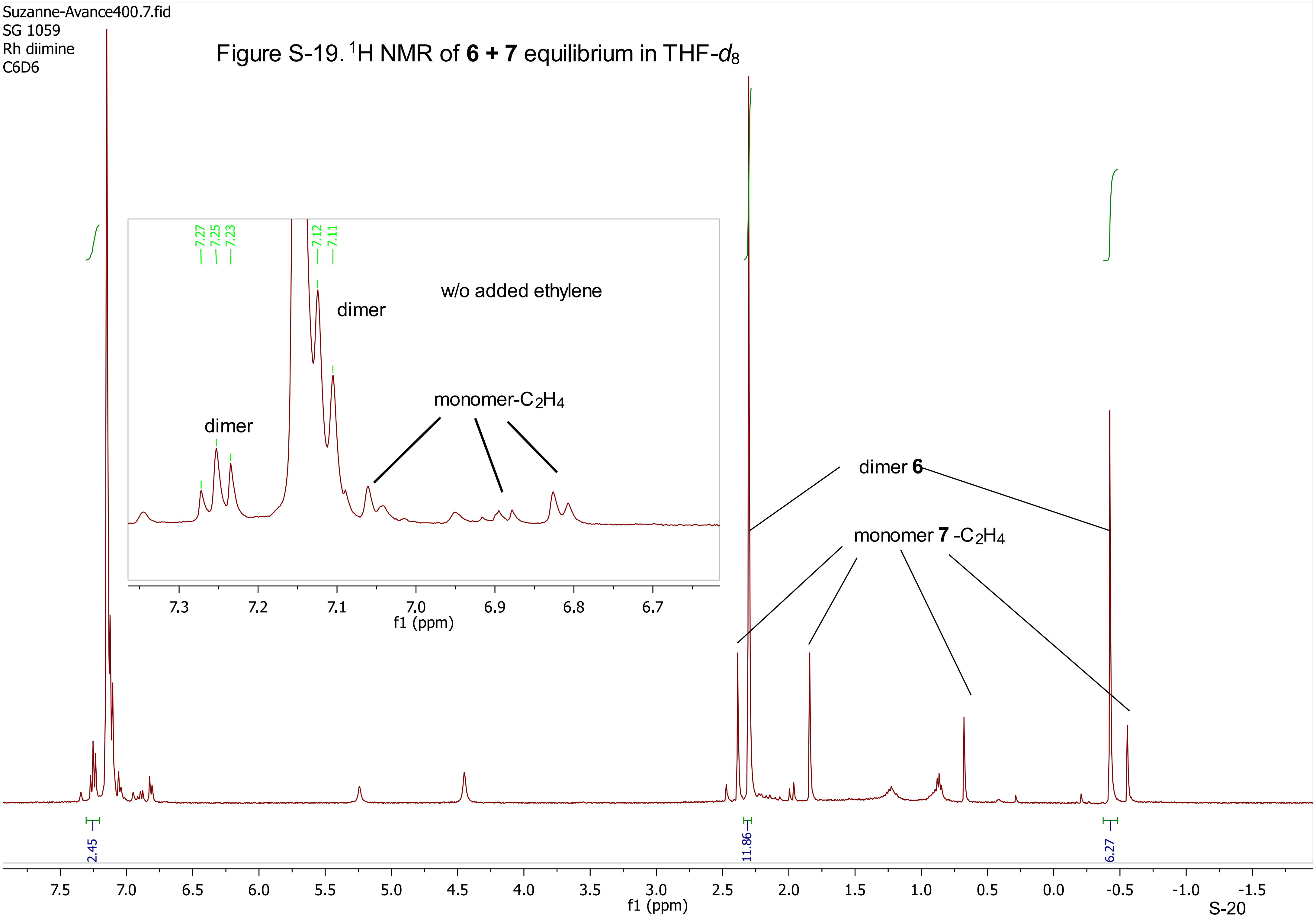
Suzanne-Avance400.7.fid
SG 1059
Rh diimine
C6D6
Figure S-19. ^{1}H NMR of **6 + 7** equilibrium in THF-d_8
7.27
7.25
7.23
7.12
7.11
w/o added ethylene
dimer
monomer-C_2H_4
dimer
7.3
7.2
7.1
7.0
6.9
6.8
6.7
f1 (ppm)
dimer **6**
monomer **7** -C_2H_4
2.45
11.86
6.27
7.5
7.0
6.5
6.0
5.5
5.0
4.5
4.0
3.5
3.0
2.5
2.0
1.5
1.0
0.5
0.0
-0.5
-1.0
-1.5
f1 (ppm)

REFERENCE NUMBER: jonjk02

1

CRYSTAL STRUCTURE REPORT

C_{22} H_{28} Cl Ir N_2

or

$Ir(C_{20}H_{24}N_2)(C_2H_4)Cl$

Report prepared for:

J. Kovach, Prof. W. Jones

May 27, 2006

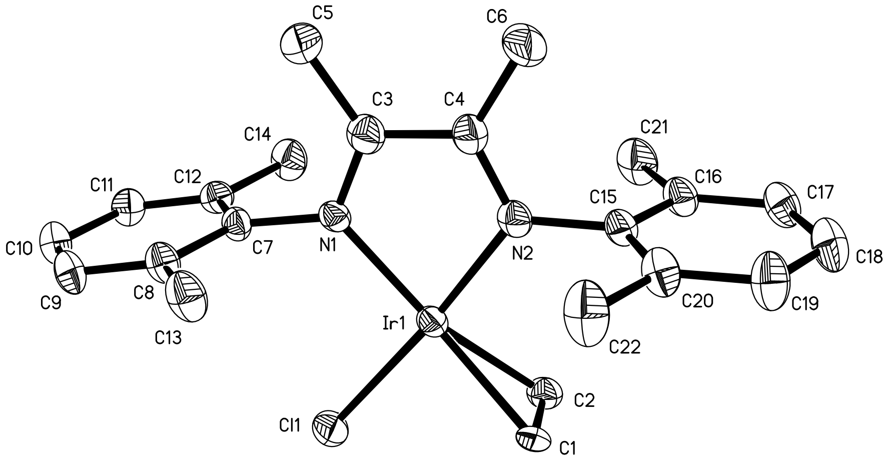

Figure S-20. X-ray Structure of $IrCl(C_2H_4)$(α-diimine), **1** (hydrogens omitted).

William W. Brennessel

X-ray Crystallographic Facility

Department of Chemistry

University of Rochester

120 Trustee Road

Rochester, NY 14627

Data collection

A crystal (0.22 x 0.08 x 0.01 mm^3) was placed onto the tip of a 0.1 mm diameter glass capillary tube or fiber and mounted on a Bruker SMART APEX II CCD Platform diffractometer for a data collection at 100.0(1) K.[1] A preliminary set of cell constants and an orientation matrix were calculated from 393 reflections harvested from three sets of 20 frames. These initial sets of frames were oriented such that orthogonal wedges of reciprocal space were surveyed. The data collection was carried out using MoKα radiation (graphite monochromator) with a frame time of 60 seconds and a detector distance of 4.92 cm. A randomly oriented region of reciprocal space was surveyed to the extent of one sphere and to a resolution of 0.70 Å. Four major sections of frames were collected with 0.50º steps in ω at four different ϕ settings and a detector position of -28º in 2θ. The intensity data were corrected for absorption.[2] Final cell constants were calculated from the xyz centroids of 3883 strong reflections from the actual data collection after integration.[3] See Table S-1 for additional crystal and refinement information.

Structure solution and refinement

The structure was solved using SIR97[4] and refined using SHELXL-97.[5] The space group $P2_1/c$ was determined based on systematic absences and intensity statistics. A direct-methods solution was calculated which provided most non-hydrogen atoms from the E-map. Full-matrix least squares / difference Fourier cycles were performed which located the remaining non-hydrogen atoms. All non-hydrogen atoms were refined with anisotropic displacement parameters. All hydrogen atoms were placed in ideal positions and refined as riding atoms with relative isotropic displacement parameters. The final full matrix least squares refinement converged to $R1 = 0.0450$ (F^2, $I > 2\sigma(I)$) and $wR2 = 0.0981$ (F^2, all data).

Structure description

The structure is the one suggested. All atoms lie on general positions. The chloride and ethylene ligands are modeled as disordered with each other (52:48).

Data collection, structure solution, and structure refinement were conducted at the X-ray Crystallographic Facility, B51 Hutchison Hall, Department of Chemistry, University of Rochester.

[1] APEX2 V2.0-2, Bruker Analytical X-ray Systems, Madison, WI (2005).

[2] SADABS 2004/1, An empirical correction for absorption anisotropy, R. Blessing, *Acta Cryst.* **A51**, 33-38 (1995).

[3] SAINT V7.23A, Bruker Analytical X-ray Systems, Madison, WI (2005).

[4] SIR97, A new tool for crystal structure determination and refinement, A. Altomare, M. C. Burla, M. Camalli, G. Cascarano, C. Giacovazzo, A. Guagliardi, A. G. G. Moliterni, G. Polidori, R. Spagna. *J. Appl. Cryst.* **32**, 115-119 (1998).

[5] SHELXTL V6.14, Bruker Analytical X-ray Systems, Madison, WI (2000).

Some equations of interest:

$$R_{int} = \Sigma|F_o^2 - \langle F_o^2\rangle| / \Sigma|F_o^2|$$
$$R_1 = \Sigma||F_o| - |F_c|| / \Sigma|F_o|$$
$$wR2 = [\Sigma[w(F_o^2 - F_c^2)^2] / \Sigma[w(F_o^2)^2]]^{1/2}$$
$$\text{where } w = q / [\sigma^2(F_o^2) + (a*P)^2 + b*P + d + e*\sin(\theta)]$$
$$\text{GooF} = S = [\Sigma[w(F_o^2 - F_c^2)^2] / (n-p)]^{1/2}$$

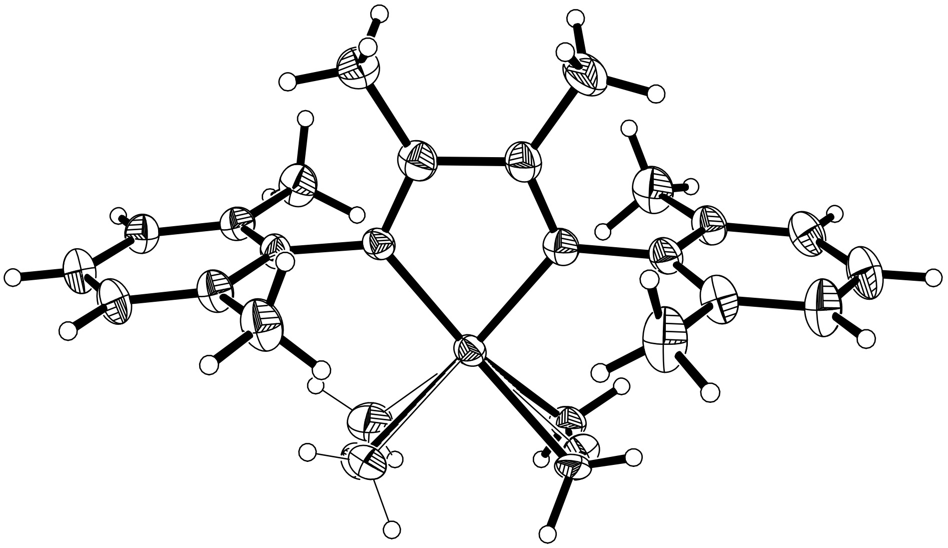

Figure S-21. X-ray Structure of $IrCl(C_2H_4)$(α-diimine), **1** (with hydrogens).

Table S-1. Crystal data and structure refinement for **1**.

Identification code	jonjk02	
Empirical formula	C22 H28 Cl Ir N2	
Formula weight	548.11	
Temperature	100.0(1) K	
Wavelength	0.71073 Å	
Crystal system	Monoclinic	
Space group	$P2_1/c$	
Unit cell dimensions	a = 8.233(2) Å	α = 90°
	b = 13.236(3) Å	β = 98.776(4)°
	c = 19.282(5) Å	γ = 90°
Volume	2076.7(9) $Å^3$	
Z	4	
Density (calculated)	1.753 Mg/m^3	
Absorption coefficient	6.566 mm^{-1}	
F(000)	1072	
Crystal color, morphology	colorless, plate	
Crystal size	0.22 x 0.08 x 0.01 mm^3	
Theta range for data collection	1.87 to 30.51°	
Index ranges	$-11 \le h \le 11, -18 \le k \le 18, -27 \le l \le 27$	
Reflections collected	32022	
Independent reflections	6322 [R(int) = 0.0560]	
Observed reflections	5048	
Completeness to theta = 30.51°	99.8%	
Absorption correction	Multi-scan	
Max. and min. transmission	0.9392 and 0.3960	
Refinement method	Full-matrix least-squares on F^2	
Data / restraints / parameters	6322 / 0 / 269	
Goodness-of-fit on F^2	1.078	
Final R indices [I>2sigma(I)]	$R1$ = 0.0450, $wR2$ = 0.0923	
R indices (all data)	$R1$ = 0.0624, $wR2$ = 0.0981	
Largest diff. peak and hole	2.798 and -3.161 e.$Å^{-3}$	

Table S-2. Atomic coordinates (x 10^4) and equivalent isotropic displacement parameters ($Å^2$x 10^3) for **1**. U_{eq} is defined as one third of the trace of the orthogonalized U_{ij} tensor.

	x	y	z	U_{eq}
Ir1	7812(1)	848(1)	7794(1)	22(1)
Cl1	8793(6)	2365(3)	7438(2)	32(1)
C1	8530(20)	1330(13)	8870(7)	37(4)
C2	6900(20)	1605(14)	8628(11)	27(3)
Cl1'	7486(7)	1605(4)	8836(3)	35(2)
C1'	7790(40)	2318(13)	7321(10)	45(4)
C2'	9340(30)	2036(19)	7544(11)	39(4)
N1	7825(5)	88(4)	6886(3)	20(1)
N2	7121(6)	-542(4)	8042(3)	24(1)
C3	7410(7)	-881(5)	6884(3)	30(1)
C4	7016(8)	-1247(5)	7541(4)	30(1)
C5	7309(10)	-1551(6)	6248(4)	43(2)
C6	6597(11)	-2331(5)	7662(4)	45(2)
C7	8252(7)	538(4)	6258(3)	22(1)
C8	9904(7)	539(5)	6149(3)	26(1)
C9	10300(7)	1011(5)	5555(3)	29(1)
C10	9092(8)	1457(5)	5069(4)	30(1)
C11	7461(7)	1448(5)	5181(3)	28(1)
C12	7009(7)	994(4)	5775(3)	24(1)
C13	11203(7)	37(6)	6682(4)	35(2)
C14	5238(7)	972(6)	5895(4)	34(2)
C15	6758(7)	-835(5)	8726(3)	26(1)
C16	5128(7)	-765(5)	8857(3)	28(1)
C17	4806(8)	-1058(5)	9520(4)	32(1)
C18	6057(9)	-1391(5)	10029(4)	37(2)
C19	7671(9)	-1429(6)	9890(4)	38(2)
C20	8048(8)	-1151(5)	9236(4)	30(1)
C21	3790(8)	-360(6)	8308(4)	41(2)
C22	9793(8)	-1185(7)	9077(4)	44(2)

Table S-3. Bond lengths [Å] and angles [°] for **1**.

Ir(1)-N(2)	2.005(5)	C(8)-C(13)	1.518(9)
Ir(1)-N(1)	2.021(5)	C(9)-C(10)	1.389(9)
Ir(1)-C(2')	2.12(2)	C(9)-H(9A)	0.9500
Ir(1)-C(2)	2.13(2)	C(10)-C(11)	1.392(8)
Ir(1)-C(1')	2.147(17)	C(10)-H(10A)	0.9500
Ir(1)-C(1)	2.165(13)	C(11)-C(12)	1.394(8)
Ir(1)-Cl(1')	2.298(5)	C(11)-H(11A)	0.9500
Ir(1)-Cl(1)	2.307(5)	C(12)-C(14)	1.511(8)
C(1)-C(2)	1.40(3)	C(13)-H(13A)	0.9800
C(1)-H(1A)	0.9900	C(13)-H(13B)	0.9800
C(1)-H(1B)	0.9900	C(13)-H(13C)	0.9800
C(2)-H(2A)	0.9900	C(14)-H(14A)	0.9800
C(2)-H(2B)	0.9900	C(14)-H(14B)	0.9800
C(1')-C(2')	1.34(4)	C(14)-H(14C)	0.9800
C(1')-H(1C)	0.9900	C(15)-C(20)	1.397(9)
C(1')-H(1D)	0.9900	C(15)-C(16)	1.405(7)
C(2')-H(2C)	0.9900	C(16)-C(17)	1.399(9)
C(2')-H(2D)	0.9900	C(16)-C(21)	1.505(9)
N(1)-C(3)	1.327(8)	C(17)-C(18)	1.383(10)
N(1)-C(7)	1.440(7)	C(17)-H(17A)	0.9500
N(2)-C(4)	1.336(8)	C(18)-C(19)	1.396(9)
N(2)-C(15)	1.451(8)	C(18)-H(18A)	0.9500
C(3)-C(4)	1.439(9)	C(19)-C(20)	1.394(9)
C(3)-C(5)	1.506(9)	C(19)-H(19A)	0.9500
C(4)-C(6)	1.503(9)	C(20)-C(22)	1.515(9)
C(5)-H(5A)	0.9800	C(21)-H(21A)	0.9800
C(5)-H(5B)	0.9800	C(21)-H(21B)	0.9800
C(5)-H(5C)	0.9800	C(21)-H(21C)	0.9800
C(6)-H(6A)	0.9800	C(22)-H(22A)	0.9800
C(6)-H(6B)	0.9800	C(22)-H(22B)	0.9800
C(6)-H(6C)	0.9800	C(22)-H(22C)	0.9800
C(7)-C(8)	1.407(8)	N(2)-Ir(1)-N(1)	77.8(2)
C(7)-C(12)	1.410(8)	N(2)-Ir(1)-C(2')	159.5(8)
C(8)-C(9)	1.386(9)	N(1)-Ir(1)-C(2')	95.2(6)

N(2)-Ir(1)-C(2)	96.1(5)
N(1)-Ir(1)-C(2)	159.9(5)
C(2')-Ir(1)-C(2)	96.5(8)
N(2)-Ir(1)-C(1')	161.5(7)
N(1)-Ir(1)-C(1')	94.8(5)
C(2')-Ir(1)-C(1')	36.6(11)
C(2)-Ir(1)-C(1')	85.0(7)
N(2)-Ir(1)-C(1)	95.1(4)
N(1)-Ir(1)-C(1)	160.0(6)
C(2')-Ir(1)-C(1)	85.0(7)
C(2)-Ir(1)-C(1)	38.2(8)
C(1')-Ir(1)-C(1)	97.0(6)
N(2)-Ir(1)-Cl(1')	96.7(2)
N(1)-Ir(1)-Cl(1')	172.61(19)
C(2')-Ir(1)-Cl(1')	91.6(6)
C(2)-Ir(1)-Cl(1')	14.4(5)
C(1')-Ir(1)-Cl(1')	89.0(5)
C(1)-Ir(1)-Cl(1')	23.8(6)
N(2)-Ir(1)-Cl(1)	173.92(18)
N(1)-Ir(1)-Cl(1)	97.33(18)
C(2')-Ir(1)-Cl(1)	15.9(7)
C(2)-Ir(1)-Cl(1)	89.7(5)
C(1')-Ir(1)-Cl(1)	20.9(7)
C(1)-Ir(1)-Cl(1)	88.3(4)
Cl(1')-Ir(1)-Cl(1)	88.47(17)
C(2)-C(1)-Ir(1)	69.5(11)
C(2)-C(1)-H(1A)	116.7
Ir(1)-C(1)-H(1A)	116.7
C(2)-C(1)-H(1B)	116.7
Ir(1)-C(1)-H(1B)	116.7
H(1A)-C(1)-H(1B)	113.7
C(1)-C(2)-Ir(1)	72.4(9)
C(1)-C(2)-H(2A)	116.3
Ir(1)-C(2)-H(2A)	116.3
C(1)-C(2)-H(2B)	116.3
Ir(1)-C(2)-H(2B)	116.3
H(2A)-C(2)-H(2B)	113.3
C(2')-C(1')-Ir(1)	70.5(12)
C(2')-C(1')-H(1C)	116.6
Ir(1)-C(1')-H(1C)	116.6
C(2')-C(1')-H(1D)	116.6
Ir(1)-C(1')-H(1D)	116.6
H(1C)-C(1')-H(1D)	113.6
C(1')-C(2')-Ir(1)	73.0(11)
C(1')-C(2')-H(2C)	116.3
Ir(1)-C(2')-H(2C)	116.3
C(1')-C(2')-H(2D)	116.3
Ir(1)-C(2')-H(2D)	116.3
H(2C)-C(2')-H(2D)	113.2
C(3)-N(1)-C(7)	119.6(5)
C(3)-N(1)-Ir(1)	116.6(4)
C(7)-N(1)-Ir(1)	123.8(4)
C(4)-N(2)-C(15)	118.1(5)
C(4)-N(2)-Ir(1)	117.3(4)
C(15)-N(2)-Ir(1)	124.6(4)
N(1)-C(3)-C(4)	114.5(6)
N(1)-C(3)-C(5)	123.6(6)
C(4)-C(3)-C(5)	121.8(6)
N(2)-C(4)-C(3)	113.8(6)
N(2)-C(4)-C(6)	123.1(6)
C(3)-C(4)-C(6)	123.1(6)
C(3)-C(5)-H(5A)	109.5
C(3)-C(5)-H(5B)	109.5
H(5A)-C(5)-H(5B)	109.5
C(3)-C(5)-H(5C)	109.5
H(5A)-C(5)-H(5C)	109.5
H(5B)-C(5)-H(5C)	109.5
C(4)-C(6)-H(6A)	109.5
C(4)-C(6)-H(6B)	109.5
H(6A)-C(6)-H(6B)	109.5
C(4)-C(6)-H(6C)	109.5
H(6A)-C(6)-H(6C)	109.5

H(6B)-C(6)-H(6C)	109.5	C(20)-C(15)-C(16)	122.6(5)
C(8)-C(7)-C(12)	121.4(5)	C(20)-C(15)-N(2)	118.7(5)
C(8)-C(7)-N(1)	119.4(5)	C(16)-C(15)-N(2)	118.7(5)
C(12)-C(7)-N(1)	119.1(5)	C(17)-C(16)-C(15)	117.7(6)
C(9)-C(8)-C(7)	118.5(5)	C(17)-C(16)-C(21)	121.4(5)
C(9)-C(8)-C(13)	121.7(5)	C(15)-C(16)-C(21)	120.9(6)
C(7)-C(8)-C(13)	119.7(5)	C(18)-C(17)-C(16)	120.9(6)
C(8)-C(9)-C(10)	120.9(5)	C(18)-C(17)-H(17A)	119.6
C(8)-C(9)-H(9A)	119.5	C(16)-C(17)-H(17A)	119.6
C(10)-C(9)-H(9A)	119.5	C(17)-C(18)-C(19)	120.2(6)
C(9)-C(10)-C(11)	120.0(6)	C(17)-C(18)-H(18A)	119.9
C(9)-C(10)-H(10A)	120.0	C(19)-C(18)-H(18A)	119.9
C(11)-C(10)-H(10A)	120.0	C(20)-C(19)-C(18)	121.0(7)
C(10)-C(11)-C(12)	121.0(6)	C(20)-C(19)-H(19A)	119.5
C(10)-C(11)-H(11A)	119.5	C(18)-C(19)-H(19A)	119.5
C(12)-C(11)-H(11A)	119.5	C(19)-C(20)-C(15)	117.7(6)
C(11)-C(12)-C(7)	118.0(5)	C(19)-C(20)-C(22)	121.7(6)
C(11)-C(12)-C(14)	121.3(5)	C(15)-C(20)-C(22)	120.6(6)
C(7)-C(12)-C(14)	120.7(5)	C(16)-C(21)-H(21A)	109.5
C(8)-C(13)-H(13A)	109.5	C(16)-C(21)-H(21B)	109.5
C(8)-C(13)-H(13B)	109.5	H(21A)-C(21)-H(21B)	109.5
H(13A)-C(13)-H(13B)	109.5	C(16)-C(21)-H(21C)	109.5
C(8)-C(13)-H(13C)	109.5	H(21A)-C(21)-H(21C)	109.5
H(13A)-C(13)-H(13C)	109.5	H(21B)-C(21)-H(21C)	109.5
H(13B)-C(13)-H(13C)	109.5	C(20)-C(22)-H(22A)	109.5
C(12)-C(14)-H(14A)	109.5	C(20)-C(22)-H(22B)	109.5
C(12)-C(14)-H(14B)	109.5	H(22A)-C(22)-H(22B)	109.5
H(14A)-C(14)-H(14B)	109.5	C(20)-C(22)-H(22C)	109.5
C(12)-C(14)-H(14C)	109.5	H(22A)-C(22)-H(22C)	109.5
H(14A)-C(14)-H(14C)	109.5	H(22B)-C(22)-H(22C)	109.5
H(14B)-C(14)-H(14C)	109.5		

Table S-4. Anisotropic displacement parameters ($Å^2$x 10^3) for **1**. The anisotropic displacement factor exponent takes the form: $-2\pi^2[h^2 a^{*2}U_{11} + ... + 2 h k a^* b^* U_{12}]$

	U_{11}	U_{22}	U_{33}	U_{23}	U_{13}	U_{12}
Ir1	20(1)	22(1)	25(1)	-2(1)	7(1)	-4(1)
Cl1	42(3)	22(2)	32(2)	0(2)	11(2)	-6(2)
C1	34(9)	51(9)	27(7)	-11(6)	7(6)	-30(8)
C2	27(9)	25(7)	31(10)	-6(7)	9(6)	-4(6)
Cl1'	42(5)	36(2)	27(2)	-8(2)	8(2)	-11(2)
C1'	63(14)	30(9)	42(10)	-7(7)	8(9)	-9(8)
C2'	46(13)	37(12)	33(9)	0(8)	5(8)	-21(9)
N1	16(2)	22(2)	22(2)	0(2)	2(2)	-2(2)
N2	17(2)	28(3)	29(3)	-1(2)	7(2)	0(2)
C3	27(3)	29(3)	35(3)	-2(3)	10(2)	0(3)
C4	30(3)	32(3)	31(3)	-1(3)	11(3)	-3(3)
C5	58(5)	34(4)	41(4)	-9(3)	20(4)	-10(3)
C6	67(5)	23(3)	51(5)	-5(3)	28(4)	-9(3)
C7	19(2)	25(3)	22(3)	1(2)	6(2)	-1(2)
C8	18(2)	31(3)	30(3)	4(2)	6(2)	3(2)
C9	22(3)	34(4)	35(3)	5(3)	13(2)	1(2)
C10	29(3)	33(3)	31(3)	7(3)	11(3)	2(3)
C11	25(3)	33(3)	25(3)	3(2)	3(2)	2(2)
C12	21(2)	26(3)	26(3)	-2(2)	3(2)	1(2)
C13	20(3)	47(4)	39(4)	10(3)	5(3)	3(3)
C14	21(3)	47(4)	35(3)	1(3)	6(2)	3(3)
C15	23(2)	29(3)	28(3)	0(3)	9(2)	-5(2)
C16	19(2)	33(3)	32(3)	3(3)	8(2)	-2(2)
C17	26(3)	33(4)	41(4)	5(3)	16(3)	2(2)
C18	38(4)	35(4)	42(4)	10(3)	20(3)	7(3)
C19	34(3)	47(4)	35(4)	14(3)	11(3)	11(3)
C20	26(3)	30(3)	36(3)	7(3)	12(3)	8(2)
C21	19(3)	60(5)	42(4)	7(4)	3(3)	-4(3)
C22	26(3)	64(5)	43(4)	17(4)	10(3)	15(3)

Table S-5. Hydrogen coordinates (x 10^4) and isotropic displacement parameters (Å^2 x 10^3) for **1**.

	x	y	z	U(eq)
H1A	8710	792	9230	44
H1B	9346	1886	8950	44
H2A	6679	2337	8563	33
H2B	6045	1246	8842	33
H1C	7449	2351	6807	54
H1D	7359	2879	7579	54
H2C	9938	2418	7946	46
H2D	10027	1890	7176	46
H5A	7588	-1159	5851	65
H5B	6190	-1817	6130	65
H5C	8084	-2114	6348	65
H6A	6498	-2430	8158	68
H6B	7468	-2769	7538	68
H6C	5552	-2503	7370	68
H9A	11412	1029	5479	35
H10A	9379	1769	4661	36
H11A	6642	1755	4846	33
H13A	12297	201	6573	52
H13B	11045	-697	6663	52
H13C	11106	282	7153	52
H14A	4644	1542	5651	51
H14B	5194	1023	6399	51
H14C	4728	337	5714	51
H17A	3713	-1028	9621	39
H18A	5817	-1594	10475	44
H19A	8524	-1647	10247	46
H21A	4158	272	8117	61
H21B	2802	-231	8520	61
H21C	3539	-856	7929	61
H22A	9995	-593	8797	66
H22B	9948	-1801	8813	66
H22C	10564	-1184	9518	66

Table S-6. Torsion angles [°] for **1**.

N2-Ir1-C1-C2	-93.4(10)	C1'-Ir1-N1-C7	17.1(9)
N1-Ir1-C1-C2	-161.3(10)	C1-Ir1-N1-C7	-109.3(11)
C2'-Ir1-C1-C2	107.2(13)	Cl1'-Ir1-N1-C7	137.8(14)
C1'-Ir1-C1-C2	72.6(12)	Cl1-Ir1-N1-C7	-3.8(4)
Cl1'-Ir1-C1-C2	1.6(11)	N1-Ir1-N2-C4	0.4(4)
Cl1-Ir1-C1-C2	91.7(10)	C2'-Ir1-N2-C4	-71.5(18)
N2-Ir1-C2-C1	90.5(10)	C2-Ir1-N2-C4	161.0(7)
N1-Ir1-C2-C1	161.4(11)	C1'-Ir1-N2-C4	68.4(18)
C2'-Ir1-C2-C1	-73.3(12)	C1-Ir1-N2-C4	-160.7(7)
C1'-Ir1-C2-C1	-108.1(12)	Cl1'-Ir1-N2-C4	175.4(5)
Cl1'-Ir1-C2-C1	-2.7(18)	Cl1-Ir1-N2-C4	-37(2)
Cl1-Ir1-C2-C1	-87.7(10)	N1-Ir1-N2-C15	179.3(5)
N2-Ir1-C1'-C2'	-157.7(15)	C2'-Ir1-N2-C15	107.4(17)
N1-Ir1-C1'-C2'	-92.3(13)	C2-Ir1-N2-C15	-20.2(7)
C2-Ir1-C1'-C2'	107.9(13)	C1'-Ir1-N2-C15	-112.7(17)
C1-Ir1-C1'-C2'	71.7(14)	C1-Ir1-N2-C15	18.2(7)
Cl1'-Ir1-C1'-C2'	94.1(13)	Cl1'-Ir1-N2-C15	-5.7(5)
Cl1-Ir1-C1'-C2'	5.6(13)	Cl1-Ir1-N2-C15	141.9(17)
N2-Ir1-C2'-C1'	160.0(12)	C7-N1-C3-C4	179.8(5)
N1-Ir1-C2'-C1'	91.1(12)	Ir1-N1-C3-C4	-0.2(7)
C2-Ir1-C2'-C1'	-72.5(13)	C7-N1-C3-C5	-1.8(9)
C1-Ir1-C2'-C1'	-109.0(14)	Ir1-N1-C3-C5	178.2(5)
Cl1'-Ir1-C2'-C1'	-86.1(12)	C15-N2-C4-C3	-179.6(5)
Cl1-Ir1-C2'-C1'	-7.4(17)	Ir1-N2-C4-C3	-0.6(7)
N2-Ir1-N1-C3	-0.1(4)	C15-N2-C4-C6	-2.2(9)
C2'-Ir1-N1-C3	160.4(9)	Ir1-N2-C4-C6	176.8(6)
C2-Ir1-N1-C3	-74.2(16)	N1-C3-C4-N2	0.6(8)
C1'-Ir1-N1-C3	-162.9(9)	C5-C3-C4-N2	-177.9(6)
C1-Ir1-N1-C3	70.7(11)	N1-C3-C4-C6	-176.9(6)
Cl1'-Ir1-N1-C3	-42.2(17)	C5-C3-C4-C6	4.7(11)
Cl1-Ir1-N1-C3	176.2(4)	C3-N1-C7-C8	-91.0(7)
N2-Ir1-N1-C7	179.9(4)	Ir1-N1-C7-C8	89.0(6)
C2'-Ir1-N1-C7	-19.6(9)	C3-N1-C7-C12	90.9(7)
C2-Ir1-N1-C7	105.9(16)	Ir1-N1-C7-C12	-89.1(6)

C12-C7-C8-C9	0.6(9)	C4-N2-C15-C16	-89.4(8)
N1-C7-C8-C9	-177.5(6)	Ir1-N2-C15-C16	91.8(6)
C12-C7-C8-C13	-179.9(6)	C20-C15-C16-C17	-2.2(10)
N1-C7-C8-C13	2.0(9)	N2-C15-C16-C17	179.6(6)
C7-C8-C9-C10	-1.1(10)	C20-C15-C16-C21	176.1(7)
C13-C8-C9-C10	179.3(7)	N2-C15-C16-C21	-2.1(10)
C8-C9-C10-C11	0.9(10)	C15-C16-C17-C18	0.9(10)
C9-C10-C11-C12	-0.1(10)	C21-C16-C17-C18	-177.3(7)
C10-C11-C12-C7	-0.5(9)	C16-C17-C18-C19	0.6(11)
C10-C11-C12-C14	-179.4(6)	C17-C18-C19-C20	-1.1(12)
C8-C7-C12-C11	0.2(9)	C18-C19-C20-C15	-0.1(11)
N1-C7-C12-C11	178.3(5)	C18-C19-C20-C22	179.8(7)
C8-C7-C12-C14	179.1(6)	C16-C15-C20-C19	1.7(10)
N1-C7-C12-C14	-2.8(9)	N2-C15-C20-C19	180.0(6)
C4-N2-C15-C20	92.4(7)	C16-C15-C20-C22	-178.2(7)
Ir1-N2-C15-C20	-86.5(7)	N2-C15-C20-C22	0.0(10)

REFERENCE NUMBER: jonjk17

4

CRYSTAL STRUCTURE REPORT

C_{28} H_{38} Cl Ir N_2

or

$IrCl(C_{20}H_{24}N_2)(COE)$

Report prepared for:

J. Kovach, Prof. W. Jones

April 21, 2007

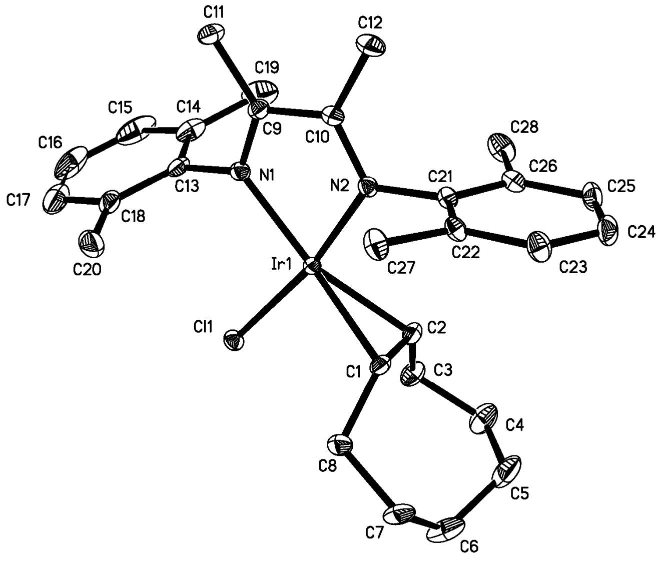

Figure S-22. X-ray Structure of IrCl(COE)(α-diimine), **4** (hydrogens omitted).

William W. Brennessel

X-ray Crystallographic Facility

Department of Chemistry

University of Rochester

120 Trustee Road

Rochester, NY 14627

Data collection

A crystal ($0.16 \times 0.14 \times 0.04$ mm^3) was placed onto the tip of a 0.1 mm diameter glass capillary tube or fiber and mounted on a Bruker SMART APEX II CCD Platform diffractometer for a data collection at 100.0(1) K.[1] A preliminary set of cell constants and an orientation matrix were calculated from 796 reflections harvested from three sets of 20 frames. These initial sets of frames were oriented such that orthogonal wedges of reciprocal space were surveyed. The data collection was carried out using MoKα radiation (graphite monochromator) with a frame time of 20 seconds and a detector distance of 5.04 cm. A randomly oriented region of reciprocal space was surveyed: four major sections of frames were collected with 0.50º steps in ω at four different ϕ settings and a detector position of -33º in 2θ. The intensity data were corrected for absorption.[2] Final cell constants were calculated from the xyz centroids of 3812 strong reflections from the actual data collection after integration.[3] See Table S-7 for additional crystal and refinement information.

Structure solution and refinement

The structure was solved using SIR97[4] and refined using SHELXL-97.[5] The space group $P2_12_12_1$ was determined based on systematic absences and intensity statistics. A direct-methods solution was calculated which provided most non-hydrogen atoms from the E-map. Full-matrix least squares / difference Fourier cycles were performed which located the remaining non-hydrogen atoms. All non-hydrogen atoms were refined with anisotropic displacement parameters. All hydrogen atoms were placed in ideal positions and refined as riding atoms with relative isotropic displacement parameters. The final full matrix least squares refinement converged to $R1 = 0.0217$ (F^2, $I > 2\sigma(I)$) and $wR2 = 0.0441$ (F^2, all data).

Structure description

The structure is the one suggested. All atoms lie on general positions.

Data collection, structure solution, and structure refinement were conducted at the X-ray Crystallographic Facility, B51 Hutchison Hall, Department of Chemistry, University of Rochester.

[1] APEX2 V2.1-0, Bruker Analytical X-ray Systems, Madison, WI (2006).

[2] SADABS 2004/1, An empirical correction for absorption anisotropy, R. Blessing, *Acta Cryst.* **A51**, 33-38 (1995).

[3] SAINT V7.34A, Bruker Analytical X-ray Systems, Madison, WI (2006).

[4] SIR97, A new tool for crystal structure determination and refinement, A. Altomare, M. C. Burla, M. Camalli, G. L. Cascarano, C. Giacovazzo, A. Guagliardi, A. G. G. Moliterni, G. Polidori, R. Spagna. *J. Appl. Cryst.* **32**, 115-119 (1999).

[5] SHELXTL V6.14, Bruker Analytical X-ray Systems, Madison, WI (2000).

Some equations of interest:

$$R_{int} = \Sigma|F_o^2 - \langle F_o^2\rangle| / \Sigma|F_o^2|$$

$$R_1 = \Sigma||F_o| - |F_c|| / \Sigma|F_o|$$

$$wR2 = [\Sigma[w(F_o^2 - F_c^2)^2] / \Sigma[w(F_o^2)^2]]^{1/2}$$

$$\text{where } w = q / [\sigma^2(F_o^2) + (a*P)^2 + b*P + d + e*\sin(\theta)]$$

$$\text{GooF} = S = [\Sigma[w(F_o^2 - F_c^2)^2] / (n-p)]^{1/2}$$

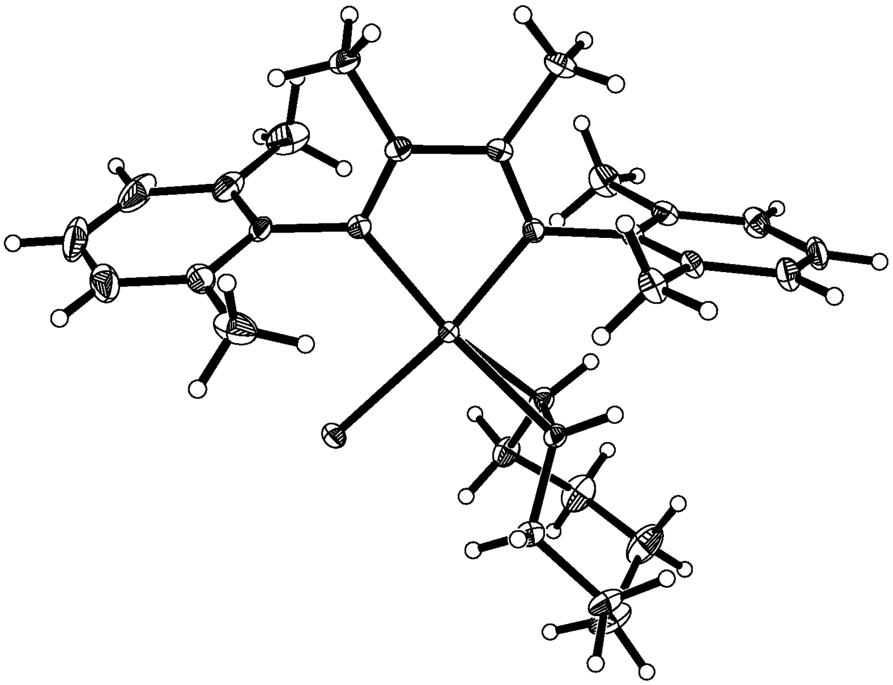

Figure S-23. X-ray Structure of IrCl(COE)(α-diimine), **4** (with hydrogens).

Table S-7. Crystal data and structure refinement for **4**.

Identification code	jonjk17	
Empirical formula	C28 H38 Cl Ir N2	
Formula weight	630.25	
Temperature	100.0(1) K	
Wavelength	0.71073 Å	
Crystal system	Orthorhombic	
Space group	$P2_12_12_1$	
Unit cell dimensions	*a* = 10.8778(6) Å	α = 90°
	b = 11.8615(7) Å	β = 90°
	c = 20.1185(11) Å	γ = 90°
Volume	2595.8(3) $Å^3$	
Z	4	
Density (calculated)	1.613 Mg/m^3	
Absorption coefficient	5.264 mm^{-1}	
F(000)	1256	
Crystal color, morphology	green-black, plate	
Crystal size	0.16 x 0.14 x 0.04 mm^3	
Theta range for data collection	1.99 to 32.57°	
Index ranges	$-16 \le h \le 16$, $-17 \le k \le 17$, $-30 \le l \le 30$	
Reflections collected	46865	
Independent reflections	9379 [*R*(int) = 0.0423]	
Observed reflections	8750	
Completeness to theta = 32.57°	99.7%	
Absorption correction	Multi-scan	
Max. and min. transmission	0.8170 and 0.4463	
Refinement method	Full-matrix least-squares on F^2	
Data / restraints / parameters	9379 / 0 / 295	
Goodness-of-fit on F^2	1.017	
Final *R* indices [*I*>2sigma(*I*)]	*R*1 = 0.0217, *wR*2 = 0.0430	
R indices (all data)	*R*1 = 0.0249, *wR*2 = 0.0441	
Absolute structure parameter	-0.017(4)	
Largest diff. peak and hole	1.252 and -0.453 e.$Å^{-3}$	

Table S-8. Atomic coordinates (x 10^4) and equivalent isotropic displacement parameters ($Å^2$x 10^3) for **4**. U_{eq} is defined as one third of the trace of the orthogonalized U_{ij} tensor.

	x	y	z	U_{eq}
Ir1	4167(1)	625(1)	1195(1)	10(1)
Cl1	6120(1)	-34(1)	961(1)	14(1)
N1	4748(2)	1585(2)	1994(1)	13(1)
N2	2648(2)	1383(2)	1475(1)	12(1)
C1	3426(2)	137(2)	232(1)	13(1)
C2	3242(2)	-741(2)	688(1)	16(1)
C3	3861(2)	-1867(2)	637(1)	19(1)
C4	3069(3)	-2686(3)	223(2)	27(1)
C5	2607(4)	-2220(3)	-443(2)	33(1)
C6	3595(3)	-1782(3)	-915(2)	34(1)
C7	3725(3)	-488(3)	-961(1)	23(1)
C8	4307(3)	76(2)	-353(1)	18(1)
C9	3912(2)	2262(2)	2248(1)	13(1)
C10	2709(2)	2152(2)	1959(1)	14(1)
C11	4153(3)	3072(2)	2802(1)	18(1)
C12	1616(3)	2813(3)	2195(2)	22(1)
C13	5942(3)	1511(2)	2304(1)	16(1)
C14	6056(3)	892(2)	2894(1)	23(1)
C15	7229(3)	796(3)	3171(2)	37(1)
C16	8243(3)	1292(3)	2872(2)	43(1)
C17	8105(3)	1906(3)	2291(2)	33(1)
C18	6948(3)	2026(2)	1998(2)	21(1)
C19	4976(3)	357(3)	3225(2)	32(1)
C20	6820(3)	2719(3)	1381(2)	28(1)
C21	1451(2)	1204(2)	1179(1)	13(1)
C22	1101(2)	1882(2)	647(1)	13(1)
C23	-15(2)	1660(2)	336(1)	18(1)
C24	-760(3)	787(2)	562(1)	20(1)
C25	-418(2)	151(2)	1107(1)	19(1)
C26	702(2)	339(2)	1427(1)	15(1)
C27	1906(2)	2843(2)	420(1)	18(1)
C28	1072(3)	-340(2)	2029(1)	23(1)

Table S-9. Bond lengths [Å] and angles [°] for **4**.

Ir(1)-N(2)	1.964(2)	C(11)-H(11A)	0.9800
Ir(1)-N(1)	2.069(2)	C(11)-H(11B)	0.9800
Ir(1)-C(2)	2.163(3)	C(11)-H(11C)	0.9800
Ir(1)-C(1)	2.177(2)	C(12)-H(12A)	0.9800
Ir(1)-Cl(1)	2.3117(6)	C(12)-H(12B)	0.9800
N(1)-C(9)	1.317(3)	C(12)-H(12C)	0.9800
N(1)-C(13)	1.443(3)	C(13)-C(18)	1.396(4)
N(2)-C(10)	1.336(3)	C(13)-C(14)	1.401(4)
N(2)-C(21)	1.448(3)	C(14)-C(15)	1.398(4)
C(1)-C(2)	1.402(4)	C(14)-C(19)	1.494(5)
C(1)-C(8)	1.518(4)	C(15)-C(16)	1.388(6)
C(1)-H(1A)	1.0000	C(15)-H(15A)	0.9500
C(2)-C(3)	1.500(4)	C(16)-C(17)	1.386(6)
C(2)-H(2A)	1.0000	C(16)-H(16A)	0.9500
C(3)-C(4)	1.541(4)	C(17)-C(18)	1.397(4)
C(3)-H(3A)	0.9900	C(17)-H(17A)	0.9500
C(3)-H(3B)	0.9900	C(18)-C(20)	1.496(4)
C(4)-C(5)	1.535(4)	C(19)-H(19A)	0.9800
C(4)-H(4A)	0.9900	C(19)-H(19B)	0.9800
C(4)-H(4B)	0.9900	C(19)-H(19C)	0.9800
C(5)-C(6)	1.525(5)	C(20)-H(20A)	0.9800
C(5)-H(5A)	0.9900	C(20)-H(20B)	0.9800
C(5)-H(5B)	0.9900	C(20)-H(20C)	0.9800
C(6)-C(7)	1.544(5)	C(21)-C(22)	1.391(3)
C(6)-H(6A)	0.9900	C(21)-C(26)	1.402(3)
C(6)-H(6B)	0.9900	C(22)-C(23)	1.390(3)
C(7)-C(8)	1.532(4)	C(22)-C(27)	1.508(4)
C(7)-H(7A)	0.9900	C(23)-C(24)	1.392(4)
C(7)-H(7B)	0.9900	C(23)-H(23A)	0.9500
C(8)-H(8A)	0.9900	C(24)-C(25)	1.381(4)
C(8)-H(8B)	0.9900	C(24)-H(24A)	0.9500
C(9)-C(10)	1.438(3)	C(25)-C(26)	1.396(4)
C(9)-C(11)	1.494(3)	C(25)-H(25A)	0.9500
C(10)-C(12)	1.501(4)	C(26)-C(28)	1.509(4)

C(27)-H(27A)	0.9800	C(2)-C(3)-H(3B)	109.6
C(27)-H(27B)	0.9800	C(4)-C(3)-H(3B)	109.6
C(27)-H(27C)	0.9800	H(3A)-C(3)-H(3B)	108.1
C(28)-H(28A)	0.9800	C(5)-C(4)-C(3)	115.3(2)
C(28)-H(28B)	0.9800	C(5)-C(4)-H(4A)	108.4
C(28)-H(28C)	0.9800	C(3)-C(4)-H(4A)	108.4
N(2)-Ir(1)-N(1)	77.40(8)	C(5)-C(4)-H(4B)	108.4
N(2)-Ir(1)-C(2)	94.97(9)	C(3)-C(4)-H(4B)	108.4
N(1)-Ir(1)-C(2)	157.02(9)	H(4A)-C(4)-H(4B)	107.5
N(2)-Ir(1)-C(1)	93.76(9)	C(6)-C(5)-C(4)	115.8(3)
N(1)-Ir(1)-C(1)	162.02(9)	C(6)-C(5)-H(5A)	108.3
C(2)-Ir(1)-C(1)	37.70(9)	C(4)-C(5)-H(5A)	108.3
N(2)-Ir(1)-Cl(1)	170.52(6)	C(6)-C(5)-H(5B)	108.3
N(1)-Ir(1)-Cl(1)	93.64(6)	C(4)-C(5)-H(5B)	108.3
C(2)-Ir(1)-Cl(1)	94.50(7)	H(5A)-C(5)-H(5B)	107.4
C(1)-Ir(1)-Cl(1)	93.99(7)	C(5)-C(6)-C(7)	116.2(3)
C(9)-N(1)-C(13)	119.4(2)	C(5)-C(6)-H(6A)	108.2
C(9)-N(1)-Ir(1)	115.27(17)	C(7)-C(6)-H(6A)	108.2
C(13)-N(1)-Ir(1)	125.26(16)	C(5)-C(6)-H(6B)	108.2
C(10)-N(2)-C(21)	116.4(2)	C(7)-C(6)-H(6B)	108.2
C(10)-N(2)-Ir(1)	118.68(17)	H(6A)-C(6)-H(6B)	107.4
C(21)-N(2)-Ir(1)	124.86(16)	C(8)-C(7)-C(6)	115.1(2)
C(2)-C(1)-C(8)	124.1(2)	C(8)-C(7)-H(7A)	108.5
C(2)-C(1)-Ir(1)	70.62(14)	C(6)-C(7)-H(7A)	108.5
C(8)-C(1)-Ir(1)	117.87(17)	C(8)-C(7)-H(7B)	108.5
C(2)-C(1)-H(1A)	112.6	C(6)-C(7)-H(7B)	108.5
C(8)-C(1)-H(1A)	112.6	H(7A)-C(7)-H(7B)	107.5
Ir(1)-C(1)-H(1A)	112.6	C(1)-C(8)-C(7)	112.3(2)
C(1)-C(2)-C(3)	123.6(2)	C(1)-C(8)-H(8A)	109.2
C(1)-C(2)-Ir(1)	71.68(15)	C(7)-C(8)-H(8A)	109.2
C(3)-C(2)-Ir(1)	119.38(17)	C(1)-C(8)-H(8B)	109.2
C(1)-C(2)-H(2A)	112.1	C(7)-C(8)-H(8B)	109.2
C(3)-C(2)-H(2A)	112.1	H(8A)-C(8)-H(8B)	107.9
Ir(1)-C(2)-H(2A)	112.1	N(1)-C(9)-C(10)	114.5(2)
C(2)-C(3)-C(4)	110.3(2)	N(1)-C(9)-C(11)	124.1(2)
C(2)-C(3)-H(3A)	109.6	C(10)-C(9)-C(11)	121.3(2)
C(4)-C(3)-H(3A)	109.6	N(2)-C(10)-C(9)	113.7(2)

N(2)-C(10)-C(12)	123.2(2)	H(19B)-C(19)-H(19C)	109.5
C(9)-C(10)-C(12)	123.1(2)	C(18)-C(20)-H(20A)	109.5
C(9)-C(11)-H(11A)	109.5	C(18)-C(20)-H(20B)	109.5
C(9)-C(11)-H(11B)	109.5	H(20A)-C(20)-H(20B)	109.5
H(11A)-C(11)-H(11B)	109.5	C(18)-C(20)-H(20C)	109.5
C(9)-C(11)-H(11C)	109.5	H(20A)-C(20)-H(20C)	109.5
H(11A)-C(11)-H(11C)	109.5	H(20B)-C(20)-H(20C)	109.5
H(11B)-C(11)-H(11C)	109.5	C(22)-C(21)-C(26)	122.6(2)
C(10)-C(12)-H(12A)	109.5	C(22)-C(21)-N(2)	118.6(2)
C(10)-C(12)-H(12B)	109.5	C(26)-C(21)-N(2)	118.9(2)
H(12A)-C(12)-H(12B)	109.5	C(23)-C(22)-C(21)	118.4(2)
C(10)-C(12)-H(12C)	109.5	C(23)-C(22)-C(27)	120.9(2)
H(12A)-C(12)-H(12C)	109.5	C(21)-C(22)-C(27)	120.7(2)
H(12B)-C(12)-H(12C)	109.5	C(22)-C(23)-C(24)	120.2(3)
C(18)-C(13)-C(14)	122.2(3)	C(22)-C(23)-H(23A)	119.9
C(18)-C(13)-N(1)	119.2(2)	C(24)-C(23)-H(23A)	119.9
C(14)-C(13)-N(1)	118.5(2)	C(25)-C(24)-C(23)	120.6(2)
C(15)-C(14)-C(13)	117.5(3)	C(25)-C(24)-H(24A)	119.7
C(15)-C(14)-C(19)	120.3(3)	C(23)-C(24)-H(24A)	119.7
C(13)-C(14)-C(19)	122.1(3)	C(24)-C(25)-C(26)	120.9(2)
C(16)-C(15)-C(14)	121.2(3)	C(24)-C(25)-H(25A)	119.5
C(16)-C(15)-H(15A)	119.4	C(26)-C(25)-H(25A)	119.5
C(14)-C(15)-H(15A)	119.4	C(25)-C(26)-C(21)	117.4(2)
C(17)-C(16)-C(15)	120.2(3)	C(25)-C(26)-C(28)	121.2(2)
C(17)-C(16)-H(16A)	119.9	C(21)-C(26)-C(28)	121.4(2)
C(15)-C(16)-H(16A)	119.9	C(22)-C(27)-H(27A)	109.5
C(16)-C(17)-C(18)	120.5(3)	C(22)-C(27)-H(27B)	109.5
C(16)-C(17)-H(17A)	119.8	H(27A)-C(27)-H(27B)	109.5
C(18)-C(17)-H(17A)	119.8	C(22)-C(27)-H(27C)	109.5
C(13)-C(18)-C(17)	118.4(3)	H(27A)-C(27)-H(27C)	109.5
C(13)-C(18)-C(20)	122.2(3)	H(27B)-C(27)-H(27C)	109.5
C(17)-C(18)-C(20)	119.3(3)	C(26)-C(28)-H(28A)	109.5
C(14)-C(19)-H(19A)	109.5	C(26)-C(28)-H(28B)	109.5
C(14)-C(19)-H(19B)	109.5	H(28A)-C(28)-H(28B)	109.5
H(19A)-C(19)-H(19B)	109.5	C(26)-C(28)-H(28C)	109.5
C(14)-C(19)-H(19C)	109.5	H(28A)-C(28)-H(28C)	109.5
H(19A)-C(19)-H(19C)	109.5	H(28B)-C(28)-H(28C)	109.5

Table S-10. Anisotropic displacement parameters ($Å^2 x 10^3$) for **4**. The anisotropic displacement factor exponent takes the form: $-2\pi^2[h^2 a^{*2}U_{11} + ... + 2 h k a^* b^* U_{12}]$

	U_{11}	U_{22}	U_{33}	U_{23}	U_{13}	U_{12}
Ir1	9(1)	11(1)	10(1)	-1(1)	0(1)	0(1)
Cl1	11(1)	14(1)	16(1)	0(1)	1(1)	1(1)
N1	12(1)	15(1)	11(1)	0(1)	0(1)	0(1)
N2	10(1)	14(1)	12(1)	1(1)	0(1)	0(1)
C1	13(1)	13(1)	14(1)	-5(1)	-2(1)	1(1)
C2	16(1)	16(1)	15(1)	-3(1)	-2(1)	-4(1)
C3	24(1)	14(1)	20(1)	-1(1)	-6(1)	0(1)
C4	37(2)	15(1)	30(2)	-1(1)	-7(1)	-6(1)
C5	44(2)	27(2)	28(2)	-5(1)	-11(2)	-14(2)
C6	52(2)	29(2)	20(2)	-10(1)	-4(1)	1(2)
C7	32(1)	27(2)	10(1)	-1(1)	-1(1)	0(1)
C8	19(1)	22(1)	12(1)	0(1)	1(1)	0(1)
C9	16(1)	12(1)	12(1)	0(1)	1(1)	-1(1)
C10	13(1)	16(1)	14(1)	-2(1)	2(1)	1(1)
C11	22(1)	17(1)	16(1)	-6(1)	-1(1)	1(1)
C12	17(1)	26(2)	23(2)	-7(1)	5(1)	5(1)
C13	14(1)	17(1)	16(1)	-8(1)	-5(1)	2(1)
C14	29(2)	20(1)	20(1)	-5(1)	-8(1)	6(1)
C15	49(2)	34(2)	29(2)	-12(1)	-24(2)	20(2)
C16	24(2)	44(2)	60(3)	-30(2)	-24(2)	14(2)
C17	18(1)	32(2)	51(2)	-22(2)	-4(1)	2(1)
C18	16(1)	18(1)	29(2)	-11(1)	1(1)	-1(1)
C19	49(2)	25(2)	20(2)	4(1)	0(1)	6(1)
C20	29(2)	21(1)	35(2)	-7(1)	14(1)	-8(1)
C21	10(1)	16(1)	14(1)	-1(1)	1(1)	1(1)
C22	10(1)	15(1)	16(1)	3(1)	1(1)	2(1)
C23	12(1)	21(1)	21(1)	4(1)	-2(1)	2(1)
C24	10(1)	23(1)	26(1)	-2(1)	-4(1)	1(1)
C25	13(1)	18(1)	25(2)	0(1)	2(1)	-5(1)
C26	14(1)	16(1)	16(1)	1(1)	3(1)	1(1)
C27	14(1)	15(1)	24(1)	5(1)	1(1)	0(1)
C28	22(2)	24(1)	22(1)	10(1)	4(1)	0(1)

Table S-11. Hydrogen coordinates (x 10^4) and isotropic displacement parameters ($Å^2$ x 10^3) for **4**.

	x	y	z	U(eq)
H1A	2647	545	120	16
H2A	2361	-809	824	19
H3A	3988	-2182	1087	23
H3B	4676	-1777	424	23
H4A	3556	-3374	135	33
H4B	2348	-2910	493	33
H5A	2144	-2823	-673	40
H5B	2024	-1599	-351	40
H6A	3419	-2076	-1365	40
H6B	4396	-2098	-774	40
H7A	4230	-305	-1356	27
H7B	2899	-159	-1032	27
H8A	5047	-353	-219	21
H8B	4570	849	-473	21
H11A	5042	3155	2864	27
H11B	3782	2786	3213	27
H11C	3793	3806	2692	27
H12A	1183	3131	1811	33
H12B	1891	3425	2486	33
H12C	1060	2314	2440	33
H15A	7334	383	3572	45
H16A	9034	1211	3066	51
H17A	8802	2247	2090	40
H19A	4413	61	2887	47
H19B	4547	920	3496	47
H19C	5258	-261	3510	47
H20A	6099	2465	1130	42
H20B	7559	2631	1106	42
H20C	6719	3514	1501	42
H23A	-269	2106	-31	22

H24A	-1511	627	340	23
H25A	-953	-422	1266	22
H27A	2721	2553	302	26
H27B	1986	3396	780	26
H27C	1535	3205	31	26
H28A	1888	-669	1956	34
H28B	473	-945	2102	34
H28C	1097	152	2420	34

Table S-12. Torsion angles [°] for **4**.

N2-Ir1-N1-C9	-5.35(18)	C1-C2-C3-C4	-90.2(3)
C2-Ir1-N1-C9	-77.9(3)	Ir1-C2-C3-C4	-176.80(19)
C1-Ir1-N1-C9	56.6(4)	C2-C3-C4-C5	49.0(4)
Cl1-Ir1-N1-C9	171.51(18)	C3-C4-C5-C6	55.6(4)
N2-Ir1-N1-C13	170.8(2)	C4-C5-C6-C7	-103.5(3)
C2-Ir1-N1-C13	98.2(3)	C5-C6-C7-C8	72.5(4)
C1-Ir1-N1-C13	-127.3(3)	C2-C1-C8-C7	84.5(3)
Cl1-Ir1-N1-C13	-12.3(2)	Ir1-C1-C8-C7	169.06(18)
N1-Ir1-N2-C10	5.22(18)	C6-C7-C8-C1	-74.9(3)
C2-Ir1-N2-C10	163.26(19)	C13-N1-C9-C10	-171.7(2)
C1-Ir1-N2-C10	-158.94(19)	Ir1-N1-C9-C10	4.7(3)
Cl1-Ir1-N2-C10	-14.1(5)	C13-N1-C9-C11	7.1(4)
N1-Ir1-N2-C21	-177.3(2)	Ir1-N1-C9-C11	-176.56(19)
C2-Ir1-N2-C21	-19.3(2)	C21-N2-C10-C9	178.0(2)
C1-Ir1-N2-C21	18.5(2)	Ir1-N2-C10-C9	-4.3(3)
Cl1-Ir1-N2-C21	163.3(3)	C21-N2-C10-C12	-0.1(4)
N2-Ir1-C1-C2	-93.26(15)	Ir1-N2-C10-C12	177.6(2)
N1-Ir1-C1-C2	-152.9(3)	N1-C9-C10-N2	-0.5(3)
Cl1-Ir1-C1-C2	92.21(14)	C11-C9-C10-N2	-179.3(2)
N2-Ir1-C1-C8	147.62(19)	N1-C9-C10-C12	177.6(2)
N1-Ir1-C1-C8	88.0(3)	C11-C9-C10-C12	-1.2(4)
C2-Ir1-C1-C8	-119.1(3)	C9-N1-C13-C18	-105.7(3)
Cl1-Ir1-C1-C8	-26.91(19)	Ir1-N1-C13-C18	78.3(3)
C8-C1-C2-C3	-2.5(4)	C9-N1-C13-C14	76.2(3)
Ir1-C1-C2-C3	-113.6(2)	Ir1-N1-C13-C14	-99.8(2)
C8-C1-C2-Ir1	111.1(2)	C18-C13-C14-C15	-0.4(4)
N2-Ir1-C2-C1	89.73(15)	N1-C13-C14-C15	177.6(2)
N1-Ir1-C2-C1	158.9(2)	C18-C13-C14-C19	179.0(3)
Cl1-Ir1-C2-C1	-90.70(14)	N1-C13-C14-C19	-2.9(4)
N2-Ir1-C2-C3	-151.5(2)	C13-C14-C15-C16	-0.2(4)
N1-Ir1-C2-C3	-82.3(3)	C19-C14-C15-C16	-179.7(3)
C1-Ir1-C2-C3	118.8(3)	C14-C15-C16-C17	0.6(5)
Cl1-Ir1-C2-C3	28.1(2)	C15-C16-C17-C18	-0.4(5)

C14-C13-C18-C17	0.7(4)	C26-C21-C22-C27	176.7(2)
N1-C13-C18-C17	-177.4(2)	N2-C21-C22-C27	-4.9(4)
C14-C13-C18-C20	-177.9(3)	C21-C22-C23-C24	0.8(4)
N1-C13-C18-C20	4.1(4)	C27-C22-C23-C24	-178.5(2)
C16-C17-C18-C13	-0.2(5)	C22-C23-C24-C25	1.7(4)
C16-C17-C18-C20	178.4(3)	C23-C24-C25-C26	-2.5(4)
C10-N2-C21-C22	87.5(3)	C24-C25-C26-C21	0.8(4)
Ir1-N2-C21-C22	-90.0(2)	C24-C25-C26-C28	179.0(3)
C10-N2-C21-C26	-94.0(3)	C22-C21-C26-C25	1.8(4)
Ir1-N2-C21-C26	88.5(3)	N2-C21-C26-C25	-176.6(2)
C26-C21-C22-C23	-2.6(4)	C22-C21-C26-C28	-176.5(2)
N2-C21-C22-C23	175.9(2)	N2-C21-C26-C28	5.1(4)

REFERENCE NUMBER: jonjk04

6

CRYSTAL STRUCTURE REPORT

C_{42} H_{52} Cl_{6} N_{4} Rh_{2}

or

$[RhCl(N_2C_{20}H_{24})]_2 \cdot 2CH_2Cl_2$

Report prepared for:

J. Kovach, Prof. W. Jones

September 28, 2006

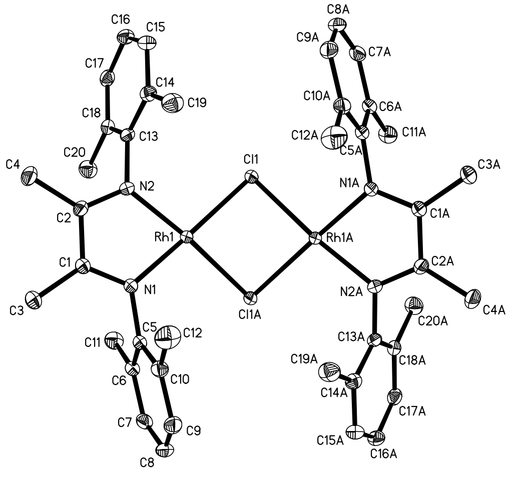

Figure S-24. X-ray Structure of $[RhCl(\alpha\text{-diimine})]_2$, **6** (hydrogens omitted).

William W. Brennessel

X-ray Crystallographic Facility

Department of Chemistry

University of Rochester

120 Trustee Road

Rochester, NY 14627

Data collection

A crystal (0.15 x 0.12 x 0.05 mm^3) was placed onto the tip of a 0.1 mm diameter glass capillary tube or fiber and mounted on a Bruker SMART APEX II CCD Platform diffractometer for a data collection at 100.0(1) K.[1] A preliminary set of cell constants and an orientation matrix were calculated from 304 reflections harvested from three sets of 20 frames. These initial sets of frames were oriented such that orthogonal wedges of reciprocal space were surveyed. The data collection was carried out using MoKα radiation (graphite monochromator) with a frame time of 30 seconds and a detector distance of 5.04 cm. A randomly oriented region of reciprocal space was surveyed: four major sections of frames were collected with 0.50º steps in ω at four different ϕ settings and a detector position of -33º in 2θ. The intensity data were corrected for absorption.[2] Final cell constants were calculated from the xyz centroids of 3615 strong reflections from the actual data collection after integration.[3] See Table S-13 for additional crystal and refinement information.

Structure solution and refinement

The structure was solved using SIR97[4] and refined using SHELXL-97.[5] The space group $C2/c$ was determined based on systematic absences and intensity statistics. A direct-methods solution was calculated which provided most non-hydrogen atoms from the E-map. Full-matrix least squares / difference Fourier cycles were performed which located the remaining non-hydrogen atoms. All non-hydrogen atoms were refined with anisotropic displacement parameters. All hydrogen atoms were placed in ideal positions and refined as riding atoms with relative isotropic displacement parameters. The final full matrix least squares refinement converged to $R1 = 0.0288$ (F^2, $I > 2\sigma(I)$) and $wR2 = 0.0626$ (F^2, all data).

Structure description

The structure is the one suggested. The molecule lies on a crystallographic two-fold axis; thus, one half of the molecule and one dichloromethane solvent molecule are unique. There are two dichloromethane solvent molecules per dirhodium molecule. The deviation from planar geometry, or twist angle, about each Rh center is 7.02(5)°. The angle between Rh planes that share an edge defined by the Cl atoms is 42.35(2)°.

Data collection, structure solution, and structure refinement were conducted at the X-ray Crystallographic Facility, B51 Hutchison Hall, Department of Chemistry, University of Rochester.

[1] APEX2 V2.0-2, Bruker Analytical X-ray Systems, Madison, WI (2005).

[2] SADABS 2004/1, An empirical correction for absorption anisotropy, R. Blessing, *Acta Cryst.* **A51**, 33-38 (1995).

[3] SAINT V7.23A, Bruker Analytical X-ray Systems, Madison, WI (2005).

[4] SIR97, A new tool for crystal structure determination and refinement, A. Altomare, M. C. Burla, M. Camalli, G. Cascarano, C. Giacovazzo, A. Guagliardi, A. G. G. Moliterni, G. Polidori, R. Spagna. *J. Appl. Cryst.* **32**, 115-119 (1998).

[5] SHELXTL V6.14, Bruker Analytical X-ray Systems, Madison, WI (2000).

Some equations of interest:

$$R_{int} = \Sigma|F_o^2 - \langle F_o^2 \rangle| / \Sigma|F_o^2|$$
$$R_1 = \Sigma||F_o|-|F_c|| / \Sigma|F_o|$$
$$wR2 = [\Sigma[w(F_o^2-F_c^2)^2] / \Sigma[w(F_o^2)^2]]^{1/2}$$
$$\text{where } w = q / [\sigma^2(F_o^2) + (a{*}P)^2 + b{*}P + d + e{*}\sin(\theta)]$$
$$\text{GooF} = S = [\Sigma[w(F_o^2-F_c^2)^2] / (n-p)]^{1/2}$$

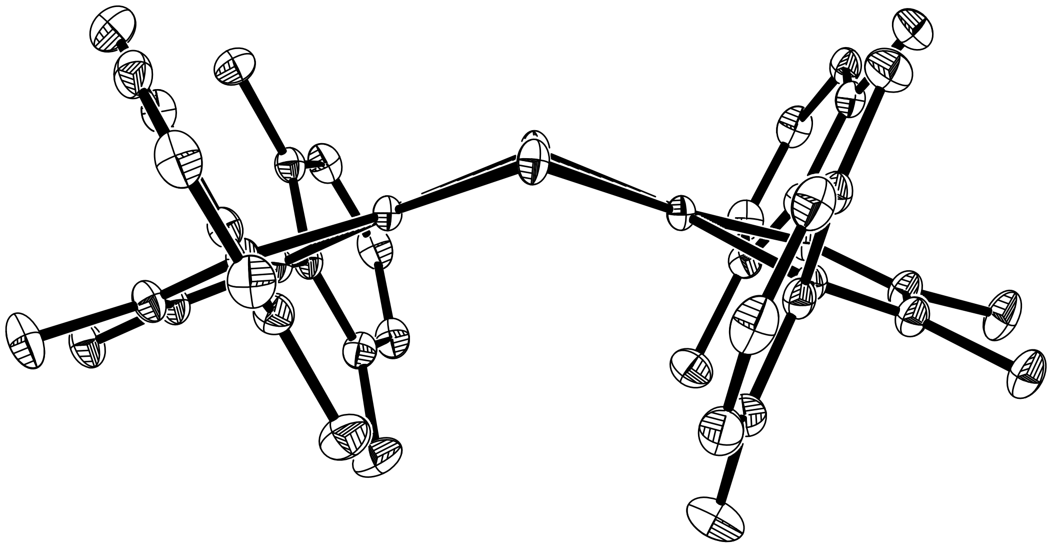

Figure S-25. X-ray Structure of [RhCl(α-diimine)]$_2$, **6** (hydrogens omitted).

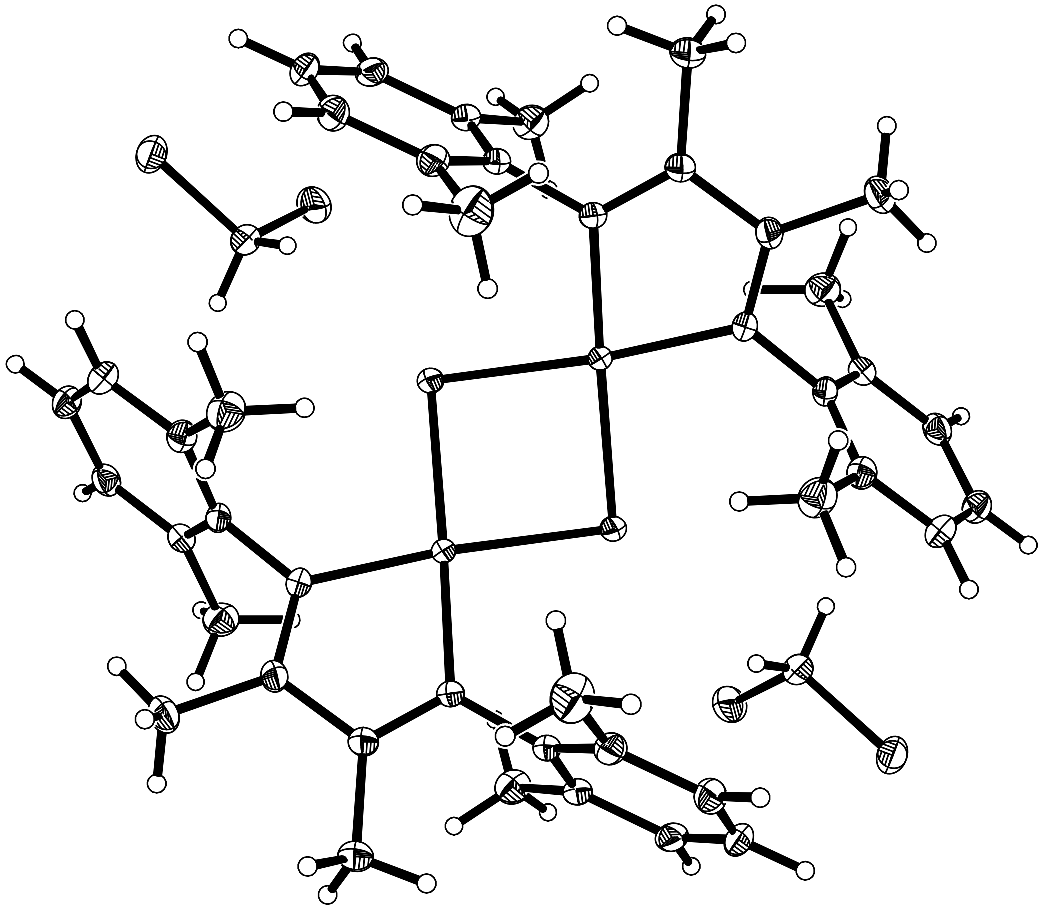

Figure S-26. X-ray Structure of $[RhCl(\alpha\text{-diimine})]_2$, **6** (with hydrogens).

Table S-13. Crystal data and structure refinement for **6**.

Identification code	jonjk04	
Empirical formula	C42 H52 Cl6 N4 Rh2	
Formula weight	1031.40	
Temperature	100.0(1) K	
Wavelength	0.71073 Å	
Crystal system	Monoclinic	
Space group	*C*2/*c*	
Unit cell dimensions	*a* = 23.823(4) Å	α = 90°
	b = 13.255(2) Å	β = 92.900(2)°
	c = 14.118(2) Å	γ = 90°
Volume	4452.6(13) $Å^3$	
Z	4	
Density (calculated)	1.539 Mg/m^3	
Absorption coefficient	1.136 mm^{-1}	
F(000)	2096	
Crystal color, morphology	red, plate	
Crystal size	0.15 x 0.12 x 0.05 mm^3	
Theta range for data collection	1.71 to 32.58°	
Index ranges	$-35 \le h \le 35, -20 \le k \le 20, -21 \le l \le 21$	
Reflections collected	39437	
Independent reflections	8031 [*R*(int) = 0.0456]	
Observed reflections	6389	
Completeness to theta = 32.58°	99.0%	
Absorption correction	Multi-scan	
Max. and min. transmission	0.9454 and 0.8481	
Refinement method	Full-matrix least-squares on F^2	
Data / restraints / parameters	8031 / 0 / 250	
Goodness-of-fit on F^2	1.017	
Final *R* indices [*I*>2sigma(*I*)]	*R*1 = 0.0288, *wR*2 = 0.0574	
R indices (all data)	*R*1 = 0.0439, *wR*2 = 0.0626	
Largest diff. peak and hole	0.499 and -0.710 e.$Å^{-3}$	

Table S-14. Atomic coordinates (x 10^4) and equivalent isotropic displacement parameters ($Å^2$x 10^3) for **6**. U_{eq} is defined as one third of the trace of the orthogonalized U_{ij} tensor.

	x	y	z	U_{eq}
Rh1	5364(1)	2292(1)	3522(1)	12(1)
Cl1	4435(1)	1811(1)	3043(1)	16(1)
N1	6108(1)	2804(1)	3937(1)	14(1)
N2	5264(1)	2601(1)	4867(1)	15(1)
C1	6185(1)	3122(1)	4822(1)	17(1)
C2	5696(1)	2994(1)	5364(1)	18(1)
C3	6731(1)	3529(2)	5238(1)	23(1)
C4	5689(1)	3264(2)	6396(1)	26(1)
C5	6558(1)	2845(1)	3300(1)	15(1)
C6	6895(1)	1991(1)	3211(1)	16(1)
C7	7308(1)	2017(2)	2544(1)	21(1)
C8	7374(1)	2860(2)	1979(1)	23(1)
C9	7035(1)	3697(2)	2084(1)	23(1)
C10	6624(1)	3709(1)	2752(1)	19(1)
C11	6805(1)	1070(1)	3803(1)	23(1)
C12	6261(1)	4619(2)	2884(2)	31(1)
C13	4779(1)	2292(1)	5364(1)	15(1)
C14	4299(1)	2899(1)	5333(1)	18(1)
C15	3844(1)	2574(2)	5842(1)	21(1)
C16	3871(1)	1679(2)	6357(1)	22(1)
C17	4349(1)	1084(1)	6363(1)	20(1)
C18	4812(1)	1377(1)	5861(1)	17(1)
C19	4277(1)	3876(2)	4787(1)	25(1)
C20	5336(1)	742(2)	5874(1)	24(1)
C21	6731(1)	600(2)	757(1)	23(1)
Cl2	7360(1)	982(1)	237(1)	26(1)
Cl3	6857(1)	-474(1)	1482(1)	32(1)

Table S-15. Bond lengths [Å] and angles [°] for **6**.

Rh(1)-N(1)	1.9591(14)	C(12)-H(12C)	0.9800
Rh(1)-N(2)	1.9684(14)	C(13)-C(14)	1.397(2)
Rh(1)-Cl(1)	2.3678(5)	C(13)-C(18)	1.401(2)
Rh(1)-Cl(1)#1	2.3715(5)	C(14)-C(15)	1.398(2)
Cl(1)-Rh(1)#1	2.3715(5)	C(14)-C(19)	1.506(3)
N(1)-C(1)	1.323(2)	C(15)-C(16)	1.392(3)
N(1)-C(5)	1.436(2)	C(15)-H(15A)	0.9500
N(2)-C(2)	1.323(2)	C(16)-C(17)	1.386(3)
N(2)-C(13)	1.442(2)	C(16)-H(16A)	0.9500
C(1)-C(2)	1.437(2)	C(17)-C(18)	1.395(2)
C(1)-C(3)	1.500(2)	C(17)-H(17A)	0.9500
C(2)-C(4)	1.501(2)	C(18)-C(20)	1.505(2)
C(3)-H(3A)	0.9800	C(19)-H(19A)	0.9800
C(3)-H(3B)	0.9800	C(19)-H(19B)	0.9800
C(3)-H(3C)	0.9800	C(19)-H(19C)	0.9800
C(4)-H(4A)	0.9800	C(20)-H(20A)	0.9800
C(4)-H(4B)	0.9800	C(20)-H(20B)	0.9800
C(4)-H(4C)	0.9800	C(20)-H(20C)	0.9800
C(5)-C(10)	1.394(2)	C(21)-Cl(3)	1.770(2)
C(5)-C(6)	1.397(2)	C(21)-Cl(2)	1.7750(19)
C(6)-C(7)	1.398(2)	C(21)-H(21A)	0.9900
C(6)-C(11)	1.501(3)	C(21)-H(21B)	0.9900
C(7)-C(8)	1.386(3)	N(1)-Rh(1)-N(2)	77.96(6)
C(7)-H(7A)	0.9500	N(1)-Rh(1)-Cl(1)	175.19(4)
C(8)-C(9)	1.384(3)	N(2)-Rh(1)-Cl(1)	100.06(4)
C(8)-H(8A)	0.9500	N(1)-Rh(1)-Cl(1)#1	98.56(4)
C(9)-C(10)	1.395(2)	N(2)-Rh(1)-Cl(1)#1	173.97(4)
C(9)-H(9A)	0.9500	Cl(1)-Rh(1)-Cl(1)#1	83.785(17)
C(10)-C(12)	1.502(3)	Rh(1)-Cl(1)-Rh(1)#1	87.917(15)
C(11)-H(11A)	0.9800	C(1)-N(1)-C(5)	120.39(14)
C(11)-H(11B)	0.9800	C(1)-N(1)-Rh(1)	118.28(11)
C(11)-H(11C)	0.9800	C(5)-N(1)-Rh(1)	121.33(11)
C(12)-H(12A)	0.9800	C(2)-N(2)-C(13)	118.30(14)
C(12)-H(12B)	0.9800	C(2)-N(2)-Rh(1)	117.71(11)

C(13)-N(2)-Rh(1)	123.37(11)	C(9)-C(10)-C(12)	121.47(17)
N(1)-C(1)-C(2)	112.77(15)	C(6)-C(11)-H(11A)	109.5
N(1)-C(1)-C(3)	124.20(15)	C(6)-C(11)-H(11B)	109.5
C(2)-C(1)-C(3)	122.97(15)	H(11A)-C(11)-H(11B)	109.5
N(2)-C(2)-C(1)	113.21(15)	C(6)-C(11)-H(11C)	109.5
N(2)-C(2)-C(4)	124.22(16)	H(11A)-C(11)-H(11C)	109.5
C(1)-C(2)-C(4)	122.55(16)	H(11B)-C(11)-H(11C)	109.5
C(1)-C(3)-H(3A)	109.5	C(10)-C(12)-H(12A)	109.5
C(1)-C(3)-H(3B)	109.5	C(10)-C(12)-H(12B)	109.5
H(3A)-C(3)-H(3B)	109.5	H(12A)-C(12)-H(12B)	109.5
C(1)-C(3)-H(3C)	109.5	C(10)-C(12)-H(12C)	109.5
H(3A)-C(3)-H(3C)	109.5	H(12A)-C(12)-H(12C)	109.5
H(3B)-C(3)-H(3C)	109.5	H(12B)-C(12)-H(12C)	109.5
C(2)-C(4)-H(4A)	109.5	C(14)-C(13)-C(18)	122.66(15)
C(2)-C(4)-H(4B)	109.5	C(14)-C(13)-N(2)	119.71(15)
H(4A)-C(4)-H(4B)	109.5	C(18)-C(13)-N(2)	117.63(15)
C(2)-C(4)-H(4C)	109.5	C(13)-C(14)-C(15)	117.43(16)
H(4A)-C(4)-H(4C)	109.5	C(13)-C(14)-C(19)	121.21(16)
H(4B)-C(4)-H(4C)	109.5	C(15)-C(14)-C(19)	121.34(16)
C(10)-C(5)-C(6)	122.36(16)	C(16)-C(15)-C(14)	120.98(17)
C(10)-C(5)-N(1)	119.14(15)	C(16)-C(15)-H(15A)	119.5
C(6)-C(5)-N(1)	118.38(15)	C(14)-C(15)-H(15A)	119.5
C(5)-C(6)-C(7)	117.84(16)	C(17)-C(16)-C(15)	120.33(16)
C(5)-C(6)-C(11)	120.80(15)	C(17)-C(16)-H(16A)	119.8
C(7)-C(6)-C(11)	121.34(16)	C(15)-C(16)-H(16A)	119.8
C(8)-C(7)-C(6)	120.80(17)	C(16)-C(17)-C(18)	120.57(17)
C(8)-C(7)-H(7A)	119.6	C(16)-C(17)-H(17A)	119.7
C(6)-C(7)-H(7A)	119.6	C(18)-C(17)-H(17A)	119.7
C(9)-C(8)-C(7)	120.10(17)	C(17)-C(18)-C(13)	118.00(16)
C(9)-C(8)-H(8A)	119.9	C(17)-C(18)-C(20)	120.91(16)
C(7)-C(8)-H(8A)	119.9	C(13)-C(18)-C(20)	121.07(15)
C(8)-C(9)-C(10)	120.97(17)	C(14)-C(19)-H(19A)	109.5
C(8)-C(9)-H(9A)	119.5	C(14)-C(19)-H(19B)	109.5
C(10)-C(9)-H(9A)	119.5	H(19A)-C(19)-H(19B)	109.5
C(5)-C(10)-C(9)	117.91(17)	C(14)-C(19)-H(19C)	109.5
C(5)-C(10)-C(12)	120.62(16)	H(19A)-C(19)-H(19C)	109.5

H(19B)-C(19)-H(19C)	109.5	Cl(3)-C(21)-Cl(2)	110.26(10)
C(18)-C(20)-H(20A)	109.5	Cl(3)-C(21)-H(21A)	109.6
C(18)-C(20)-H(20B)	109.5	Cl(2)-C(21)-H(21A)	109.6
H(20A)-C(20)-H(20B)	109.5	Cl(3)-C(21)-H(21B)	109.6
C(18)-C(20)-H(20C)	109.5	Cl(2)-C(21)-H(21B)	109.6
H(20A)-C(20)-H(20C)	109.5	H(21A)-C(21)-H(21B)	108.1
H(20B)-C(20)-H(20C)	109.5		

Symmetry transformations used to generate equivalent atoms:

#1 -x+1,y,-z+1/2

Table S-16. Anisotropic displacement parameters ($Å^2$x 10^3) for **6**. The anisotropic displacement factor exponent takes the form: $-2\pi^2[h^2 a^{*2}U_{11} + ... + 2 h k a^* b^* U_{12}]$

	U_{11}	U_{22}	U_{33}	U_{23}	U_{13}	U_{12}
Rh1	10(1)	16(1)	11(1)	0(1)	1(1)	0(1)
Cl1	12(1)	24(1)	13(1)	2(1)	0(1)	-2(1)
N1	12(1)	17(1)	14(1)	1(1)	0(1)	-1(1)
N2	14(1)	19(1)	13(1)	0(1)	3(1)	0(1)
C1	15(1)	20(1)	16(1)	-1(1)	0(1)	-3(1)
C2	18(1)	22(1)	13(1)	-3(1)	2(1)	-3(1)
C3	18(1)	30(1)	20(1)	-3(1)	-1(1)	-8(1)
C4	26(1)	37(1)	16(1)	-7(1)	2(1)	-9(1)
C5	13(1)	19(1)	13(1)	0(1)	0(1)	-2(1)
C6	14(1)	20(1)	16(1)	0(1)	0(1)	-2(1)
C7	14(1)	28(1)	21(1)	-5(1)	1(1)	1(1)
C8	17(1)	34(1)	19(1)	-3(1)	6(1)	-7(1)
C9	23(1)	26(1)	18(1)	3(1)	2(1)	-8(1)
C10	17(1)	21(1)	19(1)	3(1)	1(1)	-3(1)
C11	22(1)	21(1)	27(1)	3(1)	2(1)	3(1)
C12	36(1)	23(1)	34(1)	11(1)	6(1)	4(1)
C13	15(1)	19(1)	13(1)	-2(1)	3(1)	-2(1)
C14	18(1)	19(1)	17(1)	-1(1)	2(1)	1(1)
C15	16(1)	28(1)	21(1)	-2(1)	5(1)	2(1)
C16	18(1)	31(1)	19(1)	-2(1)	5(1)	-6(1)
C17	22(1)	23(1)	14(1)	2(1)	1(1)	-5(1)
C18	16(1)	20(1)	14(1)	-1(1)	0(1)	0(1)
C19	25(1)	24(1)	27(1)	3(1)	4(1)	6(1)
C20	22(1)	24(1)	26(1)	5(1)	0(1)	3(1)
C21	18(1)	31(1)	21(1)	3(1)	2(1)	-1(1)
Cl2	26(1)	31(1)	23(1)	-4(1)	8(1)	-6(1)
Cl3	32(1)	39(1)	25(1)	11(1)	-2(1)	-4(1)

Table S-17. Hydrogen coordinates (x 10 ^{4}) and isotropic displacement parameters ($Å^2$ x 10^3) for **6**.

	x	y	z	U(eq)
H3A	6979	3689	4727	34
H3B	6910	3023	5659	34
H3C	6659	4142	5601	34
H4A	5365	2944	6674	40
H4B	5661	3998	6462	40
H4C	6036	3026	6725	40
H7A	7547	1451	2476	25
H8A	7651	2863	1520	28
H9A	7084	4272	1695	27
H11A	6411	858	3726	35
H11B	6898	1225	4471	35
H11C	7049	524	3597	35
H12A	5865	4434	2767	46
H12B	6361	5147	2438	46
H12C	6319	4870	3535	46
H15A	3511	2970	5835	26
H16A	3560	1475	6706	27
H17A	4362	471	6712	23
H19A	4390	3755	4139	38
H19B	4533	4365	5099	38
H19C	3893	4143	4768	38
H20A	5472	700	5232	36
H20B	5251	62	6100	36
H20C	5626	1049	6299	36
H21A	6588	1158	1143	27
H21B	6441	440	252	27

Table 18. Torsion angles [°] for **6.**

N1-Rh1-Cl1-Rh1#1	89.4(5)	N1-C5-C6-C7	175.85(15)
N2-Rh1-Cl1-Rh1#1	154.60(4)	C10-C5-C6-C11	-179.05(16)
Cl1#1-Rh1-Cl1-Rh1#1	-30.09(2)	N1-C5-C6-C11	-3.0(2)
N2-Rh1-N1-C1	-2.24(13)	C5-C6-C7-C8	-1.0(3)
Cl1-Rh1-N1-C1	63.8(5)	C11-C6-C7-C8	177.80(17)
Cl1#1-Rh1-N1-C1	-177.24(12)	C6-C7-C8-C9	1.2(3)
N2-Rh1-N1-C5	178.37(13)	C7-C8-C9-C10	-0.1(3)
Cl1-Rh1-N1-C5	-115.6(5)	C6-C5-C10-C9	1.3(3)
Cl1#1-Rh1-N1-C5	3.37(12)	N1-C5-C10-C9	-174.77(15)
N1-Rh1-N2-C2	1.60(13)	C6-C5-C10-C12	-178.76(17)
Cl1-Rh1-N2-C2	-173.93(13)	N1-C5-C10-C12	5.2(3)
Cl1#1-Rh1-N2-C2	56.8(5)	C8-C9-C10-C5	-1.1(3)
N1-Rh1-N2-C13	-169.23(14)	C8-C9-C10-C12	178.93(18)
Cl1-Rh1-N2-C13	15.24(14)	C2-N2-C13-C14	102.72(19)
Cl1#1-Rh1-N2-C13	-114.0(4)	Rh1-N2-C13-C14	-86.50(18)
C5-N1-C1-C2	-178.15(15)	C2-N2-C13-C18	-77.7(2)
Rh1-N1-C1-C2	2.5(2)	Rh1-N2-C13-C18	93.12(16)
C5-N1-C1-C3	-0.8(3)	C18-C13-C14-C15	1.5(3)
Rh1-N1-C1-C3	179.84(14)	N2-C13-C14-C15	-178.89(15)
C13-N2-C2-C1	170.51(15)	C18-C13-C14-C19	-179.60(17)
Rh1-N2-C2-C1	-0.8(2)	N2-C13-C14-C19	0.0(3)
C13-N2-C2-C4	-8.2(3)	C13-C14-C15-C16	-0.1(3)
Rh1-N2-C2-C4	-179.47(15)	C19-C14-C15-C16	-178.95(18)
N1-C1-C2-N2	-1.0(2)	C14-C15-C16-C17	-1.0(3)
C3-C1-C2-N2	-178.47(17)	C15-C16-C17-C18	0.6(3)
N1-C1-C2-C4	177.66(17)	C16-C17-C18-C13	0.8(3)
C3-C1-C2-C4	0.2(3)	C16-C17-C18-C20	179.16(17)
C1-N1-C5-C10	-90.1(2)	C14-C13-C18-C17	-1.9(3)
Rh1-N1-C5-C10	89.27(17)	N2-C13-C18-C17	178.53(15)
C1-N1-C5-C6	93.7(2)	C14-C13-C18-C20	179.75(17)
Rh1-N1-C5-C6	-86.93(17)	N2-C13-C18-C20	0.1(2)
C10-C5-C6-C7	-0.2(3)		

Symmetry transformations used to generate equivalent atoms: #1 -x+1,y,-z+1/2
